# Impact of Quantity and Type of Dietary Protein on Cardiovascular Disease Risk Factors Using Standard and Network Meta-analyses of Randomized Controlled Trials

**DOI:** 10.1093/nutrit/nuae086

**Published:** 2024-07-16

**Authors:** Yueying Yao, Vanessa Huang, Vernice Seah, Jung Eun Kim

**Affiliations:** Department of Food Science and Technology, National University of Singapore, 117543 Singapore; Department of Food Science and Technology, National University of Singapore, 117543 Singapore; Department of Food Science and Technology, National University of Singapore, 117543 Singapore; Department of Food Science and Technology, National University of Singapore, 117543 Singapore

**Keywords:** dietary protein, cardiovascular disease risk factors, blood pressure, lipid-lipoprotein profile, flow-mediated dilation

## Abstract

**Context:**

Higher protein diets (HPDs) have shown favorable outcomes on weight maintenance and body-composition management; however, their protective effects against cardiovascular diseases (CVDs) remain uncertain and contentious. Furthermore, it is important to consider the influence of other macronutrients in the diet and type of dietary protein when studying HPDs, because this aspect has been overlooked in previous studies.

**Objective:**

We assessed the impacts of quantity and type of dietary protein on CVD risk factors.

**Data Sources:**

A database search was conducted in PubMed, Embase, CINAHL, Web of Science, and Cochrane Library and a total of 100 articles met the eligibility criteria.

**Data Extraction:**

Extracted data from 100 articles were analyzed using standard meta-analysis, and 41 articles were also analyzed using network meta-analysis.

**Data Analysis:**

In the standard meta-analysis, an HPD had significant favorable effects on systolic blood pressure (SBP) (mean difference [MD] = −1.51 mmHg; 95% CI: −2.77, −0.25), diastolic blood pressure (DBP) (MD = −1.08 mmHg; 95% CI: −1.81, −0.35), and flow-mediated dilation (MD = 0.78%; 95% CI: 0.09, 1.47) compared with lower protein diets. The further network meta-analysis supported that the high-protein, high-carbohydrate, low-fat diet was the most recommended diet to ensure a maximum decrease in SBP, DBP, total cholesterol (TC), and low-density-lipoprotein cholesterol (LDL-C). In comparison to animal-protein–rich diets, plant-protein–rich diets (PPRs) exhibited a significant favorable effects on improving TC (MD = −0.12 mmol/L; 95% CI: −0.19, −0.05), triglyceride (MD = −0.05 mmol/L; 95% CI: −0.09, −0.01), LDL-C (MD = −0.11 mmol/L; 95% CI: −0.18, −0.04), and high-density-lipoprotein cholesterol (MD = 0.03 mmol/L; 95% CI: 0.02, 0.04) levels.

**Conclusion:**

Consumption of HPDs and PPRs supports improvements in vascular health and lipid-lipoprotein profiles, respectively. Furthermore, macronutrient composition should be carefully designed in the dietary approach to maximize the effectiveness of HPDs in improving CVD risk factors.

**Systematic Review Registration:**

PROSPERO registration no. CRD42022369931.

## INTRODUCTION

Cardiovascular disease (CVD) is a leading cause of mortality, representing 30% of all global deaths.^1^ Lifestyle modifications, including dietary interventions, are recognized as the primary nonpharmacological approach for the prevention of CVD by effectively addressing major modifiable risk factors, such as hypertension and dyslipidemia.^1^^,^^2^ For example, studies have suggested that diets low in dietary fat or carbohydrate content could reduce CVD risk, particularly by improving blood lipid-lipoprotein profiles.^3^^,^^4^ More recently, dietary protein has received increased attention for its role in preventing CVD due to its potential in blood pressure regulation and endothelial function improvement.^5^^,^^6^ Several researchers have assembled evidence indicating an inverse relationship between dietary protein intake and blood pressure.^7–11^ In addition, The Nurses’ Health Study found a moderate negative association between dietary protein intake and the incidence of ischemic heart disease (26% risk reduction).^12^ In contrast, data from another prospective cohort study indicated that increased dietary protein intake is associated with an increased incidence of CVD (4%–5% increase in risk).^13^

These controversial results from different studies have recently been explained by differences in type of dietary protein.^14^ Foods with different types of dietary protein contain different nutrients or bioactive compounds and this may have beneficial or adverse effects on CVD health. For instance, cholesterol and saturated fat contained in red meat have been linked to an elevated risk of CVD,^15^ while plant sterols provided by soy have been proved to be associated with reduced CVD risk and mortality.^14^ In addition, plant- and animal-based dietary protein–rich foods provide a unique profile of amino acids, fatty acids, carbohydrates, and micronutrients, all of which can contribute to divergent impacts on cardiovascular health.^14^ Furthermore, the discrepant outcomes evident in prior research might be attributed to variations in composition of other macronutrients with similar protein diet contents.^16^^,^^17^ Researchers have found that, with similar protein intake, a significant decrease in low-density-lipoprotein cholesterol (LDL-C) concentrations was only observed in the higher-carbohydrate diet (65% vs 48%) and lower-fat diet (13% vs 33%).^17^ Collectively, disregarding the type of dietary protein and the varying composition of other macronutrients within the diet could potentially affect the intended outcomes^18^^,^^19^ For this reason, the latest research has demonstrated that assessing the health benefits of dietary protein intake on improving cardiovascular risk factors must consider the type of protein and should not ignore the impact of eliminated nutrients from the diet with increased protein consumption.

Therefore, the present study aimed to evaluate the effects of dietary protein quantity (higher protein diets [HPDs] vs lower protein diets [LPDs]) and type (plant-protein–rich diets [PPRs] vs animal-protein–rich diets [APRs]) on CVD risk factors using standard meta-analysis of randomized controlled trials (RCTs). Further network meta-analysis (NMA) was conducted to comprehensively assess and rank the effect of the composition of different macronutrients within the diet on CVD risk factors.

## METHODS

The protocol of this study was registered at the International Prospective Register of Systematic Reviews as CRD42022369931. The present systematic review, meta-regression, and meta-analysis was planned, conducted, and reported in adherence to the PRISMA (Preferred Reporting Items for Systematic Reviews and Meta-Analyses) guidelines^20^ and the PRISMA Extension Statement for conducting Network Meta-analyses.^21^ The description of the PICOS (Population, Intervention, Comparison, Outcome, and Study design) criteria used to define the research question is presented in Table 1.

**Table 1.** PICOS Criteria for Inclusion of Studies

Parameter	**Criteria**
Population	Adults with a mean age ≥19 years
Intervention	Groups that consumed a higher protein diet (>20% of total energy intake from dietary protein) and groups that consumed a plant-protein–rich diet (>50% of total protein intake from plant-based protein)
Comparator	Groups that had a lower protein diet and groups that consumed animal-protein–rich diets
Outcome	Primary outcomes include classical CVD risk factors, such as blood lipids, lipoproteins, blood pressure, flow-mediated dilation; secondary outcomes include oxidative stress and inflammation biomarkers
Study design	Randomized controlled trials
Research question	Are there difference impacts in the quantity and type of dietary protein consumption on CVD risk factors?

*Abbreviation:* CVD, cardiovascular disease.

### Search strategy and eligibility criteria

A computerized search of the literature was performed independently by a primary reviewer (V.H.) and a secondary reviewer (V.S.) using 5 online databases, including PubMed, Embase, CINAHL Plus with Full Text, Web of Science, and Cochrane Library in September 2022 and updated in June 2023. The search strategy focused on dietary protein as well as selected primary outcomes of interest. Medical subject headings (MeSH) were applied when applicable. The search strategy is detailed in **Table S1**.

To be included in standard and/or network meta-analyses, studies had to meet the following inclusion criteria: (1) human RCTs with subjects having a mean age ≥19 years, (2) RCTs that either compared the effects of HPDs with LPDs or accessed the effects of PPRs to those of APRs, (3) reported at least 1 of the primary outcomes as a dependent variable, and (4) articles published in English. In addition, RCTs that assessed acute postprandial response were excluded. Also, crossover trials were treated as parallel studies with the full number of study participants to represent the population for both HPDs /PPRs and LPDs/APRs.^22^

A high-protein diet was predefined as >20% protein (PRO) of total energy intake^23^; a low-carbohydrate diet and a moderate-carbohydrate diet were predefined as < 25% and 25%–45% carbohydrate (CHO) of total energy intake, respectively^23^; a low-fat diet was predefined as < 30% fat of total energy intake.^23^^,^^24^ Accordingly, in the NMA, the diets were further categorized into 7 groups based on different combinations of macronutrients—namely, a low-PRO, moderate-CHO, and high-fat (LMH) group; a low-PRO, high-CHO, and low-fat (LHL) group; a low-PRO, high-CHO, and high-fat (LHH) group; a high-PRO, moderate-CHO, and low-fat (HML) group; a high-PRO, moderate-CHO, and high-fat (HMH) group; a high-PRO, high-CHO, and low-fat (HHL) group; and a high-PRO, high-CHO, and high-fat (HHH) group. Similarly, if the proportion of plant-based protein exceeded that of the animal-based protein, we defined it as a PPR and vice versa.

### Data extraction and quality assessment

Data from the selected studies were also extracted by 2 independent reviewers (V.H. and V.S.). Relevant data included the following characteristics: title, name of first author, publication year, country, population size, dropout rate, RCT design and duration, washout duration (for crossover studies only), health status, mean age, gender ratio, mean baseline body mass index (BMI), specification of protein intervention (amount, form, source), macronutrient composition in their diets, mean and SD or SEM of the pre-intervention and post-intervention, and change values for selected outcome variables. Corresponding authors were contacted through e-mail to obtain additional information and missing data, and 1 author provided additional data.^25^ Any discrepancies during the screening and data-extraction process were discussed between the 2 reviewers and resolved by a third reviewer (Y.Y.).

The quality assessment of the selected studies was performed using a modified version of the Cochrane risk-of-bias tool,^22^ where 2 reviewers (V.H. and Y.Y.) independently assigned a subjective level of risk (such as low, high, or unclear) to 4 domains: random-sequence generation, allocation concealment, blinding of participants and investigator, and blinding of outcome assessors. Any disagreements were resolved by consensus. Risk of bias for NMA also included evaluation of transitivity.^26^ To assess the assumption of transitivity, the distribution of the potential effect modifiers (BMI, age, percent male, study duration, sample size) across the available direct comparisons was compared. Box plots were obtained using GraphPad Prism 9.5.1 (GraphPad Software, Boston, Massachusetts, USA) to present the transitivity data.

### Grading of the evidence

To make inferences about the quality of evidence from the NMA, the Grading of Recommendations Assessment, Development, and Evaluation (GRADE) approach, which was suggested by Salanti et al,^27^ was applied for the previous selected outcomes. Overall, GRADE specifies 4 levels of certainty of evidence: high, moderate, low, and very low.

### Data synthesis and analysis

A standard meta-analysis was performed using standard Cochrane methods, and the evidence from standard meta-analysis for protein quantity group was further analyzed using NMA.^28^ Data were managed and analyzed with the use of STATA/IC version 13 (StataCorp LP, College Station, TX, USA) for all analyses.

#### Standard meta-analysis

The generic inverse variance method with random-effects models was applied to synthesize the overall effect estimates of HPD and LPD or PPR and APR on the following: (1) systolic blood pressure (SBP; mmHg), (2) diastolic blood pressure (DBP; mmHg), (3) total cholesterol (TC; mmol/L), (4) triglycerides (TG; mmol/L), (5) LDL-C (mmol/L), (6) high-density-lipoprotein cholesterol (HDL-C; mmol/L), (7) flow-mediated dilation (FMD; %), (8) C-reactive protein (CRP; mg/L), (9) interleukin (IL)-6 (ng/L), and (10) tumor necrosis factor-α (TNF-α; ng/L). For each selected outcome, we extracted or calculated the change values from baseline (pre-intervention) to the end of the study (post-intervention), along with their corresponding SDs, using the available data.^29^^,^^30^ Then, the absolute mean differences (MDs) and the SDs for MDs between HPDs and LPDs or PPRs and APRs were calculated and statistics was based on published formulas.^29^^,^^30^ The pooled estimates were expressed and presented as MDs with their 95% CIs. Interstudy heterogeneity was assessed and quantified by the *I^2^* statistic. To explore the sources of heterogeneity, sensitivity analyses were subsequently conducted by excluding every single group or trial and repeating the meta-analysis.^31^ Additionally, the presence of publication bias was examined via a visual inspection of the funnel plot coupled with an Egger’s regression test.^32^

#### Meta-regression

Meta-regression analyses were conducted with the MD and the SD to determine the dose-dependent association between the PRO % of energy (E%) during the intervention and the change values of each selected outcome. The MD was obtained by calculating the mean changes of the selected outcome values observed from the intervention and baseline. A random-effects model was applied using a univariable analysis. Statistical significance was accepted at *P *<* *.05.

#### Network meta-analysis

A frequentist NMA was performed using a multivariate meta-analysis model and the “network” suite of commands available in STATA (StataCorp, College Station, TX, USA).^33^ Network plots were performed for each selected outcome to illustrate the interactions among the studies included in the NMA and to show the available direct comparisons between dietary interventions.^33^ The inconsistency in the data was checked using both global and local approaches. The global approach tests for overall inconsistency from all possible sources in the whole network simultaneously using a design-by-treatment interaction model, while the side-splitting approach detects comparisons for which direct estimates disagree with indirect evidence from the entire network.^34^^,^^35^ If inconsistency was suggested, the sensitivity analyses were conducted by removing every single group or trial to explore the sources of heterogeneity.^31^ The network funnel plots were created to visually evaluate the publication bias using the criterion of symmetry.^36^ Afterwards, random-effects NMAs for each selected outcome were performed in order to estimate all possible pairwise relative effects. Ranking probabilities of the different treatments for each outcome were estimated using the surface under the cumulative ranking (SUCRA) curve. The larger the SUCRA values, the better the rank of the intervention (100% = best, 0% = worst).^33^

## RESULTS

### Results of the literature search

**Figure 1.**
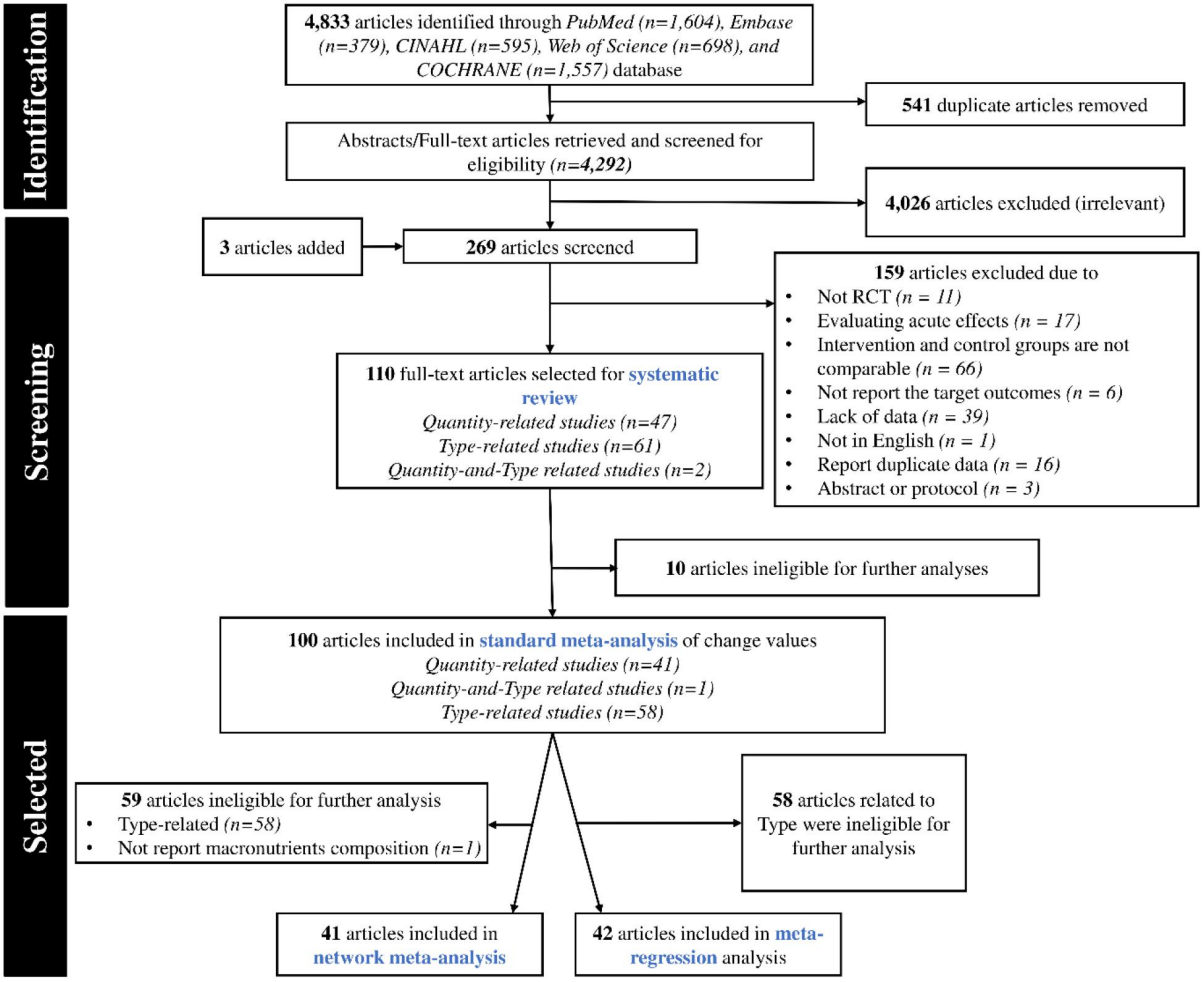
Flow Diagram of the Literature Search Process. Abbreviation: RCT, randomized controlled trial

The study selection flowchart is illustrated in Figure 1. A total of 4833 articles were identified and, after the exclusion of duplicates (*n* = 541), the titles and abstracts of 4292 articles were screened. Another 4026 articles were excluded due to irrelevance, while 3 articles were added from other reviews; thus, a total of 269 articles were reviewed in full text. During the screening, a total of 159 articles were excluded according to the inclusion and exclusion criteria, including 11 non-RCTs, 17 evaluating acute effects, 66 with intervention and control groups that were not comparable, 6 not reporting the target outcomes, 39 lacking data, 1 not in English, 16 reporting duplicate data, and 3 consisting solely of abstracts or protocols, of which 100 articles provided data for inclusion in the standard meta-analysis (including 42 articles for the quantity group and 59 articles for the type group). Furthermore, 41 articles were included in the NMA because, through our sorting method, we were able to categorize the intervention and control groups within each of these articles into different groups based on varying combinations of macronutrients.

### Characteristics of selected studies

Characteristics of articles included for protein quantity and type are summarized in Tables S2 and S3, respectively. Forty-nine quantity-related articles included a total number of 4180 participants (35% male) and the mean age and BMI of the participants were 46 years (range: 23 to 77 years) and 31.2 kg/m^2^ (range: 23.0 to 45.8 kg/m^2^), respectively. Only 7 articles included healthy participants and the rest of studies included participants with obesity or overweight, as well as hyperinsulinemia, hypertension, hypercholesterolemia, or hyperlipidemia. The protein intake in the LPD ranged from 11% to 20%, while the protein intake ranged from 20% to 40% in the HPD. The study duration ranged from 4 to 238 weeks.

With respect to type-related articles, 63 articles with a total of 4061 participants (38% male) were included and the mean age and BMI of the participants were 50 years (range: 20 to 66 years), and 26.9 kg/m^2^ (range: 20.9 to 36.1 kg/m^2^), respectively. Studies included healthy participants as well as obese or overweight, diabetic, hypercholesterolemic, hyperlipidemic, and hypertensive participants of all ages. The plant-sourced proteins used in the articles were mainly from legumes, such as soy, lupin, and pea proteins, and the animal-sourced proteins included dairy, meat, and egg. The duration of the studies ranged from 2 to 238 weeks.

### Network diagrams

**Figure S1** displays the network diagrams for each selected outcome of all available direct comparisons between pairs of intervention used in NMA. The largest number of articles compared the impact of the HMH with the LHH (*n *= 18) on blood pressure, whereas the number of articles comparing the effects of the HMH with the LHH (*n* = 16) and the HML with the LHL (*n* = 16) on lipid-lipoprotein profiles was comparably large.

### Standard meta-analysis, meta-regression, and NMA

All prespecified outcomes were analyzed in meta-analysis for dietary protein quantity, whereas 8 outcomes were analyzed for dietary protein type. Also, meta-regression and NMA were performed for only 6 outcomes due to the limited data. All trends observed below were robust and largely stable to sensitivity analysis (Tables S4–S6).

#### Results of protein quantity

##### Standard meta-analysis and meta-regression

**Figure 2.**
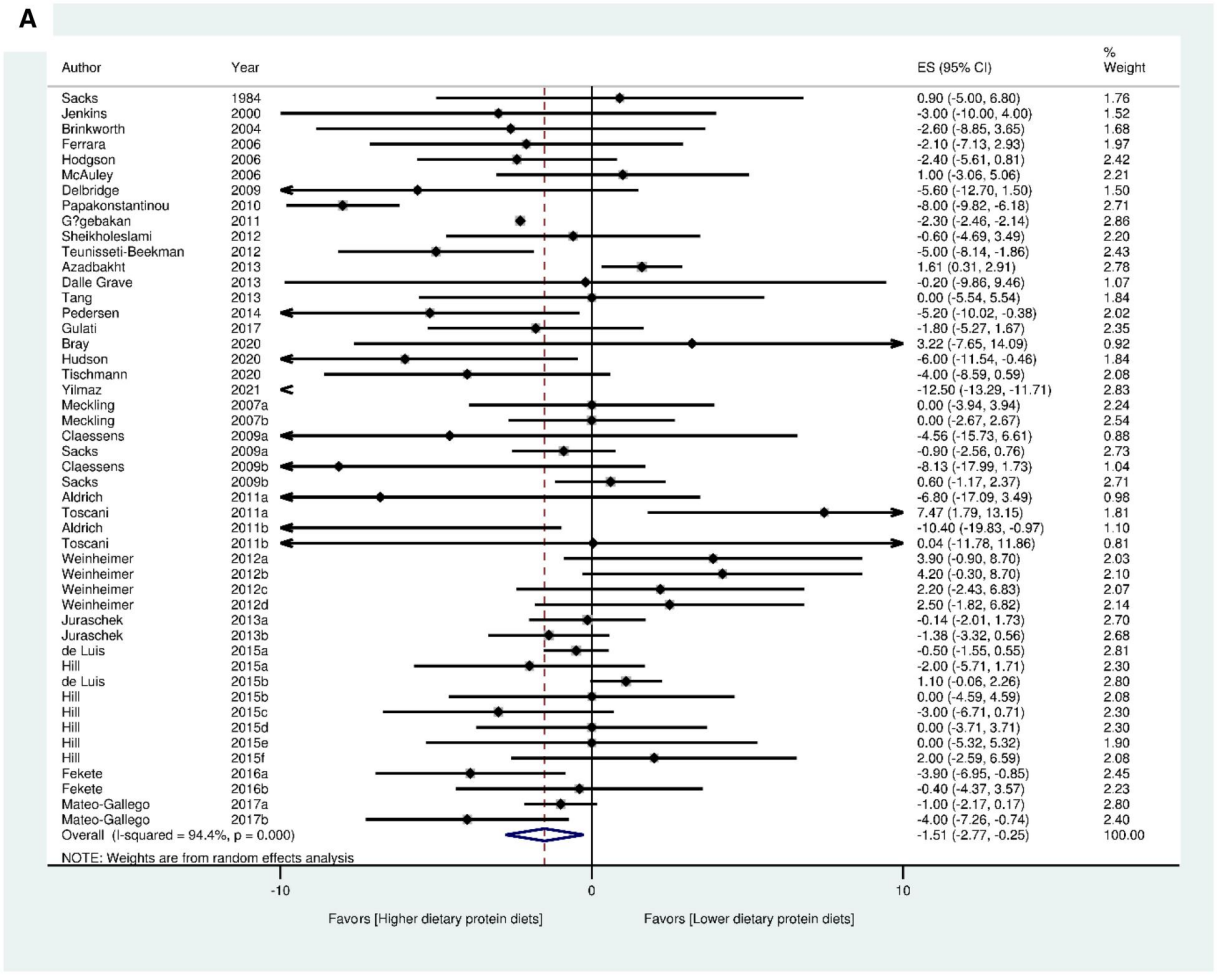

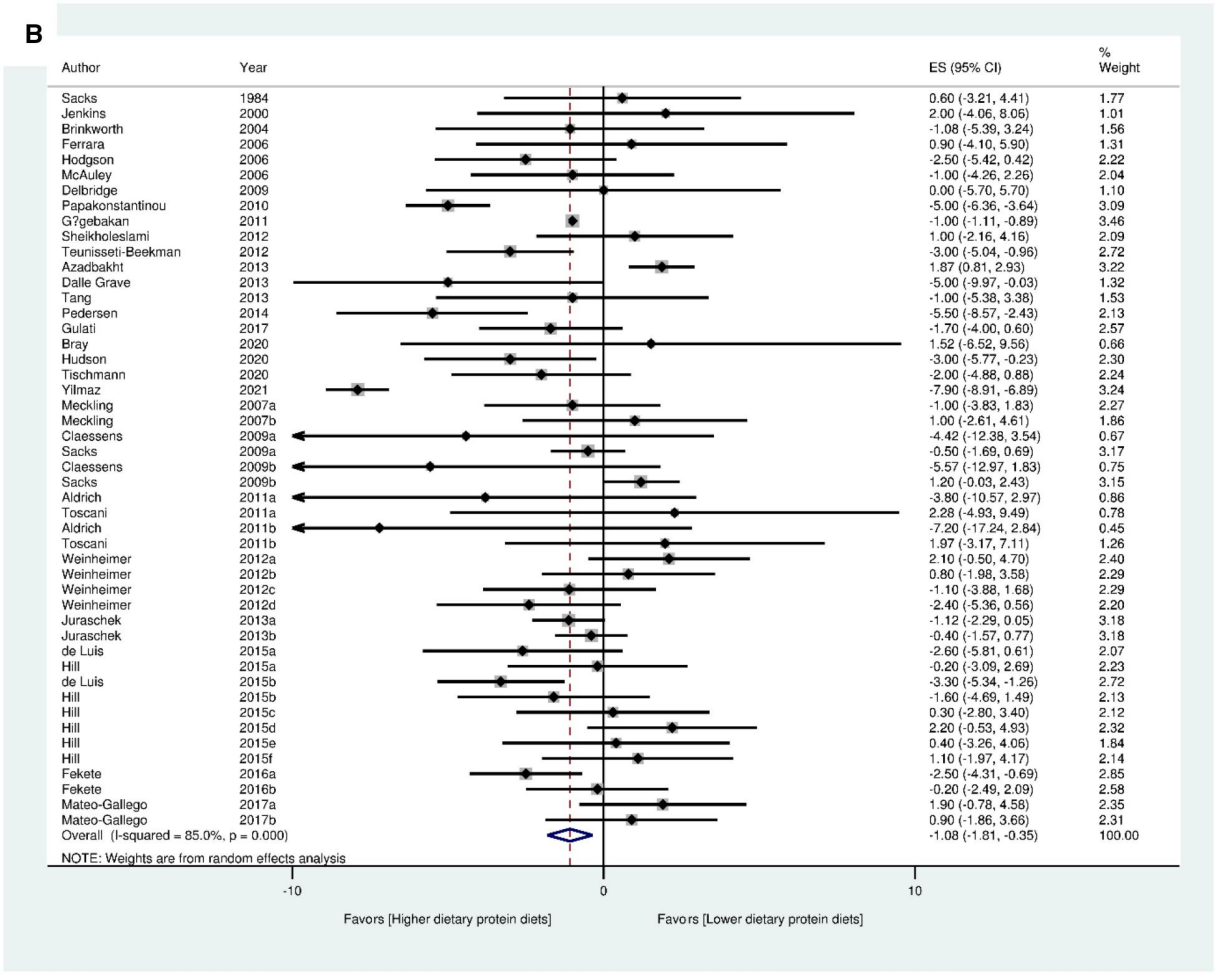

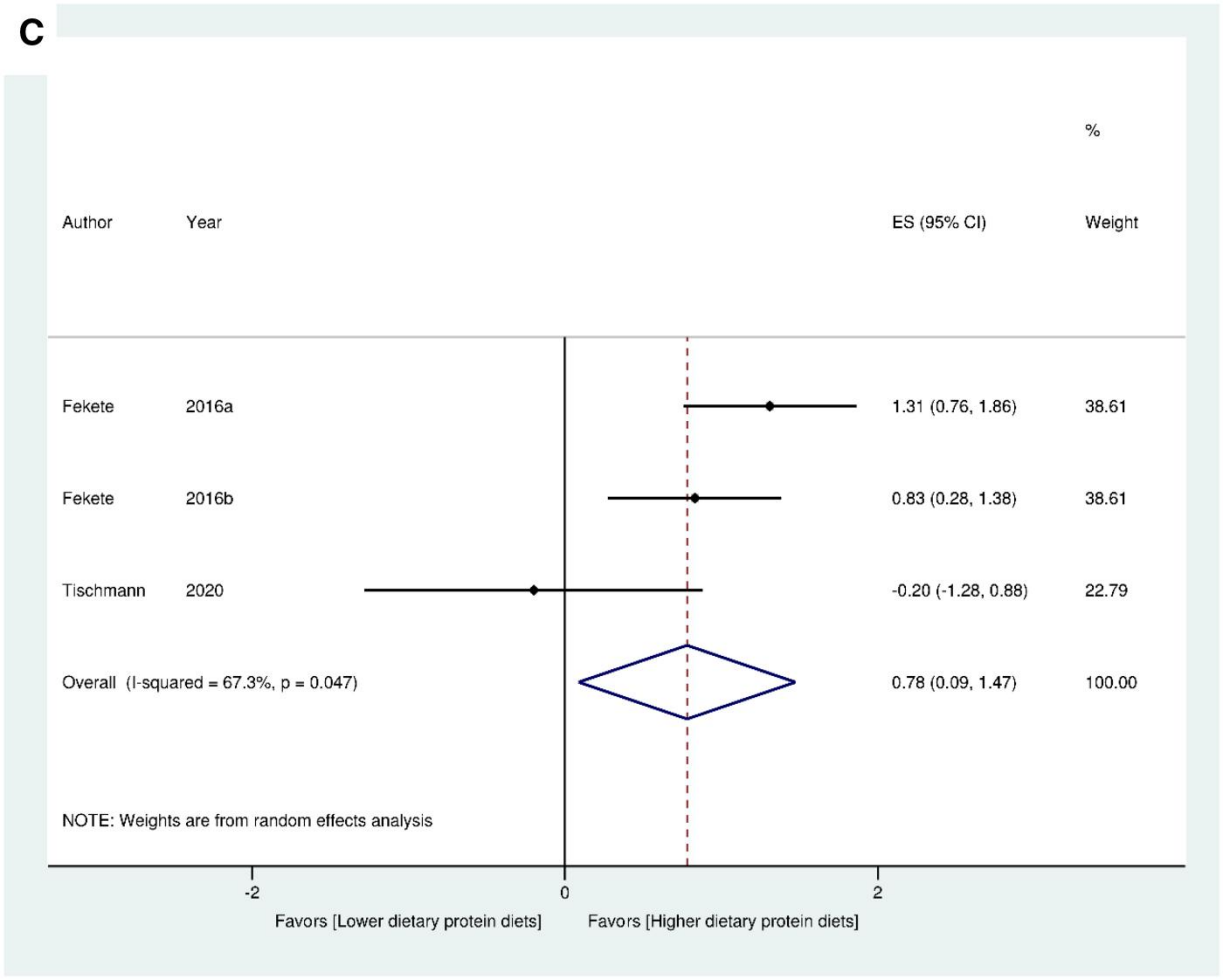
Forest Plots for SBP (A), DBP (B), and FMD (C) in Standard Meta-analysis for Quantity-Related Studies. Abbreviations: DBP, diastolic blood pressure; ES, effect size; FMD, flow-mediated dilation; SBP, systolic blood pressure

In the standard meta-analysis, the HPD significantly improved vascular health by reducing SBP (MD_HPD vs LPD_ = -1.51 mmHg; 95% CI: -2.77, -0.25) (31 articles with 48 trials) and DBP (31 articles with 48 trials) (MD_HPD vs LPD_ = -1.08 mmHg; 95% CI: -1.81, -0.35), as well as enhancing FMD (2 articles with 3 trials) (MD_HPD vs LPD_ = 0.78%; 95% CI: 0.09, 1.47) in comparison to the LPD (Figure 2). Further analysis through meta-regression revealed significant inverse associations between protein intake and changes in SBP (-0.192 mmHg/PRO E%; *P *=* *.013) and DBP values (-0.096 mmHg/PRO E%; *P *=* *.032) (Table S7 and Figure S2).

Although the HPD induced a reduction in TC (38 articles with 56 trials) (MD_HPD vs LPD_ = -0.09 mmol/L; 95% CI: -0.15, -0.04), no change was observed in other blood lipid-lipoprotein profiles (TG [37 articles with 54 trials), LDL-C [37 articles with 54 trials], HDL-C [39 articles with 57 trials]) and inflammatory markers (CRP: 11 articles with 22 trials; IL-6: 3 articles with 4 trials; TNF-α: 3 articles with 4 trials) (Figure S3). No significant association between protein intake and changes in the lipid-lipoprotein profile was observed.

##### Network meta-analysis

The main results of the NMA are presented as league tables (Tables S8–S13) and a relative ranking table (Table 2). Sensitivity analyses were only performed for HDL-C in the NMA as no inconsistency was observed for the rest of the comparisons.

**Table 2.** Relative Ranking of Diets With a Different Macronutrient Composition for Selected Outcomes

Treatment group	Ranking					
	SBP	DBP	TC	TG	LDL-C	HDL-C
LMH	13.70	28.70	13.80	20.30	13.80	14.50
LHL	0.30	0.20	0.10	3.80	0.60	1.10
LHH	0.00	0.00	0.00	0.00	0.00	**60.70**
HML	30.80	5.80	4.60	**47.10**	8.50	9.40
HMH	6.70	24.10	14.60	15.50	5.90	10.30
HHL	**34.40**	**32.70**	**58.00**	1.00	**69.10**	0.70
HHH	14.10	8.50	8.90	12.20	2.10	3.40

The values represent the SUCRA for all outcomes, and the bolded value represent the best ranking for every outcome (eg, the HHL group was ranked as the best dietary intervention group for reducing SBP, SUCRA [34.40%]).

Abbreviations: DBP, diastolic blood pressure; HDL-C, high-density-lipoprotein cholesterol; HHH, high-protein, high-carbohydrate, high-fat diet; HHL, high-protein, high-carbohydrate, low-fat diet; HMH, high-protein, moderate-carbohydrate, high-fat diet; HML, high-protein, moderate-carbohydrate, low-fat diet; LDL-C, low-density-lipoprotein cholesterol; LHH, low-protein, high-carbohydrate, high-fat diet; LHL, low-protein, high-carbohydrate, low-fat diet; LMH, low-protein, moderate-carbohydrate, high-fat diet; SBP, systolic blood pressure; SUCRA, surface under the cumulative ranking curves; TC, total cholesterol; TG, triglyceride.

For blood pressure, the HHL showed the highest probability to be the best treatment in improving SBP (SUCRA: 34.40%) and DBP (SUCRA: 32.70%), whereas the LHH was found to be the least effective dietary approach on both SBP (SUCRA: 0.00%) and DBP (SUCRA: 0.00%) among all interventions. From the league tables, the HMH (MD_HMH vs LHH_ = -2.68 mmHg; 95% CI: -4.42, -0.93) and HHL (MD_HHL vs LHH_ = -3.99 mmHg; 95% CI: -7.58, -0.40) exhibited a significant decrease in SBP; the HMH also showed a significant decrease in DBP (MD_HMH vs LHH_ = -1.76 mmHg; 95% CI: -3.03, -0.50). Additionally, among HPDs, low-fat diets showed greater impacts on SBP reduction than the high-fat diets (MD_HHH vs HHL_ = 1.12 mmHg, 95% CI: -3.08, 5.33; MD_HHH vs HML_ = 0.94 mmHg, 95% CI: -3.22, 5.09; MD_HMH vs HML_ = 1.12 mmHg, 95% CI: -2.97, 5.22).

With regard to the blood lipid-lipoprotein profile, the HHL had the highest SUCRA for TC (58.00%) and LDL-C reduction (69.10%), but generated the lowest SUCRA for enhancing HDL-C (0.70%), and the highest SUCRA for TG reduction (47.10%) was achieved by the HML. As shown in the league tables, significant favorable effects of the HMH (MD_HMH vs LHH_ = -0.15 mmol/L; 95% CI: -0.24, -0.05) and HHL (MD_HHL vs LHH_ = -0.21 mmol/L; 95% CI: -0.38, -0.03) on TC, the HML (MD_HML vs LHH_ = -0.22 mmol/L; 95% CI: -0.43, -0.02) and HMH (MD_HMH vs LHH_ = -0.17 mmol/L; 95% CI: -0.30, -0.04) on TG, and the HHL (MD_HHL vs LHH_ = -0.19 mmol/L; 95% CI: -0.35, -0.02) on LDL-C were observed. However, the HHL showed a significant HDL-C reduction impact when compared with the LHH (MD_HHL vs LHH_ = -0.06 mmol/L; 95% CI: -0.12, -0.01).

#### Results of protein type

**Figure 3.**
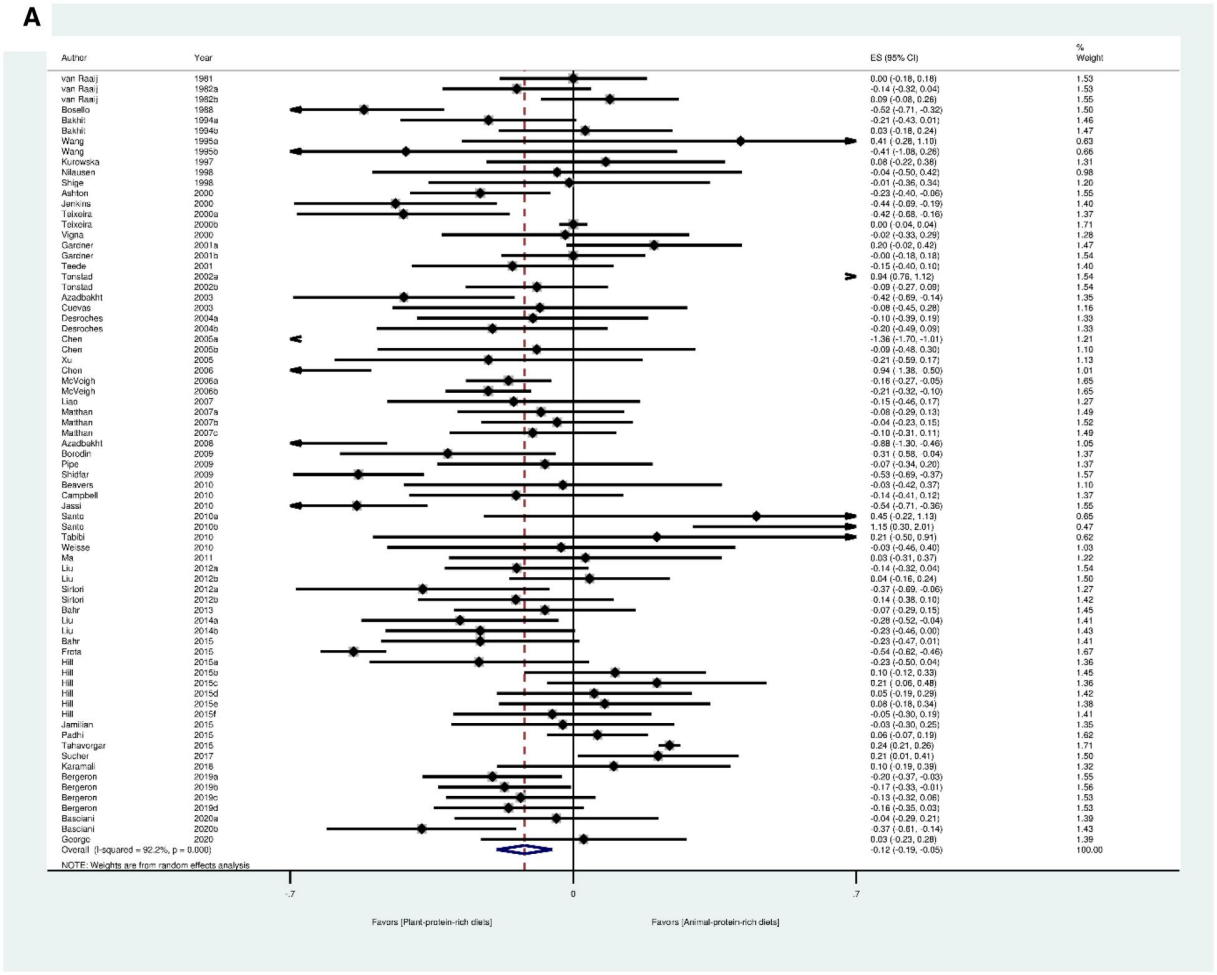

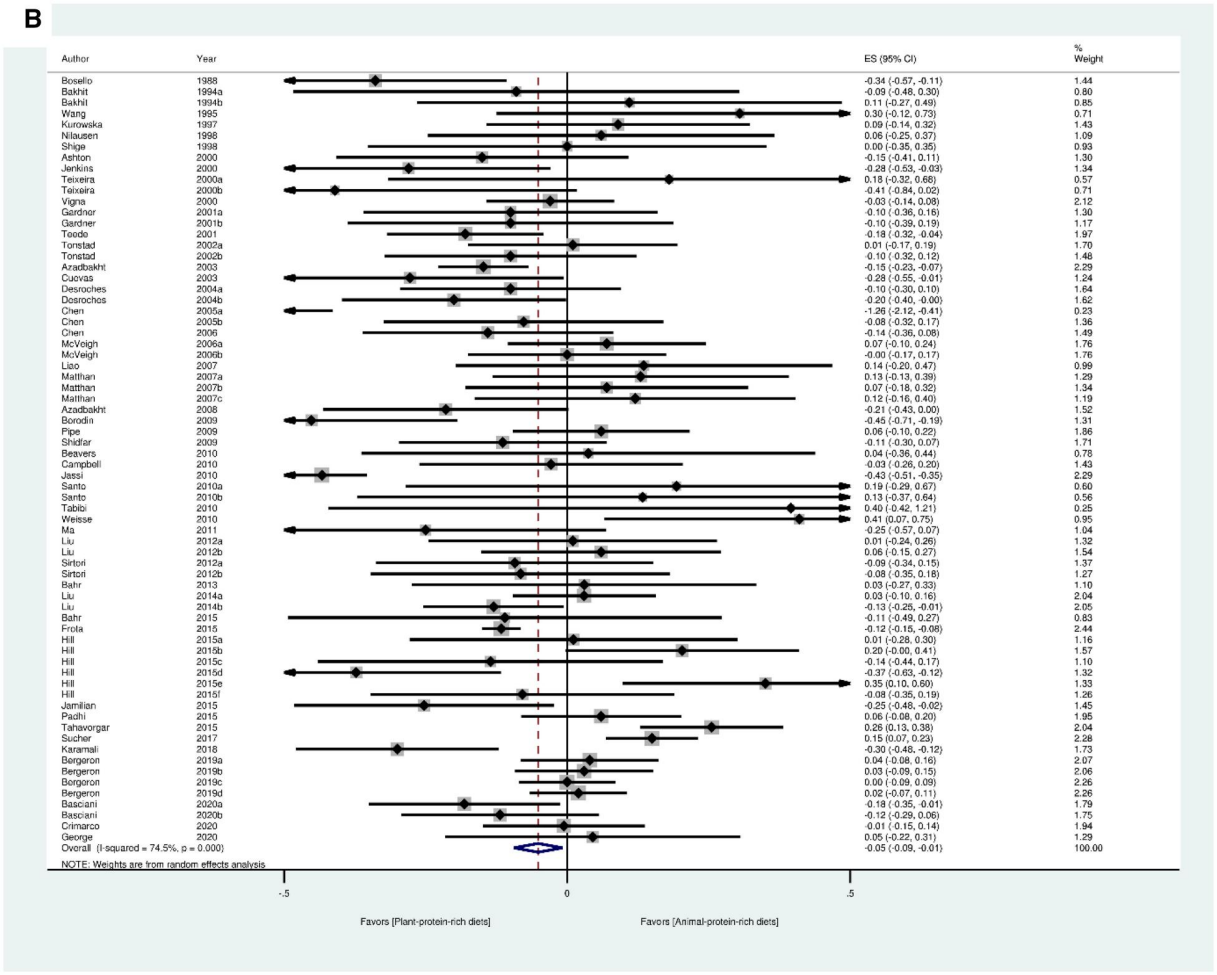

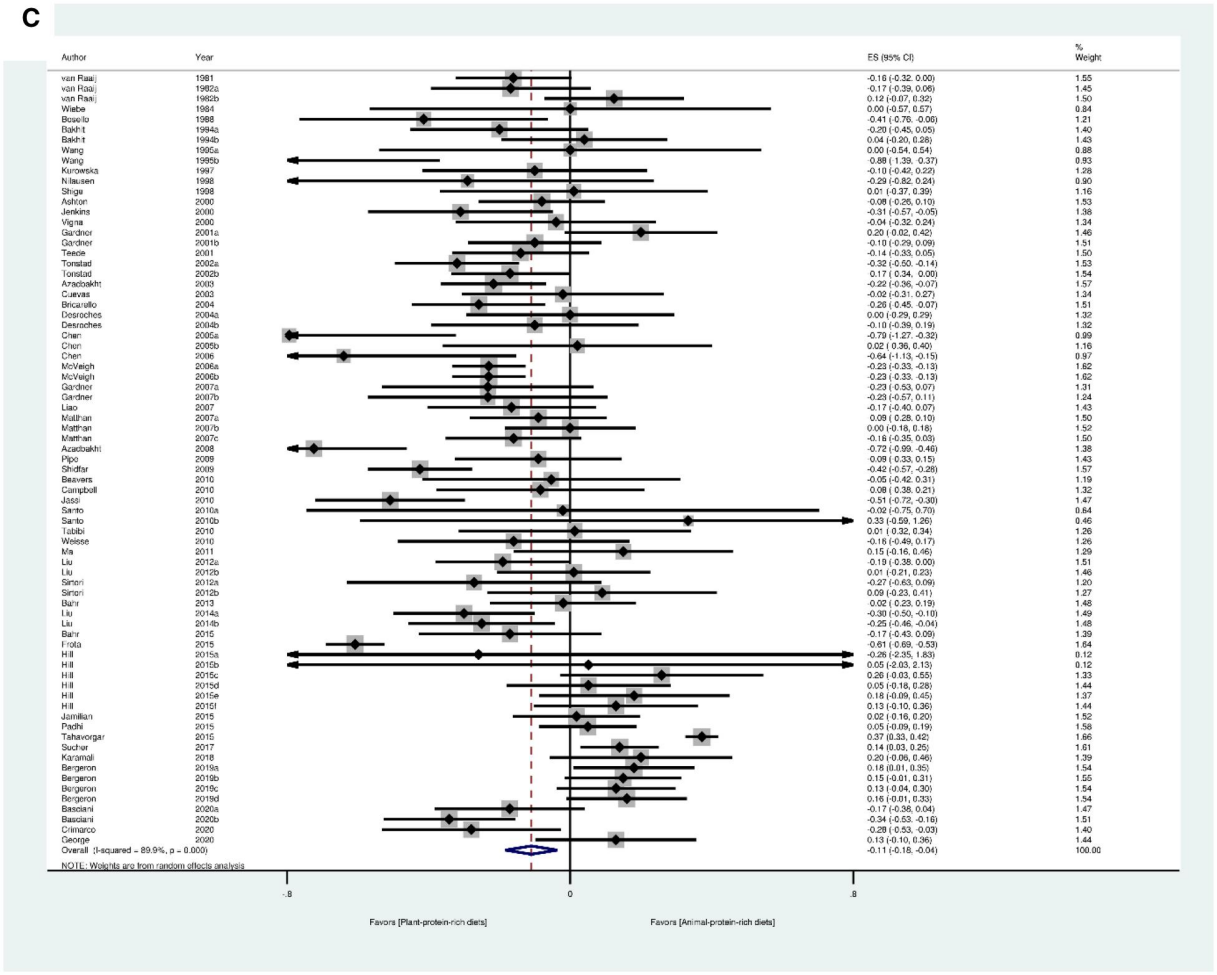

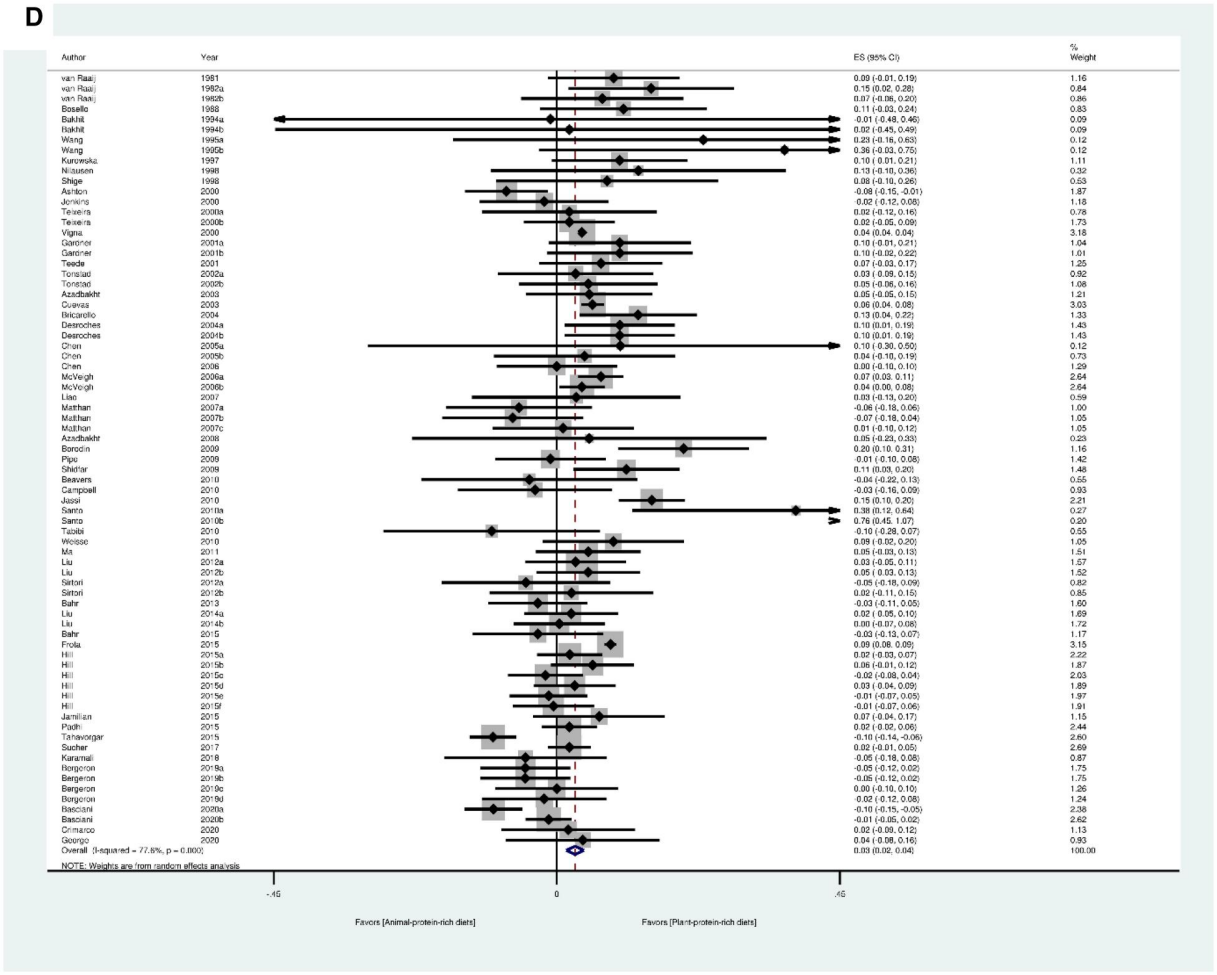
Forest Plots for TC (A), TG (B), LDL-C (C), and HDL-C (D) in Standard Meta-analysis for Type-Related Studies. Abbreviations: ES, effect size; HDL-C, high-density-lipoprotein cholesterol; LDL-C, low-density-lipoprotein cholesterol; TC, total cholesterol; TG, triglyceride

In the standard meta-analysis, the PPR significantly improved lipid-lipoprotein profiles, notably through the reduction of TC (50 articles with 74 trials) (MD_PPR vs APR_ = -0.12 mmol/L; 95% CI: -0.19, -0.05), TG (47 articles with 70 trials) (MD_PPR vs APR_ = -0.05 mmol/L; 95% CI: -0.09, -0.01), LDL-C (50 articles with 74 trials) (MD_PPR vs APR_ = -0.11 mmol/L; 95% CI: -0.18, -0.04), and an elevation of HDL-C (51 articles with 75 trials) (MD_PPR vs APR_ = 0.03 mmol/L; 95% CI: 0.02, 0.04) (Figure 3). However, there were no beneficial effects of PPR compared with APR on blood pressure (SBP: 20 articles with 31 trials; DBP: 20 articles with 31 trials), FMD (2 articles with 3 trials), and inflammatory factors (CRP: 13 articles with 25 trials; TNF-α: 2 articles with 2 trials) (Figure S4).

### Risk of bias

Table S14 shows the individual Cochrane risk-of-bias assessments for all 110 articles included in this study. Collectively, 10 out of 36 articles with a crossover study design were judged to be at high risk of bias due to period and carryover effects. Four articles were deemed to have a high risk of bias in terms of missing outcome data,^37–40^ and 4 articles were judged to be at high risk of bias for measurement of the outcome.^41–44^ Overall, 51 articles (46%) were judged to be at low risk of bias, while 17 articles (15%) were rated as high risk of bias for ≥1 domain.

Transitivity analyses showed minor differences among studies for participants’ characteristics (ie, BMI, age, % male) and RCT characteristics (ie, study duration, sample size) (Figure S5).

### Inconsistency

For comparisons in the network meta-analysis, the side-splitting approach suggested some inconsistency for HDL-C (Table S15) in the comparisons of HML with LHH and HML with LHL. The design-by-treatment model showed no significant inconsistency for SBP (*P *=* *.55), DBP (*P *=* *.30), TC (*P *=* *.43), TG (*P *=* *.75), LDL-C (*P *=* *.63), and HDL-C (*P *=* *.19).

### Publication bias

Publication bias analysis for FMD, IL-6, and TNF-α was not performed, as there were fewer than 10 direct comparisons available for each of them. In addition, no evidence for publication bias was found in the standard meta-analysis for the outcomes for quantity-related studies, except for TC (Egger’s test *P *=* *.004) (Figure S6). With regard to for type-related studies, the funnel plots for SBP (Egger’s test *P *=* *.026), DBP (Egger’s test *P *=* *.002), TC (Egger’s test *P *=* *.000), and LDL-C (Egger’s test *P *=* *.007) in standard meta-analysis appears to be asymmetric (Figure S7). Meanwhile, Figure S8 displays the comparison-adjusted funnel plots including all studies of the NMA for each selected outcome. There was no visual evidence of funnel-plot asymmetry for each selected outcome.

### GRADE assessment

The certainty of evidence for all outcomes was rated moderate or high for most comparisons (Table S16). However, some articles generated low quality of evidence, largely driven by the high degree of inconsistency resulting from the variability of participant characteristics and interventions included in the study.

## DISCUSSION

Higher protein diets have been shown to have beneficial effects on weight maintenance and body-composition management^45^; however, their protective effects against CVD are still not clearly defined and remain controversial. In this study, the favorable effects of HPD intake on lowering blood pressure and improving FMD were observed without altering lipid-lipoprotein profiles. The NMA results further reinforced these findings, highlighting the HHL diet as the most potent dietary approach in reducing SBP, DBP, TC, and LDL-C levels. In addition, the PPR showed significant enhancements in the lipid-lipoprotein profile compared with the APR, particularly in reducing TC, TG, and LDL-C levels while simultaneously increasing HDL-C levels.

The blood pressure–lowering effect of an HPD is consistent with previous systematic reviews and meta-analyses conducted in RCTs and observational studies.^46^^,^^47^ With regard to the blood pressure management effect, the possible explanation is that an HPD, irrespective of protein type, can provide antihypertensive effects due to the bioactive peptides it contains,^48–50^ such as the tryptophan-containing dipeptides isoleucine-tryptophan and tryptophan-leucine.^51^ These bioactive peptides have been shown to inhibit the activity of angiotensin converting enzyme (ACE), a component of the renin-angiotensin system that mediates systemic hypertension.^49^ Martin et al^48^ also indicated that these bioactive peptides with ACE inhibitory activity could enter the human bloodstream without being degraded into amino acids. In addition, an HPD showed an improvement in FMD, which is the gold standard for evaluating endothelial function.^52^ Researchers have demonstrated that ACE inhibitors could improve endothelial dysfunction by enhancing the release of nitric oxide from endothelial cells to induce vasodilation^53–55^ and the capability of inhibiting ACE activity with an HPD may explain the improvement in FMD. The progression of atherosclerosis and endothelial dysfunction has also been linked to cell adhesion molecules.^56^ During atherosclerosis plaque formation, circulatory soluble adhesion molecules (such as soluble intercellular adhesion molecule-1 [sICAM-1] and soluble vascular adhesion molecule-1 [sVCAM-1]) become activated.^57^ Research has shown a notable reduction in sICAM-1 and sVCAM-1 levels after higher protein consumption,^49^^,^^58^ implying that the positive effects of higher protein consumption on adhesion molecules could potentially serve as a mechanism underlying improvements in vascular reactivity.

However, the limited improvement in lipid-lipoprotein profiles by an HPD has been observed and this is possibly because the changes in blood lipid-lipoprotein levels are largely dependent on the type of protein rather than the amount.^6^ In terms of inflammatory biomarkers, the present study also suggests no clear effects of an HPD on CRP, IL-6, and TNF- α, which is in agreement with the general findings that the responses of inflammatory markers are independent of dietary protein intake.^59^^,^^60^ It has been suggested that dietary fat intake, in particular the type of fatty acids consumed (such as docosahexaenoic and eicosapentaenoic acids), is more responsible on changes in these inflammation-related cardiometabolic risk factors than is dietary protein intake.^28^

It is important to consider the influence of other eliminated macronutrients in diets when studying the HPD as variations in the composition of other macronutrients in diets with similar protein content have yielded divergent results,^17^ although this aspect has been overlooked in prior studies. Thus, this study further evaluated the impacts of different macronutrient intake on CVD risk factors using an NMA approach and, overall, the SUCRA results revealed that the HHL is the most recommended diet to ensure a maximum decrease in SBP, DBP, TC, and LDL-C values. In contrast, the LHH generated the lowest scores on the effects of blood pressure lowering (SBP and DBP) and lipid-lipoprotein profile improvement (TC, TG, and LDL-C) and this is possibly due to an increase in the TC and TG levels in the blood circulation after high-fat diet intake. The prolonged exposure to high TC and TG levels can further impair endothelium function by increasing oxidative stress and inflammation, thereby increasing blood pressure.^61^^,^^62^ In addition, compared with the LHH, the HMH and HHL diets reduced SBP by 2.68 mmHg and 3.99 mmHg, respectively. According to a previous meta-analysis, a decrease of approximately 2 mmHg in SBP will be accompanied by a 10% and 7% lower risk of death related to stroke or ischemic heart disease, respectively.^63^ Collectively, these findings suggest that, in addition to higher protein intake, manipulating other macronutrients in the diet, especially dietary fats, is a crucial factor in enhancing the effectiveness of an HPD for reducing the risk of CVD.

With regard to the protein type, results from this study showed that the blood lipid-lipoprotein profile could be improved by a PPR when compared with an APR, which aligns with previous studies,^6^^,^^64^ while no favorable blood pressure improvement effect was observed. The possible explanation relates to the different amino acid profiles displayed in plant- and animal-based proteins. In general, plant-based proteins are lower in essential amino acids but provide greater amounts of the nonessential amino acids. The essential amino acids, such as leucine, lysine, and methionine, have been shown to increase blood cholesterol levels, whereas the nonessential amino acids, such as arginine and glycine, have been found to have the opposite effect.^65–70^ While the exact mechanism is complex and not fully understood, 1 proposed explanation for these effects is that these essential amino acids decrease the clearance of blood LDL-C by reducing the expression of LDL receptors, resulting in hypercholesterolemia. This is supported by 1 prior article, which stated that high-protein diets, particularly those rich in leucine, promote atherosclerosis and plaque instability and stimulate macrophage mammalian target of rapamycin signaling.^66^ However, by upregulating the expression of LDL receptors, arginine and glycine could reduce cholesterol levels in the circulation.^69^^,^^71^^,^^72^ An alternative explanation relates to the sources of protein-rich foods. Plant-based protein serves as a carrier for other well-known antiatherogenic agents, such as plant sterols or soluble fiber. For instance, the soluble fiber contained in a PPR interacts with bile acids and cholesterol in the digestive tract, promoting their excretion and potentially leading to increased HDL-C levels.^73^ Similarly, the animal-based protein–source foods could potentially carry hypercholesterolemic compounds, such as saturated fat and cholesterol.^74–77^ Nevertheless, both animal-based and plant-based proteins have been found to contain bioactive peptides with ACE-inhibitory activity, which elucidates the similar blood pressure reduction effects observed in this study.^48–50^ Also, as described above, the improvements in inflammatory biomarkers achieved by a PPR were limited, primarily attributed to the enhancements in inflammatory markers that are predominantly influenced by alterations in dietary fat intake rather than dietary protein intake.^28^

An important strength of this study is the application of the combined approach of standard and network meta-analysis in assessing the effect of dietary protein quantity. The NMA used in this study dealt with the limitation raised in another meta-analysis article that was published by Vogtschmidt et al^5^—namely, the difficulty in determining whether the effects of an HPD are due to an increase in dietary protein intake or a decrease in intake of other macronutrients. Another strength is that this meta-analysis is based on RCTs of individuals with different health conditions, representing real-life situations. Lastly, there was strong statistical power for the pooled effects due to the substantial number of included studies in standard meta-analysis, and the results obtained from the quantitative analysis were robust and largely stable to sensitivity analysis.

However, several limitations should be considered when interpreting the results. First, considering the variability in participant characteristics and interventions, there could have been a masking effect on the pooled estimates. Even though the sensitivity analysis was conducted to uncover potential differences, an overall high heterogeneity was still discernible and not fully explained by sensitivity analysis. Hence, caution is warranted when generalizing these findings to broader populations, as individual variations may influence the outcomes differently across studies. This study also included articles that used both protein supplements or protein-rich foods as interventions, which can result in the obscuring of true effect sizes. This is due to the varying bioavailability and nutrient complexity of protein supplements and protein-rich foods, which could contribute to different physiological responses when consumed.^78^ Additionally, the NMA applied in this study was only an extension of the standard meta-analysis, which means that, based on the search strategy in this study, it is likely that some studies conforming to the investigation of the impact of dietary macronutrient composition on CVD risk factors were missed.

## CONCLUSION

In conclusion, this study provides insights into the impacts of HPDs and PPRs on cardiovascular health, highlighting a benefit of HPDs and PPRs on vascular health and blood lipid-lipoprotein profile improvement, respectively. Moreover, to maximize the effectiveness of an HPD in improving CVD risk factors, the composition of other macronutrients should also be carefully considered in the dietary approach. However, there is still a need for careful consideration before extrapolating findings to diverse populations for practical implementation in real-life situations. Further large, high-quality RCTs investigating the impacts of macronutrient composition in different diets on CVD risk factors would be useful to help better understand the role of different macronutrients in CVD risk reduction.

## Supplementary Material

nuae086_Supplementary_Data

## Data Availability

Data described in the manuscript will be made available upon request. The request for data should be accompanied by a brief reason and a proposal describing the relevant scientific questions. Requests should be submitted to J.E.K. (fstkje@nus.edu.sg).
