# Supplementary material for: Impact of Quantity and Type of Dietary Protein on Cardiovascular Disease Risk Factors Using Standard and Network Meta-analyses of Randomized Controlled Trials
Source: Nutr Rev. 2024 Jul 16;83(3):e814–28. doi: 10.1093/nutrit/nuae086 (PMC11819482; doi:10.1093/nutrit/nuae086)
Supplement: nuae086_Supplementary_Data [file nuae086_supplementary_data.zip › nuae086_Supplementary_Data/NR_Supplement Tables & Figures.docx]

**Supplementary data**

# **Supplementary Table 1.** Search terms and results for the systematic review assessing the effect of protein on cardiovascular disease risk factors

| **Database** | **Search Terms** | **Filter** | **Results Yielded** |
| --- | --- | --- | --- |
| PubMed | (protein[Title/Abstract] OR proteins[Title/Abstract]) AND ("animal proteins, dietary"[MeSH Terms] OR "meat"[Title/Abstract] OR "meats"[Title/Abstract] OR "poultry"[Title/Abstract] OR "fish"[Title/Abstract] OR "fishes"[Title/Abstract] OR "egg"[Title/Abstract] OR "eggs"[Title/Abstract] OR "milk"[Title/Abstract] OR "dairy"[Title/Abstract] OR "whey"[Title/Abstract] OR "casein"[Title/Abstract]) OR ("plant proteins, dietary"[MeSH Terms] OR plant[Title/Abstract] OR plants[Title/Abstract] OR legume[Title/Abstract] OR legumes[Title/Abstract] OR soy[Title/Abstract] OR soya[Title/Abstract] OR soybean[Title/Abstract] OR pea[Title/Abstract] OR peas[Title/Abstract] OR bean[Title/Abstract] OR beans[Title/Abstract]) AND (lipoprotein hdl2[MeSH Terms] OR lipoproteins, ldl[MeSH Terms] OR cholesterol, ldl[MeSH Terms] OR "blood pressure"[MeSH Terms] OR "triglycerides"[MeSH Terms] OR "flow-mediated dilation") | English  Adult (19+)  Humans | 1,604 |
| Embase | ('protein intake'/exp OR protein:ab,ti OR proteins:ab,ti) AND ('animal protein'/exp OR 'egg protein'/exp OR 'milk protein'/exp OR meat:ab,ti OR meats:ab,ti OR poultry:ab,ti OR fish:ab,ti OR fishes:ab,ti OR egg:ab,ti OR eggs:ab,ti OR dairy:ab,ti OR milk:ab,ti OR whey:ab,ti OR casein:ab,ti OR 'plant protein'/exp OR plant:ab,ti OR plants:ab,ti OR soy:ab,ti OR legume:ab,ti OR legumes:ab,ti OR pea:ab,ti OR peas:ab,ti OR bean:ab,ti OR beans:ab,ti) AND ('cardiovascular disease'/exp OR 'blood pressure':ab,ti OR cholesterol:ab,ti OR lipoprotein:ab,ti OR triglycerides:ab,ti OR 'flow-mediated dilation test':ab,ti) | Embase (Not Medline)  Articles  Adult (18-65 & 65+) | 379 |
| CINAHL | ((MH "Dietary Proteins+" OR AB (meat OR poultry OR fish OR dairy OR milk OR casein OR whey OR "plant protein" OR soy OR bean OR legume OR pea)) AND (MH "Cardiovascular Diseases+" OR MH "Blood Pressure+" OR MH "Cholesterol+" OR MH "Lipoproteins, LDL Cholesterol" OR MH "Lipoproteins, HDL Cholesterol" OR MH "Triglycerides" OR "flow mediated dilation") | English  All adults  Excluding Medline | 595 |
| Web of Science | (((TS=(dietary protein)) AND TS=(cardiovascular disease OR blood pressure OR cholesterol OR lipoproteins OR triglycerides OR flow mediated dilation)) AND AB=(animal OR poultry OR dairy OR milk OR casein OR whey OR soy OR soya OR soybean OR legume OR pea OR beans OR "plant protein")) AND AB=(human) | English, Articles | 698 |
| Cochrane | ([mh Dietary Protein] OR ((meat OR poultry OR fish OR dairy OR whey OR casein OR "plant protein" OR legume OR soy OR bean OR pea):ti,ab,kw)) AND ([mh Cardiovascular disease] OR [mh blood pressure] OR [mh triglycerides] OR [mh cholesterol] OR [mh lipoproteins] OR ((flow mediated dilation):ti,ab,kw)) AND (human OR adult):ti,ab,kw | Trials | 1,557 |
|  | **Total** |  | 4,833 |

**Supplementary data**

# **Supplementary Table 2**. Characteristics of the selected quantity-related studies

| **First author-year (ref)** | **Design** | **Duration (Washout period) (weeks)** | **Group** | **% Energy as Protein** | **Health status** | **n** | **Male %** | **Mean age (year)** | **Mean baseline BMI (kg/m^2^)** | **Protein source** | **Dietary pattern** | **Country** |
| --- | --- | --- | --- | --- | --- | --- | --- | --- | --- | --- | --- | --- |
| Abete-2009 (1) | P | 8 (WO: -) | Control | 19% | Obese | 10 | 100% | 38 | 31.4 | W | ER | Spain |
|  |  | 8 (WO: -) | High Protein | 30% |  | 9 | 100% | 38 | 33.2 | W | ER | Spain |
| Aldrich-2011 (2) | P | 20 (WO: -) | Control | 15% | Healthy | 5 | 17% | 51 | 29.9 | W | ER | USA |
|  |  | 20 (WO: -) | Mixed Protein | 30% |  | 5 | 17% | 50 | 30.3 | S | ER | USA |
|  |  | 20 (WO: -) | Whey Protein | 30% |  | 5 | 17% | 49 | 30.6 | S | ER | USA |
| Azadbakht-2013 (3) | P | 12 (WO: -) | Control | 15% | Overweight, obese | 30 | 0% | 40 | 26.8 | W | ER | Iran |
|  |  | 12 (WO: -) | High Protein | 25% |  | 30 | 0% | 44 | 27.2 | W | ER | Iran |
| Bray-2020 (4) | P | 8 (WO: -) | Normal | 14% | Healthy | 9 | 67% | 23 | NA | W | Overfed | USA |
|  |  | 8 (WO: -) | High Protein | 25% |  | 6 | 50% | 27 | NA | W | Overfed | USA |
| Brinkworth-2004 (5) | P | 68 (WO: -) | Standard | 15% | Obese with hyperinsulinemia | 22 | 32% | 52 | 33.6 | W | ER + EB | Australia |
|  |  | 68 (WO: -) | High Protein | 30% |  | 21 | 24% | 52 | 34.6 | W | ER + EB | Australia |
| Chiu-2014 (6) | P | 4 (WO: -) | Moderate protein high saturated fat | 20% | Overweight, obese | 29 | 30% | 38 | 33.9 | W | EB | USA |
|  |  | 4 (WO: -) | High protein high saturated fat | 30% |  | 32 | 30% | 38 | 33.9 | S | EB | USA |
|  |  | 4 (WO: -) | Moderate protein low saturated fat | 20% |  | 30 | 30% | 38 | 33.9 | W | EB | USA |
|  |  | 4 (WO: -) | High protein low saturated fat | 30% |  | 36 | 30% | 38 | 33.9 | S | EB | USA |
| Claessens-2009 (7) | P | 12 (WO: -) | High Carb | 15% | Overweight | 16 | 38% | 46 | 32.4 | W | HD | Netherlands |
|  |  | 12 (WO: -) | HP Casein | 35% |  | 14 | 36% | 45 | 32.9 | S | HD | Netherlands |
|  |  | 12 (WO: -) | HP Whey | 35% |  | 18 | 33% | 45 | 33.4 | S | HD | Netherlands |
| Dalle Grave-2013 (8) | P | 52 (WO: -) | High protein diet | 34% | Obese | 32 | 40% | 47 | 45.8 | W | ER | Italy |
|  |  | 52 (WO: -) | High carb diet | 17% |  | 37 | 44% | 47 | 45.4 | W | ER | Italy |
| de Luis-2015 (9) | P | 36 (WO: -) | Standard | 20% | Obese | 53 | 35% | 52 | 35.9 | W | ER | Spain |
|  |  | 36 (WO: -) | High protein | 34% |  | 46 | 35% | 52 | 35.8 | W | ER | Spain |
|  |  | 36 (WO: -) | Standard | 20% |  | 41 | 25% | 53 | 36.1 | W | ER | Spain |
|  |  | 36 (WO: -) | High protein | 34% |  | 53 | 25% | 53 | 36.7 | W | ER | Spain |
| Delbridge-2009 (10) | P | 48 (WO: -) | High carbohydrate | 15% | Overweight, obese | 40 | 50% | 44 | 38.6 | W | EB + ER | Australia |
|  |  | 48 (WO: -) | High protein | 30% |  | 42 | 49% | 44 | 39.3 | W | EB + ER | Australia |
| Farnsworth-2003 (11) | P | 16 (WO: -) | Standard | 16% | Overweight, obese, hyperinsulinemia | 22 | 32% | 50 | 33.8 | W | EB + ER | Australia |
|  |  | 16 (WO: -) | High protein | 27% |  | 22 | 32% | 51 | 34.3 | W | EB + ER | Australia |
| Fekete-2016 (12) | C | 8 (WO: 4) | Control | 15% | Prehypertension, mild hypertension | 38 | NR | 30 - 77 | 27.0 | W | NR | UK |
|  |  |  | Whey | 24% |  | 38 |  | 30 - 77 | 27.0 | S | NR | UK |
|  |  |  | Casein | 24% |  | 38 |  | 30 - 77 | 27.0 | S | NR | UK |
| Fernandes-2018 (13) | P | 12 (WO: -) | Low protein + Exercise | 13% | Pre-conditioned older women | 16 | 0% | 68 | 25.4 | S | HD | Brazil |
|  |  | 12 (WO: -) | High Protein + Exercise | 21% |  | 16 | 0% | 67 | 25.9 | S | HD | Brazil |
| Ferrara-2006 (14) | P | 24 (WO: -) | Normal Protein + Training | 15% | Healthy | 8 | 100% | 26 | 23.7 | W | EB | Italy |
|  |  | 24 (WO: -) | High Protein + Training | 20% |  | 7 | 100% | 26 | 23.4 | W | EB | Italy |
| Gögebakan-2011 (15) | P | 26 (WO: -) | Low protein | 18% | Overweight | 195 | 29% | 42 | 30.2 | W | EB | Netherlands |
|  |  | 26 (WO: -) | High protein | 22% |  | 225 | 37% | 42 | 30.5 | W | EB | Netherlands |
| Gulati-2017 (16) | P | 12 (WO: -) | Control | 13% | Overweight, obese | 50 | 42% | 35 | 30.3 | S | ER | India |
|  |  | 12 (WO: -) | High Protein | 28% |  | 50 | 44% | 40 | 30.4 | S | ER | India |
| He-2011 (17) | C | 8 (WO: 3) | Carbohydrates | 15% | Hypertension | 93 | 58% | 48 | 29.3 | S | EB | USA |
|  |  |  | Milk | 21% |  | 88 | 58% | 47 | 29.5 | S | EB | USA |
|  |  |  | Soy | 21% |  | 92 | 59% | 48 | 29.0 | S | EB | USA |
| Hill-2015 (18) | P | 5 (WO: -) | M-DASH (2/3 plant) - Weight maintenance | 18% | Overweight, MetS | 21 | 43% | 45 | 34.7 | W | EB | Australia |
|  |  | 5 (WO: -) | BOLD (2/3 animal) - Weight maintenance | 18% |  | 20 | 45% | 46 | 34.6 | W | EB | Australia |
|  |  | 5 (WO: -) | BOLD+ (2/3 animal HP) - Weight maintenance | 27% |  | 21 | 48% | 46 | 35.1 | W | EB | Australia |
|  |  | 6 (WO: -) | M-DASH (2/3 plant) - Weight loss | 18% |  | 21 | 43% | 45 | 34.7 | W | ER | Australia |
|  |  | 6 (WO: -) | BOLD (2/3 animal) - Weight loss | 18% |  | 20 | 45% | 46 | 34.6 | W | ER | Australia |
|  |  | 6 (WO: -) | BOLD+ (2/3 animal HP) - Weight loss | 27% |  | 21 | 48% | 46 | 35.1 | W | ER | Australia |
|  |  | 12 (WO: -) | M-DASH (2/3 plant) - Free-living weight loss | 18% |  | 21 | 43% | 45 | 34.7 | W | ER | Australia |
|  |  | 12 (WO: -) | BOLD (2/3 animal) - Free-living weight loss | 18% |  | 20 | 45% | 46 | 34.6 | W | ER | Australia |
|  |  | 12 (WO: -) | BOLD+ (2/3 animal HP) - Free-living weight loss | 27% |  | 21 | 48% | 46 | 35.1 | W | ER | Australia |
| Hodgson-2006 (19) | P | 8 (WO: -) | Control | 19% | Hypertensive | 31 | 58% | 60 | 27.9 | W | HD | Australia |
|  |  | 8 (WO: -) | Protein | 24% |  | 29 | 69% | 57 | 27.5 | W | HD | Australia |
| Hodgson-2012 (20) | P | 104 (WO: -) | Control | 18% | Healthy | 55 | 0% | 74 | 27.2 | W | NR | Australia |
|  |  | 104 (WO: -) | Protein | 23% |  | 49 | 0% | 74 | 26.3 | S | NR | Australia |
| Hudson-2020 (21) | P | 16 (WO: -) | Control | 17% | Overweight, obese | 23 | 26% | 52 | 30.3 | W | ER | USA |
|  |  | 16 (WO: -) | Milk protein isolate | 33% |  | 21 | 24% | 53 | 31.0 | S | ER | USA |
| Jenkins-2000 (22) | C | 3 (WO: 2) | Control | 16% | Hyperlipidaemic | 25 | 60% | 60 | 25.1 | W | NR | Canada |
|  |  |  | Soy | 21% |  | 25 |  | 60 | 25.1 | S | NR | Canada |
| Johnston-2004 (23) | P | 6 (WO: -) | High carbohydrate low fat | 15% | Healthy | 7 | 14% | 36 | 28.7 | W | ER | USA |
|  |  | 6 (WO: -) | High protein low fat | 30% |  | 9 | 11% | 40 | 29.1 | W | ER | USA |
| Juraschek-2013 (24) | C | 6 (WO: 2 - 4) | Unsaturated fat | 15% | Hypertension | 164 | 55% | 54 | 30.2 | W | EB | USA |
|  |  |  | Carbohydrate | 15% |  | 164 |  | 54 | 30.2 | W | EB | USA |
|  |  |  | Protein | 25% |  | 164 |  | 54 | 30.2 | W | EB | USA |
| Machin-2014 (25) | C | 4 (WO: 2) | High dairy | 15% | Prehypertension and hypertension | 49 | 44% | 53 | 30.5 | W | NR | USA |
|  |  |  | No dairy | 21% |  | 49 |  | 53 | 30.4 | W | NR | USA |
| Mamo-2005 (26) | P | 6 (WO: -) | Low protein diet | 14% | Moderately hypertriglyceridemia but otherwise healthy subjects | 10 | 70% | 41 | 31.7 | W | EB | Australia |
|  |  | 6 (WO: -) | High protein diet | 25% |  | 10 | 50% | 55 | 31.7 | W | EB | Australia |
| Mateo-Gallego-2017 (27) | P | 12 (WO: -) | 20% Protein | 24% | Overweight, obese | 24 | 0% | 44 | 33.2 | W | ER | Spain |
|  |  | 12 (WO: -) | 27% Protein | 27% |  | 29 | 0% | 45 | 33.0 | W | ER | Spain |
|  |  | 12 (WO: -) | 35% Protein | 31% |  | 27 | 0% | 43 | 32.4 | W | ER | Spain |
| McAuley-2006 (28) | P | 16 (WO: -) | High carbohydrate diet | 15% | Overweight, insulin-resistant | 30 | 0% | 45 | 36.6 | W | ER + EB | New Zealand |
|  |  | 16 (WO: -) | High protein diet | 30% |  | 27 | 0% | 47 | 34.5 | W | HD | New Zealand |
| Meckling-2007 (29) | P | 12 (WO: -) | Normal protein | 18% | Overweight, obese | 8 | 0% | 47 | 28.7 | W | ER | Canada |
|  |  | 12 (WO: -) | High protein | 35% |  | 10 | 0% | 45 | 31.2 | W | ER | Canada |
|  |  | 12 (WO: -) | Normal protein + Exercise | 18% |  | 11 | 0% | 41 | 29.2 | W | ER | Canada |
|  |  | 12 (WO: -) | High protein + Exercise | 35% |  | 14 | 0% | 37 | 30.8 | W | ER | Canada |
| Mehrabani-2012 (30) | P | 12 (WO: -) | Conventional hypocaloric diet | 15% | Overweight, obese | 26 | 0% | 29 | 31.1 | W | ER | Iran |
|  |  | 12 (WO: -) | Modified hypocaloric diet | 30% |  | 23 | 0% | 31 | 31.9 | W | ER | Iran |
| Noakes-2005 (31) | P | 12 (WO: -) | High-carbohydrate | 18% | Obese | 47 | 0% | 49 | 33.0 | W | ER | Australia |
|  |  | 12 (WO: -) | High-protein | 31% |  | 52 | 0% | 50 | 32.0 | W | ER | Australia |
| Papakonstantinou-2010 (32) | C | 4 (WO: 3) | Low-protein high-fat diet | 15% | Obese with type 2 diabetes mellitus | 17 | 29% | 46 | 34.0 | W | ER | Europe |
|  |  |  | High-protein low-fat diet | 30% |  | 17 |  | 46 | 33.0 | W | ER | Europe |
| Pedersen-2014 (33) | P | 12 (WO: -) | Standard protein diet | 20% | Overweight, obese | 24 | 83% | 62 | 35.4 | W | ER | Australia |
|  |  | 12 (WO: -) | High protein diet | 30% |  | 21 | 71% | 59 | 36.7 | W | ER | Australia |
| Porter-2019 (34) | P | 6 (WO: -) | Normal protein intake | 15% | Obese | 27 | 11% | 66 | 37.2 | W | ER | USA |
|  |  | 6 (WO: -) | High protein intake | 30% |  | 53 | 9% | 63 | 37.3 | W | ER | USA |
| Roussell-2012 (35) | C | 5 (WO: 1) | Lower protein amount (DASH) | 18% | Elevated LDL | 36 | 42% | 50 | 25.7 | W | NR | USA |
|  |  |  | Lower protein amount (BOLD) | 19% |  | 36 |  | 50 | 25.7 | W | NR | USA |
|  |  |  | High proteiin amount (BOLD+) | 27% |  | 36 |  | 50 | 25.7 | W | NR | USA |
| Sacks-1984 (36) | C | 6 (WO: 0) | Normal-protein diet | 16% | Healthy | 18 | 39% | 32 | NA | W | NR | USA |
|  |  |  | High-protein diet | 25% |  | 18 |  | 32 | NA | W | NR | USA |
| Sacks-2009 (37) | P | 96 (WO: -) | Low fat, average protein diet | 15% | Overweight, obese | 204 | 38% | 52 | 33.0 | W | ER | USA |
|  |  | 96 (WO: -) | Low fat, high protein diet | 25% |  | 202 | 33% | 50 | 33.0 | W | ER | USA |
|  |  | 96 (WO: -) | High fat, average protein diet | 15% |  | 204 | 39% | 52 | 32.0 | W | ER | USA |
|  |  | 96 (WO: -) | High fat, high protein diet | 25% |  | 201 | 36% | 51 | 33.0 | W | ER | USA |
| Sheikholeslami-2012 (38) | P | 6 (WO: -) | Training + Placebo | 19% | Overweight | 10 | 100% | 23 | 27.0 | W | HD | Iran |
|  |  | 6 (WO: -) | Training + Whey | 20% |  | 10 | 100% | 23 | 26.8 | S | HD | Iran |
| Stephenson-2005 (39) | C | 8 (WO: 0) | Control | 16% | Type 1 diabetes | 12 | 50% | 30 | 26.6 | W | HD | USA |
|  |  |  | Soy | 22% |  | 12 |  | 30 | 26.6 | W | HD | USA |
| Tang-2013 (40) | P | 12 (WO: -) | Normal protein diet | 15% | Overweight, obese | 21 | 100% | 45 | 32.0 | W | ER | USA |
|  |  | 12 (WO: -) | High protein diet | 25% |  | 22 | 100% | 51 | 31.4 | W | ER | USA |
| Teunisseti-Beekman-2012 (41) | P | 4 (WO: -) | Normal protein intake | 15% | Overweight | 51 | 69% | 55 | 28.8 | W | NR | Netherlands |
|  |  | 4 (WO: -) | Higher protein intake | 25% |  | 43 | 67% | 55 | 27.9 | S | NR | Netherlands |
| Tischmann-2020 (42) | P | 238 (WO: -) | Moderate protein | 15% | Overweight | 18 | 50% | 65 | 29.0 | W | EB | Netherlands |
|  |  | 238 (WO: -) | High protein | 25% |  | 20 | 35% | 64 | 28.9 | W | EB | Netherlands |
| Toscani-2011 (43) | P | 8 (WO: -) | Normal protein | 15% | Polycystic ovary syndrome | 9 | 0% | 23 | NA | W | ER | Brazil |
|  |  | 8 (WO: -) | High protein | 30% |  | 9 | 0% | 23 | NA | W | ER | Brazil |
|  |  | 8 (WO: -) | Normal protein diet | 15% | Healthy | 9 | 0% | 29 | NA | W | ER | Brazil |
|  |  | 8 (WO: -) | High protein diet | 30% |  | 13 | 0% | 29 | NA | W | ER | Brazil |
| Treyzon-2008 (44) | P | 12 (WO: -) | Standard protein diet | 15% | Obese | 42 | 36% | 50 | 32.7 | W | ER | USA |
|  |  | 12 (WO: -) | High protein diet | 30% |  | 45 | 24% | 49 | 33.8 | S | ER | USA |
| Weinheimer-2012 (45) | P | 36 (WO: -) | 0 g + Exercise | 15% | Overweight, obese | 70 | 40% | 49 | 29.9 | W | HD | USA |
|  |  | 36 (WO: -) | 20 g whey + Exercise | 18% |  | 71 | 41% | 47 | 30.4 | S | HD | USA |
|  |  | 36 (WO: -) | 40 g whey + Exercise | 22% |  | 21 | 32% | 46 | 29.4 | S | HD | USA |
|  |  | 36 (WO: -) | 60 g whey + Exercise | 25% |  | 26 | 40% | 50 | 30.7 | S | HD | USA |
| Wolfe-1991 (46) | C | 4 or 5 (WO: 0) | Normal protein | 11% | Moderate hypercholesterolemic | 10 | 40% | 50 | 24.4 | W | NR | Canada |
|  |  |  | High protein | 23% |  | 10 |  | 50 | 24.4 | W | NR | Canada |
| Wolfe-1992 (47) | C | 4 or 5 (WO: 0) | Normal protein | 10% | Familial hypercholesterolemia receiving cholestyramine 18 g/d | 5 | 40% | 48 | 28.4 | W | NR | Canada |
|  |  |  | High protein | 27% |  | 5 |  | 48 | 28.4 | W | NR | Canada |
| Wolfe-1999 (48) | C | 4 (WO: 2) | Normal protein | 12% | Healthy | 10 | 20% | 28 | 23.0 | W | NR | Canada |
|  |  |  | High protein | 22% |  | 10 |  | 28 | 23.0 | W | NR | Canada |
| Yilmaz-2021 (49) | P | 8 (WO: -) | Low protein diet | 15% | Obese | 30 | 0% | 34 | 32.5 | W | NR | Turkey |
|  |  | 8 (WO: -) | High protein diet | 25% |  | 30 | 0% | 32 | 32.5 | W | NR | Turkey |

BMI, Body Mass Index; C, Crossover; EB, Energy balance; ER, Energy restriction; HD, Habitual diet; MetS, Metabolism syndrome; n, Participants number; NR, Not reported; P, Parallel; S, Supplements; W, Whole foods.

**Supplementary data**

# **Supplementary Table 3**. Characteristics of the selected type-related studies

| **First author-year (ref)** | **Design** | **Duration (Washout period) (weeks)** | **Group** | **Health status** | **n** | **Male %** | **Mean age (year)** | **Mean baseline BMI (kg/m^2^)** | **Protein source** | **Dietary pattern** | **Country** |
| --- | --- | --- | --- | --- | --- | --- | --- | --- | --- | --- | --- |
| Anderson-2007 (50) | P | 16 (WO: -) | Casein 91 g + 10 mg isoflavones | Obese | 18 | 0% | 44 | 34.9 | S | ER | USA |
|  |  | 16 (WO: -) | Soy 91 g + 150 mg isoflavones |  | 17 | 0% | 47 | 34.6 | S | ER | USA |
| Ashton-2000 (51) | C | 4 (WO: 2) | Lean Meat 150 g/day | Healthy | 42 | 100% | 35 - 62 | 26.2 | W | HD | Australia |
|  |  |  | Tofu 290 g/day |  | 42 | 100% | 35 - 62 | 26.2 | W | HD | Australia |
| Azadbakht-2003 (52) | C | 7 (WO: 4) | Animal (70% Animal protein; 30% Plant protein) | T2DM with nephropathy | 14 | 71% | 63 | NR | W | HD | Iran |
|  |  |  | Soy (35% Animal protein; 65% Plant protein) |  | 14 | 71% | 63 | NR | W | HD | Iran |
| Azadbakht-2008 (53) | P | 208 (WO: -) | Animal (70% Animal protein; 30% Plant protein) | T2DM with nephropathy | 21 | 44% | 62 | NR | W | EB | Iran |
|  |  | 208 (WO: -) | Soy (35% Animal protein; 65% Plant protein) |  | 20 | 44% | 62 | NR | W | EB | Iran |
| Azadbakht-2011 (54) | C | 6 (WO: 3) | Diet with Cow's milk | Overweight or Obese | 23 | 0% | 22 | 28.1 | W | ER | Iran |
|  |  |  | Diet with Soy milk |  | 23 | 0% | 22 | 28.1 | W | ER | Iran |
| Bahr-2013 (55) | C | 8 (WO: 4) | Milk protein isolate (MPI) 25 g first then LPI | Hypercholesterolemic | 33 | 29% | 49 | 28.0 | S | HD | Germany |
|  |  |  | Lupin protein isolate (LPI) 25 g first then MPI |  | 33 | 63% | 50 | 28.0 | S | HD | Germany |
| Bahr-2015 (56) | C | 4 (WO: 6) | Milk protein isolate 25 g | Hypercholesterolemic | 22 | 32% | 57 | 26.4 | S | HD | Germany |
|  |  |  | Lupin protein isolate 25 g |  | 22 | 50% | 54 | 26.4 | S | HD | Germany |
| Bakhit-1994 (57) | C | 4 (WO: 0) | Casein + Cellulose | Hypercholesterolemic | 21 | 100% | 43 | 27.0 | S | EB | USA |
|  |  |  | Soy + Cellulose |  | 21 | 100% | 43 | 27.0 | S | EB | USA |
|  | C |  | Casein + Soybean cotyledon fibre |  | 21 | 100% | 43 | 27.0 | S | EB | USA |
|  |  |  | Soy + Soybean cotyledon fibre |  | 21 | 100% | 43 | 27.0 | S | EB | USA |
| Basciani-2020 (58) | P | 6 (WO: -) | Whey 90 g | Obesity | 16 | 40% | 56 | 35.8 | S | ER | Italy |
|  |  | 6 (WO: -) | Plant 90 g |  | 16 | 40% |  | 36.1 | S | ER | Italy |
|  |  | 6 (WO: -) | Animal 90 g |  | 16 | 40% |  | 35.7 | W | ER | Italy |
| Beavers-2010 (59) | P | 4 (WO: -) | Milk 24 g protein | Healthy | 16 | 0% | 55 | 26.3 | W | EB | USA |
|  |  | 4 (WO: -) | Soy milk 18 protein |  | 16 | 0% | 54 | 25.4 | W | EB | USA |
| Bergeron-2019 (60) | P | 4 (WO: 2 to 7) | High SFA Red meat | Healthy | 61 | 44% | 45 | 25.9 | W | EB | USA |
|  |  | 4 (WO: 2 to 7) | High SFA White meat |  | 61 | 44% | 45 | 25.9 | W | EB | USA |
|  |  | 4 (WO: 2 to 7) | High SFA Nonmeat |  | 61 | 44% | 45 | 25.9 | W | EB | USA |
|  |  | 4 (WO: 2 to 7) | Low SFA Red Meat |  | 52 | 33% | 42 | 26.0 | W | EB | USA |
|  |  | 4 (WO: 2 to 7) | Low SFA White Meat |  | 52 | 33% | 42 | 26.0 | W | EB | USA |
|  |  | 4 (WO: 2 to 7) | Low SFA Nonmeat |  | 52 | 33% | 42 | 26.0 | W | EB | USA |
| Borodin-2009 (61) | C | 8 (WO: 4) | Soy 30 g protein | Hyperlipidaemia | 28 | 32% | 51 | 28.7 | S | HD | Japan |
|  |  |  | Skimmed milk 30 g protein |  | 28 | 32% | 51 | 29.2 | S | HD | Japan |
| Bosello-1988 (62) | P | 8 (WO: -) | Casein 28 g protein | Obese | 12 | 50% | 25 - 42 | NR | S | ER | Italy |
|  |  | 8 (WO: -) | Soy 28 g protein |  | 12 | 50% | 25 - 42 | NR | S | ER | Italy |
| Bricarello-2004 (63) | C | 6 (WO: 0) | Cow's milk 27.5 g protein | Hypercholesterolemia | 60 | 25% | 56 | 24.9 | W | NR | Brazil |
|  |  |  | Soy 25 g protein |  | 60 | 25% | 56 | 24.9 | W | NR | Brazil |
| Campbell-2010 (64) | P | 48 (WO: -) | Casein 25 g protein | Healthy | 27 | 0% | 56 | 27.3 | S | NR | USA |
|  |  | 48 (WO: -) | Soy 25 g protein |  | 35 | 0% | 53 | 28.6 | S | NR | USA |
| Chen-2005 (65) | P | 12 (WO: -) | Milk protein isolate 30 g | Hyperlipidaemic | 9 | 22% | 66 | 24.4 | S | HD | China |
|  |  | 12 (WO: -) | Soy protein isolate 30 g |  | 10 | 30% | 61 | 23.6 | S | HD | China |
|  |  | 12 (WO: -) | Milk protein isolate 30 g | Healthy | 10 | 30% | 60 | 21.2 | S | HD | China |
|  |  | 12 (WO: -) | Soy protein isolate 30 g |  | 8 | 25% | 59 | 21.5 | S | HD | China |
| Chen-2006 (66) | P | 12 (WO: -) | Milk protein 30 g | Non-diabetic hypercholesterolaemia | 13 | 69% | 58 | 22.6 | S | HD | China |
|  |  | 12 (WO: -) | Soy protein 30 g |  | 13 | 77% | 59 | 23.5 | S | HD | China |
| Crimarco-2020 (67) | C | 8 (WO: 0) | Animal (67% Animal protein; 33% Plant protein) | Healthy | 36 | 33% | 50 | 27.9 | W | HD | USA |
|  |  |  | Plant (27% Animal protein; 73% Plant protein) |  | 36 | 33% | 50 | 27.9 | W | HD | USA |
| Cuevas-2003 (68) | C | 8 (WO: 0) | Caseinate 40 g | Healthy | 18 | 0% | 59 | 29.3 | S | NR | Chile |
|  |  |  | Soy protein 40 g |  | 18 | 0% | 59 | 29.3 | S | NR | Chile |
| Desroches-2004 (69) | C | 6 (WO: 2-4) | Animal protein (contributes 17% total energy) | Hypercholesterolemic | 36 | 44% | 63 | 27.6 | W | EB | Canada |
|  |  |  | Soy/- isoflavones (contributes 17% total energy) |  | 36 | 44% | 63 | 27.6 | S | EB | Canada |
|  | C |  | Animal/+ 52 mg aglycone isoflavones/1000 kcal (contributes 15% total energy) |  | 36 | 44% | 63 | 27.6 | W | EB | Canada |
|  |  |  | Soy/+ 46 mg aglycone isoflavones/1000 kcal (contributes 16% total energy) |  | 36 | 44% | 63 | 27.6 | S | EB | Canada |
| Frota-2015 (70) | C | 6 (WO: 4) | Casein 25 g | Hypercholesterolemic | 38 | 27% | 57 | 27.1 | S | HD | Brazil |
|  |  |  | Cowpea protein isolate 25 g |  | 38 | 27% | 57 | 27.3 | S | HD | Brazil |
| Gardner-2001 (71) | P | 12 (WO: -) | Milk protein 42 g | Hypercholesterolemic | 30 | 0% | 58 | 27.1 | S | ER | USA |
|  |  | 12 (WO: -) | Soy protein 42 g |  | 33 | 0% | 58 | 25.4 | S | ER | USA |
|  |  | 12 (WO: -) | Soy protein 42 g + isoflavones 80 mg |  | 31 | 0% | 63 | 25.6 | S | ER | USA |
| Gardner-2007 (72) | C | 4 (WO: 4) | Dairy 25 g protein | Hypercholesterolemic | 28 | 21% | 52 | 26.0 | W | NR | USA |
|  |  |  | Soy protein isolate 25 g |  | 28 | 21% | 52 | 26.0 | S | NR | USA |
|  |  |  | Whole bean 25 g protein |  | 28 | 21% | 52 | 26.0 | W | NR | USA |
| George-2020 (73) | P | 12 (WO: -) | Casein 40 g | Not specific | 44 | 50% | 61 | 30.6 | S | NR | USA |
|  |  | 12 (WO: -) | Soy protein 40 g + 96 mg isoflavones |  | 44 | 55% | 60 | 31.6 | S | NR | USA |
| He-2011 (17) | C | 8 (WO: 3) | Milk 40 g protein | Hypertension | 88 | 58% | 47 | 29.5 | S | EB | USA |
|  |  |  | Soy 40 g protein |  | 92 | 59% | 48 | 29.0 | S | EB | USA |
| Hill-2015 (18) | P | 5 (WO: -) | BOLD (2/3 animal) - Weight maintenance 103 g protein/day | Overweight, MetS | 20 | 45% | 46 | 34.6 | W | EB | Australia |
|  |  | 5 (WO: -) | BOLD+ (2/3 animal HP) - Weight maintenance 150 g protein/day |  | 21 | 48% | 46 | 35.1 | W | EB | Australia |
|  |  | 5 (WO: -) | M-DASH (2/3 plant) - Weight maintenance 100 g protein/day |  | 21 | 43% | 45 | 34.7 | W | EB | Australia |
|  |  | 6 (WO: -) | BOLD (2/3 animal) - Weight loss 103 g protein/day |  | 20 | 45% | 46 | 34.6 | W | ER | Australia |
|  |  | 6 (WO: -) | BOLD+ (2/3 animal HP) - Weight loss 150 g protein/day |  | 21 | 48% | 46 | 35.1 | W | ER | Australia |
|  |  | 6 (WO: -) | M-DASH (2/3 plant) - Weight loss 100 g protein/day |  | 21 | 43% | 45 | 34.7 | W | ER | Australia |
|  |  | 12 (WO: -) | BOLD (2/3 animal) - Free living weight-loss 103 g protein/day |  | 20 | 45% | 46 | 34.6 | W | ER | Australia |
|  |  | 12 (WO: -) | BOLD+ (2/3 animal HP) - Free living weight-loss 150 g protein/day |  | 21 | 48% | 46 | 35.1 | W | ER | Australia |
|  |  | 12 (WO: -) | M-DASH (2/3 plant) - Free living weight-loss 100 g protein/day |  | 21 | 43% | 45 | 34.7 | W | ER | Australia |
| Jamilian-2015 (74) | P | 6 (WO: -) | Control (70% Animal protein; 30% Plant protein) | Gestational diabetes mellitus | 32 | 0% | 29 | 28.4 | W | HD | Iran |
|  |  | 6 (WO: -) | Soy (35% Animal protein; 65% Plant protein) |  | 34 | 0% | 28 | 28.9 | W | HD | Iran |
| Jassi-2010 (75) | P | 12 (WO: -) | Soy protein 30 g + 60 mg isoflavones | Healthy | 25 | 0% | 51 | 23.3 | S | NR | India |
|  |  | 12 (WO: -) | Casein 30 g |  | 25 | 0% | 51 | 23.6 | S | NR | India |
| Jenkins-2000 (76) | C | 4 (WO: 2) | Control (Animal protein: Plant protein= 94 g: 27 g; Total protein: 121 g) | Hyperlipidaemic | 31 | 61% | 57 | NR | W | NR | Canada |
|  |  |  | Test (Animal protein: Plant protein= 8 g: 110 g; Total protein: 118 g) |  | 31 | 61% | 57 | NR | W | NR | Canada |
| Karamali-2018 (77) | P | 8 (WO: -) | Animal (70% Animal protein; 3u0% Plant protein) | Diabetes, all on metformin | 30 | 0% | 26 | 27.8 | W | EB | Iran |
|  |  | 8 (WO: -) | Plant (35% Animal protein; 65% Plant protein) |  | 30 | 0% | 25 | 28.7 | W | EB | Iran |
| Kurowska-1997 (78) | C | 4 (WO: 2) | Milk protein 98 g | Mild hypercholesterolemia | 34 | 50% | 55 | NR | W | HD | Canada |
|  |  |  | Soybean protein 96 g |  | 34 | 50% | 55 | NR | W | HD | Canada |
| Liao-2007 (79) | P | 8 (WO: -) | Traditional (67% Animal protein; 33% Plant protein) | Overweight | 15 | 20% | 38 | 30.0 | W | ER | China |
|  |  | 8 (WO: -) | Soy (100% Plant protein) |  | 15 | 20% | 29 | 29.6 | W | ER | China |
| Liu-2012 (80) | P | 24 (WO: -) | Milk protein 15 g + 100 mg isoflavones | Healthy | 57 | 0% | 56 | 24.7 | S | HD | China |
|  |  | 24 (WO: -) | Milk protein 15 g |  | 50 | 0% | 56 | 24.6 | S | HD | China |
|  |  | 24 (WO: -) | Soy protein 15 g + 100 mg isoflavones |  | 57 | 0% | 56 | 24.1 | S | HD | China |
| Liu-2013 (81) | P | 24 (WO: -) | Milk protein 15 g | Normotensive | 16 | 0% | 55 | 22.6 | S | HD | China |
|  |  | 24 (WO: -) | Soy protein 15 g |  | 17 | 0% | 54 | 23.6 | S | HD | China |
|  |  | 24 (WO: -) | Soy protein 15 g + 100 mg isoflavones |  | 17 | 0% | 56 | 23.9 | S | HD | China |
|  |  | 24 (WO: -) | Milk protein 15 g | Pre and hypertensive | 44 | 0% | 56 | 25.3 | S | HD | China |
|  |  | 24 (WO: -) | Soy protein 15 g |  | 43 | 0% | 57 | 24.3 | S | HD | China |
|  |  | 24 (WO: -) | Soy protein 15 g + 100 mg isoflavones |  | 43 | 0% | 56 | 25.1 | S | HD | China |
| Liu-2014 (82) | P | 24 (WO: -) | Low fat milk 12.7 g protein | Equol-producing (24-h urinary log10 S-equol/daidzein ratio greater than −1.75 after daidzein challenge) | 81 | 0% | 59 | 23.7 | S | NR | China |
|  |  | 24 (WO: -) | Low Fat Milk + 63 mg Daidzein 12.7 g protein |  | 87 | 0% | 58 | 23.2 | S | NR | China |
|  |  | 24 (WO: -) | Whole soy (49.8 mg isoflavones) 12.8 g protein |  | 85 | 0% | 58 | 23.3 | S | NR | China |
| Ma-2011 (83) | P | 8 (WO: -) | Milk protein 24 g | Moderately hypercholesterolemic | 45 | 27% | 52 | 23.3 | S | HD | China |
|  |  | 8 (WO: -) | Soy 18 g; Milk 6 g |  | 45 | 31% | 51 | 23.8 | S | HD | China |
| Maki-2010 (84) | P | 4 (WO: -) | Total milk protein 25 g | Hypercholesterolemic | 28 | 46% | 52 | 27.1 | S | NR | USA |
|  |  | 4 (WO: -) | Soy protein 25 g |  | 30 | 43% | 50 | 28.3 | S | NR | USA |
| Matthan-2007 (85) | C | 6 (WO: 2 to 4) | Animal protein (Animal: 75 g) | Hypercholesterolemic | 28 | 7% | 65 | 27.0 | W | EB | USA |
|  |  |  | Soybean protein diet (Soy 37.5 g + Animal: 37.5 g) |  | 28 | 7% | 65 | 27.0 | W | EB | USA |
|  |  |  | Soy flour (Soy 37.5 g + Animal: 37.5 g) |  | 28 | 7% | 65 | 27.0 | W | EB | USA |
|  |  |  | Soymilk (Soy 37.5 g + Animal: 37.5 g) |  | 28 | 7% | 65 | 27.0 | W | EB | USA |
| McVeigh-2006 (86) | C | 8 (WO: 4) | Milk protein isolate 32 g | Healthy | 35 | 100% | 28 | 25.4 | S | HD | USA |
|  |  |  | Soy protein isolate 32 g + low-isoflavones |  | 35 | 100% | 28 | 25.4 | S | HD | USA |
|  |  |  | Soy protein isolate 32 g + high-isoflavones |  | 35 | 100% | 28 | 25.4 | S | HD | USA |
| Meinertz-1988 (87) | C | 4 (WO: 4) | Low-cholesterol (less than 100 mg/d) Casein 139 g | Healthy | 10 | 50% | 41 | NR | S | NR | Denmark |
|  |  |  | Low-cholesterol (less than 100 mg/d) Soy 140 g |  | 10 | 50% | 41 | NR | S | NR | Denmark |
| Meinertz-1989 (88) | C | 4 (WO: 4) | Cholesterol-enriched (500 mg/d) Casein 139 g | Healthy | 11 | 45% | 38 | NR | S | NR | Denmark |
|  |  |  | Cholesterol-enriched (500 mg/d) Soy 140 g |  | 11 | 45% | 38 | NR | S | NR | Denmark |
| Nilausen-1998 (89) | C | 4 (WO: 7) | Casein 154 g | Healthy | 9 | 100% | 37 | 22.9 | S | NR | Europe |
|  |  |  | Soy 154 g |  | 9 | 100% | 37 | 22.9 | S | NR | Europe |
| Padhi-2015 (90) | P | 6 (WO: -) | Whey protein 25 g | Elevated LDL cholesterol and not taking cholesterol-lowering medications | 73 | 37% | 54 | 28.1 | S | HD | Canada |
|  |  | 6 (WO: -) | Soy protein 25 g |  | 72 | 37% | 56 | 27.7 | S | HD | Canada |
| Pipe-2009 (91) | C | 8 (WO: 4) | Milk protein isolate 40 g | Type 2 Diabetes | 29 | 55% | 60 | 29.6 | S | HD | Canada |
|  |  |  | Soy proten isolate 40 g + 80 mg isoflavones |  | 29 | 55% | 60 | 29.6 | S | HD | Canada |
| Prescott-1987 (92) | P | 12 (WO: -) | Animal protein 93 g | Healthy | 25 | NR | 18 - 60 | NR | S | NR | Australia |
|  |  | 12 (WO: -) | Plant protein 84 g |  | 25 | NR | 18 - 60 | NR | S | NR | Australia |
| Rivas-2002 (93) | P | 12 (WO: -) | Cow's milk 15.5 g | Hypertension | 20 | 55% | 49 | NR | W | HD | Europe |
|  |  | 12 (WO: -) | Soy 18 g + 143 mg isoflavones |  | 20 | 70% | 48 | NR | W | HD | Europe |
| Santo-2010 (94) | P | 4 (WO: -) | Milk protein isolate 25 g | Healthy | 9 | 100% | 24 | 23.4 | S | HD | USA |
|  |  | 4 (WO: -) | (Soy-) isoflavone-poor soy protein isolate 25 g |  | 11 | 100% | 24 | 25.2 | S | HD | USA |
|  |  | 4 (WO: -) | (Soy+) isoflavone-rich soy protein isolate 25 g |  | 10 | 100% | 25 | 22.8 | S | HD | USA |
| Shidfar-2009 (95) | P | 10 (WO: -) | Whey protein 50 g | Hypercholesterolemic | 21 | 0% | 57 | 26.8 | S | HD | Iran |
|  |  | 10 (WO: -) | Soy protein 50 g + 164 mg isoflavones |  | 21 | 0% | 53 | 27.1 | W | HD | Iran |
| Shige-1998 (96) | C | 3 (WO: 2) | Casein 20 g | Healthy | 11 | 100% | 33 | 24.6 | S | NR | Japan |
|  |  |  | Soy protein isolate 20 g |  | 11 | 100% | 33 | 24.6 | S | NR | Japan |
| Sirtori-2012 (97) | P | 4 (WO: -) | Casein 40.6 g + cellulose | Moderately hypercholesterolaemia | 25 | 40% | 55 | 25.4 | S | NR | Europe |
|  |  | 4 (WO: -) | Lupin protein 34.6 g + cellulose |  | 22 | 52% | 52 | 24.0 | S | NR | Europe |
|  |  | 4 (WO: -) | Pea protein 34.6 g + cellulose |  | 25 | 56% | 53 | 25.0 | S | NR | Europe |
| Sucher-2017 (98) | P | 6 (WO: -) | Animal (Animal protein: Plant protein= 80: 20) | Type 2 Diabetes | 18 | 67% | 65 | 31.0 | W | EB | Europe |
|  |  | 6 (WO: -) | Plant (Animal protein: Plant protein= 72: 28) |  | 19 | 63% | 64 | 29.4 | W | EB | Europe |
| Tabibi-2010 (99) | P | 8 (WO: -) | Control (Animal protein: Plant protein= 22 g: 18 g) | Peritoneal dialysis patients | 18 | 50% | 53 | 27.0 | W | HD | Iran |
|  |  | 8 (WO: -) | Soy (Animal protein: Plant protein= 14 g: 29 g) + 61 mg isoflavones |  | 18 | 50% | 52 | 25.0 | W | HD | Iran |
| Tahavorgar-2015 (100) | P | 12 (WO: -) | Whey protein preloads 54 g | Overweight or obese | 26 | 100% | 39 | 32.1 | S | HD | Iran |
|  |  | 12 (WO: -) | Soy protein preloads 54 g |  | 19 | 100% | 39 | 32.1 | S | HD | Iran |
| Teede-2001 (101) | P | 12 (WO: -) | Casein 40 g | Healthy | 93 | 49% | 60 | 26.0 | S | HD | Australia |
|  |  | 12 (WO: -) | Soy protein isolate 40 g + 118 mg isoflavones |  | 86 | 54% | 61 | 25.0 | S | HD | Australia |
| Teixeira-2000 (102) | P | 6 (WO: -) | Soy protein isolate 0 g + Casein 50 g | Moderately hypercholesterolemic | 16 | 100% | 50 | 27.1 | S | NR | USA |
|  |  | 6 (WO: -) | Soy protein isolate 50 g + Casein 0 g |  | 15 | 100% | 45 | 27.4 | S | NR | USA |
|  |  | 6 (WO: -) | Soy protein isolate 20 g + Casein 30 g |  | 15 | 100% | 42 | 27.8 | S | NR | USA |
|  |  | 6 (WO: -) | Soy protein isolate 30 g + Casein 20 g |  | 18 | 100% | 43 | 27.1 | S | NR | USA |
| Tonstad-2002 (103) | P | 16 (WO: -) | Casein 50 g | Healthy | 29 | 84% | 52 | 25.2 | W | NR | Europe |
|  |  | 16 (WO: -) | Soy protein isolate 50 g |  | 31 | 90% | 54 | 25.4 | W | NR | Europe |
|  |  | 16 (WO: -) | Casein 30 g |  | 36 | 81% | 52 | 25.2 | W | NR | Europe |
|  |  | 238 (WO: -) | Soy protein isolate 30 g |  | 34 | 79% | 51 | 25.4 | W | NR | Europe |
| van Raaij-1981 (104) | P | 4 (WO: -) | Casein (contributes to 8.8% energy) | Healthy | 23 | 61% | 18 - 28 | NR | W | EB | Netherlands |
|  |  | 4 (WO: -) | Soy (contributes to 8.4% energy) |  | 23 |  | 18 - 28 | NR | W | EB | Netherlands |
| van Raaij-1982 (105) | P | 4 (WO: -) | Casein 9.6 g | Healthy | 17 | 52% | 46 | NR | W | EB | Netherlands |
|  |  | 4 (WO: -) | Soy isolates 9.8 g |  | 20 |  | 46 | NR | W | EB | Netherlands |
|  |  | 4 (WO: -) | Soy concentrates 9.8 g |  | 20 |  | 46 | NR | W | EB | Netherlands |
| Vigna-2000 (106) | P | 12 (WO: -) | Casein 40 g | Healthy | 37 | 0% | 53 | 25.9 | S | NR | Europe |
|  |  | 12 (WO: -) | Soy protein 40 g + 76 mg isoflavones |  | 40 | 0% | 54 | 25.9 | S | NR | Europe |
| Wang-1995 (107) | C | 2 (WO: 0) | Casein (contributes 8% energy) | Not specific | 6 | 0% | 20 | 20.9 | S | NR | China |
|  |  |  | Soy protein isolate (contributes 8% energy) |  | 6 | 0% | 20 | 20.9 | S | NR | China |
|  |  |  | Undigested high molecular fraction of soybean protein (contributes 8% energy) |  | 6 | 0% | 20 | 20.9 | S | NR | China |
| Weisse-2010 (108) | P | 6 (WO: -) | Casein 35 g | Hypercholesterolemic | 21 | 43% | 43 | 25.7 | S | HD | Germany |
|  |  | 6 (WO: -) | Lupin protein 35 g |  | 22 | 50% | 44 | 26.2 | S | HD | Germany |
| Wiebe-1984 (109) | C | 3 (WO: 0) | Animal protein 15 g | Healthy | 8 | 100% | 21 | 21.6 | W | NR | Canada |
|  |  |  | Plant protein 13.8 g |  | 8 | 100% | 21 | 21.6 | W | NR | Canada |
| Xu-2005 (110) | P | 2 (WO: -) | Casein 30 g | Healthy | 9 | 50% | 21 | 22.3 | S | NR | China |
|  |  | 2 (WO: -) | Soy 30 g |  | 9 |  | 21 | 22.3 | S | NR | China |

BMI, Body Mass Index; C, Crossover; EB, Energy balance; ER, Energy restriction; HD, Habitual diet; MetS, Metabolism syndrome; n, Participants number; NR, Not reported; P, Parallel; S, Supplements; W, Whole foods.

**Supplementary data**

# **Supplementary Table 4.** Sensitivity analysis following the removal of single groups or randomized controlled trials to assess the robustness of standard meta-analyses results for quantity-related studies

| **Group excluded** | **Weighted mean difference** | **[95 % CI]** | **I^2^ (%)** | **χ^2^** | ***P* for χ^2^** | **Model** |
| --- | --- | --- | --- | --- | --- | --- |
| **SBP (mmHg)** | | | | | | |
| None | -1.510 | [-2.774, -0.254] | 94.40 | 839.31 | 0.00 | Random |
| Sacks-1984 | -1.557 | [-2.830, -0.284] | 94.50 | 838.08 | 0.00 | Random |
| Jenkins-2000 | -1.491 | [-2.762, -0.219] | 94.50 | 839.29 | 0.00 | Random |
| Brinkworth-2004 | -1.495 | [-2.768, -0.222] | 94.50 | 839.31 | 0.00 | Random |
| Ferrara-2006 | -1.502 | [-2.778, -0.226] | 94.50 | 839.29 | 0.00 | Random |
| Hodgson-2006 | -1.492 | [-2.774, -0.210] | 94.50 | 839.31 | 0.00 | Random |
| McAuley-2006 | -1.571 | [-2.847, -0.294] | 94.50 | 836.55 | 0.00 | Random |
| Delbridge-2009 | -1.452 | [-2.722, -0.181] | 94.50 | 838.55 | 0.00 | Random |
| Papakonstantinou-2010 | -1.333 | [-2.606, -0.060] | 94.30 | 803.02 | 0.00 | Random |
| Gögebakan-2011 | -1.507 | [-3.182, 0.169] | 94.40 | 822.88 | 0.00 | Random |
| Sheikholeslami-2012 | -1.534 | [-2.812, -0.257] | 94.50 | 838.53 | 0.00 | Random |
| Teunisseti-Beekman-2012 | -1.427 | [-2.708, -0.146] | 94.50 | 836.76 | 0.00 | Random |
| Azadbakht-2013 | -1.603 | [-2.893, -0.313] | 94.30 | 801.70 | 0.00 | Random |
| Dalle Grave-2013 | -1.528 | [-2.796, -0.260] | 94.50 | 839.11 | 0.00 | Random |
| Tang-2013 | -1.542 | [-2.816, -0.268] | 94.50 | 838.57 | 0.00 | Random |
| Pedersen-2014 | -1.438 | [-2.713, -0.162] | 94.50 | 838.05 | 0.00 | Random |
| Gulati-2017 | -1.507 | [-2.787, -0.226] | 94.50 | 839.18 | 0.00 | Random |
| Bray-2020 | -1.558 | [-2.824, -0.291] | 94.50 | 838.27 | 0.00 | Random |
| Hudson-2020 | -1.429 | [-2.703, -0.156] | 94.50 | 837.73 | 0.00 | Random |
| Tischmann-2020 | -1.461 | [-2.738, -0.185] | 94.50 | 838.87 | 0.00 | Random |
| Yilmaz-2021 | -1.137 | [-1.916, -0.358] | 76.90 | 199.04 | 0.00 | Random |
| Meckling-2007a | -1.548 | [-2.826, -0.271] | 94.50 | 837.84 | 0.00 | Random |
| Meckling-2007b | -1.553 | [-2.836, -0.270] | 94.50 | 836.09 | 0.00 | Random |
| Claessens-2009a | -1.487 | [-2.753, -0.220] | 94.50 | 839.17 | 0.00 | Random |
| Sacks-2009a | -1.531 | [-2.829, -0.233] | 94.50 | 836.00 | 0.00 | Random |
| Claessens-2009b | -1.444 | [-2.711, -0.177] | 94.50 | 838.03 | 0.00 | Random |
| Sacks-2009b | -1.572 | [-2.862, -0.282] | 94.40 | 827.96 | 0.00 | Random |
| Aldrich-2011a | -1.461 | [-2.728, -0.194] | 94.50 | 838.62 | 0.00 | Random |
| Toscani-2011a | -1.679 | [-2.946, -0.413] | 94.40 | 827.59 | 0.00 | Random |
| Aldrich-2011b | -1.415 | [-2.682, -0.148] | 94.50 | 836.57 | 0.00 | Random |
| Toscani-2011b | -1.527 | [-2.793, -0.260] | 94.50 | 839.14 | 0.00 | Random |
| Weinheimer-2012a | -1.626 | [-2.898, -0.354] | 94.50 | 832.59 | 0.00 | Random |
| Weinheimer-2012b | -1.636 | [-2.908, -0.365] | 94.50 | 830.93 | 0.00 | Random |
| Weinheimer-2012c | -1.592 | [-2.866, -0.318] | 94.50 | 835.45 | 0.00 | Random |
| Weinheimer-2012d | -1.602 | [-2.876, -0.327] | 94.50 | 834.28 | 0.00 | Random |
| Juraschek-2013a | -1.552 | [-2.843, -0.260] | 94.50 | 833.49 | 0.00 | Random |
| Juraschek-2013b | -1.517 | [-2.811, -0.224] | 94.50 | 838.16 | 0.00 | Random |
| de Luis-2015a | -1.543 | [-2.870, -0.215] | 94.40 | 825.92 | 0.00 | Random |
| Hill-2015a | -1.502 | [-2.782, -0.223] | 94.50 | 839.26 | 0.00 | Random |
| de Luis-2015b | -1.588 | [-2.890, -0.287] | 94.30 | 802.83 | 0.00 | Random |
| Hill-2015b | -1.546 | [-2.822, -0.270] | 94.50 | 838.22 | 0.00 | Random |
| Hill-2015c | -1.479 | [-2.758, -0.199] | 94.50 | 839.22 | 0.00 | Random |
| Hill-2015d | -1.549 | [-2.828, -0.271] | 94.50 | 837.65 | 0.00 | Random |
| Hill-2015e | -1.543 | [-2.817, -0.269] | 94.50 | 838.50 | 0.00 | Random |
| Hill-2015f | -1.588 | [-2.863, -0.314] | 94.50 | 835.71 | 0.00 | Random |
| Fekete-2016a | -1.454 | [-2.736, -0.172] | 94.50 | 838.43 | 0.00 | Random |
| Fekete-2016b | -1.539 | [-2.817, -0.261] | 94.50 | 838.30 | 0.00 | Random |
| Mateo-Gallego-2017a | -1.529 | [-2.849, -0.208] | 94.50 | 833.42 | 0.00 | Random |
| Mateo-Gallego-2017b | -1.453 | [-2.734, -0.171] | 94.50 | 838.43 | 0.00 | Random |
| **DBP (mmHg)** | | | | | | |
| None | -1.080 | [-1.805, -0.354] | 85.0 | 314.07 | 0.00 | Random |
| Sacks-1984 | -1.110 | [-1.844, -0.376] | 85.3 | 313.35 | 0.00 | Random |
| Jenkins-2000 | -1.111 | [-1.841, -0.381] | 85.3 | 313.10 | 0.00 | Random |
| Brinkworth-2004 | -1.079 | [-1.813, -0.346] | 85.4 | 314.07 | 0.00 | Random |
| Ferrara-2006 | -1.106 | [-1.837, -0.374] | 85.3 | 313.49 | 0.00 | Random |
| Hodgson-2006 | -1.047 | [-1.784, -0.310] | 85.3 | 313.11 | 0.00 | Random |
| McAuley-2006 | -1.081 | [-1.817, -0.344] | 85.4 | 314.07 | 0.00 | Random |
| Delbridge-2009 | -1.091 | [-1.823, -0.360] | 85.3 | 313.94 | 0.00 | Random |
| Papakonstantinou-2010 | -0.957 | [-1.677, -0.237] | 83.7 | 281.56 | 0.00 | Random |
| Gögebakan-2011 | -1.059 | [-2.001, -0.116] | 85.0 | 307.25 | 0.00 | Random |
| Sheikholeslami-2012 | -1.124 | [-1.859, -0.388] | 85.3 | 312.47 | 0.00 | Random |
| Teunisseti-Beekman-2012 | -1.025 | [-1.765, -0.286] | 85.2 | 310.55 | 0.00 | Random |
| Azadbakht-2013 | -1.179 | [-1.913, -0.446] | 83.8 | 284.69 | 0.00 | Random |
| Dalle Grave-2013 | -1.027 | [-1.757, -0.297] | 85.2 | 311.64 | 0.00 | Random |
| Tang-2013 | -1.080 | [-1.814, -0.347] | 85.4 | 314.07 | 0.00 | Random |
| Pedersen-2014 | -0.984 | [-1.714, -0.255] | 85.0 | 305.99 | 0.00 | Random |
| Gulati-2017 | -1.062 | [-1.803, -0.322] | 85.3 | 313.76 | 0.00 | Random |
| Bray-2020 | -1.097 | [-1.826, -0.368] | 85.3 | 313.68 | 0.00 | Random |
| Hudson-2020 | -1.034 | [-1.770, -0.298] | 85.3 | 312.15 | 0.00 | Random |
| Tischmann-2020 | -1.058 | [-1.795, -0.320] | 85.3 | 313.65 | 0.00 | Random |
| Yilmaz-2021 | -0.848 | [-1.397, -0.298] | 66.4 | 136.95 | 0.00 | Random |
| Meckling-2007a | -1.081 | [-1.819, -0.343] | 85.4 | 314.07 | 0.00 | Random |
| Meckling-2007b | -1.119 | [-1.853, -0.385] | 85.3 | 312.84 | 0.00 | Random |
| Claessens-2009a | -1.057 | [-1.786, -0.328] | 85.3 | 313.38 | 0.00 | Random |
| Sacks-2009a | -1.095 | [-1.851, -0.338] | 85.3 | 313.26 | 0.00 | Random |
| Claessens-2009b | -1.046 | [-1.774, -0.317] | 85.3 | 312.63 | 0.00 | Random |
| Sacks-2009b | -1.152 | [-1.896, -0.409] | 84.7 | 301.27 | 0.00 | Random |
| Aldrich-2011a | -1.056 | [-1.785, -0.326] | 85.3 | 313.43 | 0.00 | Random |
| Toscani-2011a | -1.106 | [-1.835, -0.377] | 85.3 | 313.25 | 0.00 | Random |
| Aldrich-2011b | -1.052 | [-1.779, -0.325] | 85.3 | 312.63 | 0.00 | Random |
| Toscani-2011b | -1.119 | [-1.849, -0.388] | 85.3 | 312.75 | 0.00 | Random |
| Weinheimer-2012a | -1.158 | [-1.892, -0.424] | 85.1 | 308.44 | 0.00 | Random |
| Weinheimer-2012b | -1.123 | [-1.860, -0.387] | 85.3 | 312.38 | 0.00 | Random |
| Weinheimer-2012c | -1.078 | [-1.817, -0.340] | 85.4 | 314.07 | 0.00 | Random |
| Weinheimer-2012d | -1.049 | [-1.786, -0.313] | 85.3 | 313.26 | 0.00 | Random |
| Juraschek-2013a | -1.074 | [-1.833, -0.316] | 85.4 | 314.05 | 0.00 | Random |
| Juraschek-2013b | -1.098 | [-1.855, -0.340] | 85.3 | 312.89 | 0.00 | Random |
| de Luis-2015a | -1.047 | [-1.783, -0.311] | 85.3 | 313.17 | 0.00 | Random |
| Hill-2015a | -1.099 | [-1.837, -0.362] | 85.3 | 313.74 | 0.00 | Random |
| de Luis-2015b | -1.017 | [-1.756, -0.279] | 85.1 | 309.37 | 0.00 | Random |
| Hill-2015b | -1.068 | [-1.805, -0.331] | 85.3 | 313.95 | 0.00 | Random |
| Hill-2015c | -1.109 | [-1.845, -0.373] | 85.3 | 313.35 | 0.00 | Random |
| Hill-2015d | -1.158 | [-1.891, -0.425] | 85.1 | 308.64 | 0.00 | Random |
| Hill-2015e | -1.107 | [-1.842, -0.373] | 85.3 | 313.47 | 0.00 | Random |
| Hill-2015f | -1.127 | [-1.862, -0.392] | 85.3 | 312.20 | 0.00 | Random |
| Fekete-2016a | -1.037 | [-1.780, -0.294] | 85.2 | 311.57 | 0.00 | Random |
| Fekete-2016b | -1.102 | [-1.842, -0.361] | 85.3 | 313.55 | 0.00 | Random |
| Mateo-Gallego-2017a | -1.151 | [-1.886, -0.417] | 85.1 | 309.44 | 0.00 | Random |
| Mateo-Gallego-2017b | -1.126 | [-1.862, -0.389] | 85.3 | 312.16 | 0.00 | Random |
| **Total cholesterol (mmol/L)** | | | | | | |
| None | -0.092 | [-0.145, -0.039] | 74.7 | 217.59 | 0.00 | Random |
| Jenkins-2000 | -0.091 | [-0.145, -0.037] | 75.1 | 216.92 | 0.00 | Random |
| Farnsworth-2003 | -0.092 | [-0.145, -0.038] | 75.0 | 217.49 | 0.00 | Random |
| Brinkworth-2004 | -0.085 | [-0.138, -0.033] | 74.1 | 208.81 | 0.00 | Random |
| Mamo-2005 | -0.089 | [-0.143, -0.035] | 74.8 | 214.35 | 0.00 | Random |
| Noakes-2005 | -0.091 | [-0.145, -0.037] | 75.1 | 216.75 | 0.00 | Random |
| Stephenson-2005 | -0.087 | [-0.140, -0.033] | 74.5 | 211.51 | 0.00 | Random |
| Ferrara-2006 | -0.088 | [-0.141, -0.034] | 74.7 | 213.43 | 0.00 | Random |
| Hodgson-2006 | -0.094 | [-0.148, -0.040] | 75.2 | 217.50 | 0.00 | Random |
| McAuley-2006 | -0.094 | [-0.148, -0.040] | 75.2 | 217.52 | 0.00 | Random |
| Treyzon-2008 | -0.091 | [-0.146, -0.037] | 75.1 | 217.06 | 0.00 | Random |
| Delbridge-2009 | -0.095 | [-0.149, -0.041] | 75.1 | 216.60 | 0.00 | Random |
| Gögebakan-2011 | -0.094 | [-0.149, -0.039] | 75.2 | 217.56 | 0.00 | Random |
| Mehrabani-2012 | -0.092 | [-0.146, -0.038] | 75.2 | 217.49 | 0.00 | Random |
| Sheikholeslami-2012 | -0.100 | [-0.153, -0.046] | 74.2 | 209.53 | 0.00 | Random |
| Azadbakht-2013 | -0.098 | [-0.150, -0.045] | 66.6 | 161.80 | 0.00 | Random |
| Dalle Grave-2013 | -0.092 | [-0.146, -0.038] | 75.2 | 217.47 | 0.00 | Random |
| Tang-2013 | -0.091 | [-0.144, -0.037] | 75.1 | 216.83 | 0.00 | Random |
| Machin-2014 | -0.094 | [-0.148, -0.039] | 75.2 | 217.54 | 0.00 | Random |
| Pedersen-2014 | -0.097 | [-0.151, -0.043] | 74.8 | 214.20 | 0.00 | Random |
| Gulati-2017 | -0.089 | [-0.143, -0.035] | 74.8 | 214.00 | 0.00 | Random |
| Fernandes-2018 | -0.091 | [-0.145, -0.037] | 75.1 | 217.12 | 0.00 | Random |
| Porter-2019 | -0.094 | [-0.148, -0.040] | 75.2 | 217.46 | 0.00 | Random |
| Bray-2020 | -0.098 | [-0.152, -0.044] | 74.6 | 213.02 | 0.00 | Random |
| Hudson-2020 | -0.089 | [-0.142, -0.035] | 74.8 | 214.52 | 0.00 | Random |
| Tischmann-2020 | -0.092 | [-0.146, -0.038] | 75.2 | 217.44 | 0.00 | Random |
| Yilmaz-2021 | -0.077 | [-0.125, -0.029] | 66.1 | 159.52 | 0.00 | Random |
| Meckling-2007a | -0.091 | [-0.144, -0.038] | 74.9 | 215.02 | 0.00 | Random |
| Meckling-2007b | -0.094 | [-0.148, -0.041] | 75.1 | 216.74 | 0.00 | Random |
| Claessens-2009a | -0.091 | [-0.144, -0.037] | 75.1 | 216.80 | 0.00 | Random |
| Claessens-2009b | -0.092 | [-0.146, -0.038] | 75.2 | 217.53 | 0.00 | Random |
| Sacks-2009a | -0.095 | [-0.151, -0.040] | 75.0 | 216.40 | 0.00 | Random |
| Sacks-2009b | -0.094 | [-0.150, -0.039] | 75.2 | 217.42 | 0.00 | Random |
| Toscani-2011a | -0.092 | [-0.145, -0.038] | 75.2 | 217.47 | 0.00 | Random |
| Toscani-2011b | -0.092 | [-0.146, -0.038] | 75.2 | 217.58 | 0.00 | Random |
| Roussell-2012a | -0.094 | [-0.148, -0.039] | 75.2 | 217.57 | 0.00 | Random |
| Roussell-2012b | -0.094 | [-0.148, -0.039] | 75.2 | 217.59 | 0.00 | Random |
| Weinheimer-2012a | -0.094 | [-0.148, -0.040] | 75.2 | 217.50 | 0.00 | Random |
| Weinheimer-2012b | -0.092 | [-0.146, -0.038] | 75.1 | 217.23 | 0.00 | Random |
| Weinheimer-2012c | -0.092 | [-0.146, -0.038] | 75.1 | 217.21 | 0.00 | Random |
| Weinheimer-2012d | -0.090 | [-0.144, -0.036] | 74.9 | 215.34 | 0.00 | Random |
| Juraschek-2013a | -0.091 | [-0.146, -0.037] | 75.0 | 215.88 | 0.00 | Random |
| Juraschek-2013b | -0.089 | [-0.143, -0.035] | 74.4 | 210.62 | 0.00 | Random |
| Chiu-2014a | -0.092 | [-0.146, -0.038] | 75.2 | 217.36 | 0.00 | Random |
| Chiu-2014b | -0.091 | [-0.146, -0.037] | 75.1 | 217.06 | 0.00 | Random |
| de Luis-2015a | -0.098 | [-0.152, -0.043] | 74.1 | 208.82 | 0.00 | Random |
| de Luis-2015b | -0.092 | [-0.147, -0.037] | 75.0 | 215.78 | 0.00 | Random |
| Hill-2015a | -0.092 | [-0.146, -0.037] | 75.1 | 217.13 | 0.00 | Random |
| Hill-2015b | -0.087 | [-0.141, -0.034] | 74.6 | 212.41 | 0.00 | Random |
| Hill-2015c | -0.093 | [-0.147, -0.039] | 75.2 | 217.56 | 0.00 | Random |
| Hill-2015d | -0.096 | [-0.150, -0.042] | 75.0 | 215.77 | 0.00 | Random |
| Hill-2015e | -0.095 | [-0.149, -0.041] | 75.1 | 217.03 | 0.00 | Random |
| Hill-2015f | -0.096 | [-0.150, -0.042] | 75.0 | 216.22 | 0.00 | Random |
| Fekete-2016a | -0.089 | [-0.143, -0.035] | 74.9 | 215.00 | 0.00 | Random |
| Fekete-2016b | -0.090 | [-0.144, -0.036] | 75.0 | 216.19 | 0.00 | Random |
| Mateo-Gallego-2017a | -0.083 | [-0.135, -0.032] | 70.7 | 184.18 | 0.00 | Random |
| Mateo-Gallego-2017b | -0.092 | [-0.146, -0.038] | 75.2 | 217.59 | 0.00 | Random |
| **Triglyceride (mmol/L)** | | | | | | |
| None | -0.049 | [-0.100, 0.003] | 83.8 | 328.13 | 0.00 | Random |
| Jenkins-2000 | -0.050 | [-0.102, 0.001] | 84.1 | 327.62 | 0.00 | Random |
| Farnsworth-2003 | -0.047 | [-0.098, 0.005] | 84.1 | 327.01 | 0.00 | Random |
| Brinkworth-2004 | -0.046 | [-0.098, 0.006] | 84.1 | 326.25 | 0.00 | Random |
| Mamo-2005 | -0.044 | [-0.096, 0.008] | 83.9 | 323.34 | 0.00 | Random |
| Noakes-2005 | -0.046 | [-0.098, 0.006] | 84.1 | 326.23 | 0.00 | Random |
| Stephenson-2005 | -0.047 | [-0.099, 0.005] | 84.1 | 327.05 | 0.00 | Random |
| Ferrara-2006 | -0.048 | [-0.100, 0.004] | 84.1 | 327.86 | 0.00 | Random |
| Hodgson-2006 | -0.049 | [-0.101, 0.003] | 84.2 | 328.10 | 0.00 | Random |
| McAuley-2006 | -0.045 | [-0.097, 0.007] | 84.0 | 324.74 | 0.00 | Random |
| Treyzon-2008 | -0.047 | [-0.099, 0.005] | 84.1 | 327.21 | 0.00 | Random |
| Delbridge-2009 | -0.047 | [-0.099, 0.004] | 84.1 | 327.56 | 0.00 | Random |
| Papakonstantinou-2010 | -0.048 | [-0.100, 0.005] | 84.1 | 326.36 | 0.00 | Random |
| Gögebakan-2011 | -0.051 | [-0.104, 0.001] | 84.1 | 326.18 | 0.00 | Random |
| Mehrabani-2012 | -0.051 | [-0.103, 0.002] | 84.1 | 327.76 | 0.00 | Random |
| Sheikholeslami-2012 | -0.055 | [-0.107, 0.003] | 83.5 | 315.01 | 0.00 | Random |
| Azadbakht-2013 | -0.052 | [-0.114, 0.010] | 83.4 | 313.61 | 0.00 | Random |
| Dalle Grave-2013 | -0.047 | [-0.099, 0.004] | 84.1 | 327.61 | 0.00 | Random |
| Tang-2013 | -0.047 | [-0.099, 0.004] | 84.1 | 327.19 | 0.00 | Random |
| Machin-2014 | -0.049 | [-0.101, 0.004] | 84.1 | 328.03 | 0.00 | Random |
| Pedersen-2014 | -0.046 | [-0.098, 0.006] | 84.1 | 326.27 | 0.00 | Random |
| Gulati-2017 | -0.073 | [-0.114, 0.031] | 72.0 | 185.93 | 0.00 | Random |
| Fernandes-2018 | -0.051 | [-0.103, 0.001] | 84.1 | 327.74 | 0.00 | Random |
| Porter-2019 | -0.049 | [-0.101, 0.004] | 84.1 | 328.02 | 0.00 | Random |
| Bray-2020 | -0.045 | [-0.097, 0.007] | 84.0 | 324.12 | 0.00 | Random |
| Hudson-2020 | -0.047 | [-0.099, 0.005] | 84.1 | 327.05 | 0.00 | Random |
| Yilmaz-2021 | -0.048 | [-0.103, 0.007] | 83.9 | 323.33 | 0.00 | Random |
| Meckling-2007a | -0.050 | [-0.101, 0.002] | 84.1 | 327.94 | 0.00 | Random |
| Meckling-2007b | -0.038 | [-0.089, 0.013] | 82.8 | 302.63 | 0.00 | Random |
| Claessens-2009a | -0.045 | [-0.097, 0.006] | 84.0 | 324.41 | 0.00 | Random |
| Claessens-2009b | -0.045 | [-0.097, 0.006] | 84.0 | 324.17 | 0.00 | Random |
| Sacks-2009a | -0.049 | [-0.101, 0.004] | 84.1 | 327.92 | 0.00 | Random |
| Sacks-2009b | -0.049 | [-0.102, 0.004] | 84.1 | 328.05 | 0.00 | Random |
| Roussell-2012a | -0.048 | [-0.101, 0.005] | 84.1 | 327.17 | 0.00 | Random |
| Roussell-2012b | -0.049 | [-0.102, 0.004] | 84.1 | 328.00 | 0.00 | Random |
| Weinheimer-2012a | -0.050 | [-0.102, 0.002] | 84.2 | 328.09 | 0.00 | Random |
| Weinheimer-2012b | -0.050 | [-0.102, 0.002] | 84.2 | 328.08 | 0.00 | Random |
| Weinheimer-2012c | -0.051 | [-0.103, 0.000] | 84.1 | 326.91 | 0.00 | Random |
| Weinheimer-2012d | -0.052 | [-0.103, 0.000] | 84.1 | 326.80 | 0.00 | Random |
| Juraschek-2013a | -0.048 | [-0.101, 0.004] | 84.1 | 327.69 | 0.00 | Random |
| Juraschek-2013b | -0.050 | [-0.103, 0.003] | 84.1 | 327.73 | 0.00 | Random |
| Chiu-2014a | -0.052 | [-0.104, 0.000] | 84.1 | 326.82 | 0.00 | Random |
| Chiu-2014b | -0.048 | [-0.100, 0.004] | 84.1 | 327.88 | 0.00 | Random |
| de Luis-2015a | -0.049 | [-0.102, 0.004] | 84.1 | 328.03 | 0.00 | Random |
| de Luis-2015b | -0.053 | [-0.106, 0.001] | 83.9 | 322.44 | 0.00 | Random |
| Hill-2015a | -0.046 | [-0.098, 0.006] | 84.0 | 325.63 | 0.00 | Random |
| Hill-2015b | -0.047 | [-0.099, 0.005] | 84.1 | 327.12 | 0.00 | Random |
| Hill-2015c | -0.056 | [-0.107, 0.005] | 83.7 | 319.38 | 0.00 | Random |
| Hill-2015d | -0.053 | [-0.104, 0.001] | 84.0 | 325.84 | 0.00 | Random |
| Hill-2015e | -0.051 | [-0.103, 0.001] | 84.1 | 327.53 | 0.00 | Random |
| Hill-2015f | -0.056 | [-0.107, 0.005] | 83.7 | 319.25 | 0.00 | Random |
| Fekete-2016a | -0.045 | [-0.097, 0.007] | 84.0 | 324.10 | 0.00 | Random |
| Fekete-2016b | -0.046 | [-0.098, 0.006] | 84.1 | 326.68 | 0.00 | Random |
| Mateo-Gallego-2017a | -0.040 | [-0.090, 0.009] | 80.5 | 266.31 | 0.00 | Random |
| Mateo-Gallego-2017b | -0.048 | [-0.100, 0.004] | 83.8 | 327.81 | 0.00 | Random |
| **LDL-cholesterol (mmol/L)** | | | | | | |
| None | -0.045 | [-0.109, 0.019] | 89.3 | 497.45 | 0.00 | Random |
| Jenkins-2000 | -0.043 | [-0.107, 0.021] | 89.5 | 497.13 | 0.00 | Random |
| Farnsworth-2003 | -0.045 | [-0.109, 0.019] | 89.5 | 497.45 | 0.00 | Random |
| Brinkworth-2004 | -0.041 | [-0.105, 0.023] | 89.5 | 495.43 | 0.00 | Random |
| Noakes-2005 | -0.044 | [-0.109, 0.020] | 89.5 | 497.45 | 0.00 | Random |
| Stephenson-2005 | -0.041 | [-0.106, 0.023] | 89.5 | 496.19 | 0.00 | Random |
| Hodgson-2006 | -0.050 | [-0.114, 0.015] | 89.5 | 493.73 | 0.00 | Random |
| McAuley-2006 | -0.044 | [-0.108, 0.021] | 89.5 | 497.38 | 0.00 | Random |
| Treyzon-2008 | -0.047 | [-0.111, 0.018] | 89.5 | 496.71 | 0.00 | Random |
| Delbridge-2009 | -0.046 | [-0.109, 0.018] | 89.5 | 496.02 | 0.00 | Random |
| Papakonstantinou-2010 | -0.040 | [-0.105, 0.025] | 89.4 | 491.01 | 0.00 | Random |
| Gögebakan-2011 | -0.045 | [-0.111, 0.020] | 89.5 | 497.06 | 0.00 | Random |
| Toscani-2011a | -0.044 | [-0.109, 0.020] | 89.5 | 497.44 | 0.00 | Random |
| Toscani-2011b | -0.044 | [-0.108, 0.020] | 89.5 | 497.42 | 0.00 | Random |
| Mehrabani-2012 | -0.044 | [-0.109, 0.020] | 89.5 | 497.45 | 0.00 | Random |
| Sheikholeslami-2012 | -0.054 | [-0.118, 0.010] | 89.3 | 486.44 | 0.00 | Random |
| Azadbakht-2013 | -0.048 | [-0.115, 0.020] | 86.8 | 392.54 | 0.00 | Random |
| Dalle Grave-2013 | -0.043 | [-0.107, 0.021] | 89.5 | 497.14 | 0.00 | Random |
| Tang-2013 | -0.045 | [-0.109, 0.020] | 89.5 | 497.45 | 0.00 | Random |
| Pedersen-2014 | -0.048 | [-0.113, 0.016] | 89.5 | 494.71 | 0.00 | Random |
| Gulati-2017 | -0.039 | [-0.103, 0.026] | 89.4 | 492.41 | 0.00 | Random |
| Fernandes-2018 | -0.042 | [-0.106, 0.022] | 89.5 | 496.72 | 0.00 | Random |
| Porter-2019 | -0.047 | [-0.111, 0.018] | 89.5 | 496.58 | 0.00 | Random |
| Bray-2020 | -0.049 | [-0.113, 0.015] | 89.5 | 494.91 | 0.00 | Random |
| Hudson-2020 | -0.044 | [-0.109, 0.021] | 89.5 | 497.42 | 0.00 | Random |
| Tischmann-2020 | -0.045 | [-0.110, 0.019] | 89.5 | 497.35 | 0.00 | Random |
| Yilmaz-2021 | -0.027 | [-0.064, 0.009] | 48.7 | 101.30 | 0.00 | Random |
| Meckling-2007a | -0.044 | [-0.107, 0.020] | 89.5 | 495.01 | 0.00 | Random |
| Meckling-2007b | -0.046 | [-0.110, 0.018] | 89.5 | 497.04 | 0.00 | Random |
| Claessens-2009a | -0.050 | [-0.115, 0.014] | 89.4 | 492.89 | 0.00 | Random |
| Claessens-2009b | -0.046 | [-0.111, 0.018] | 89.5 | 497.06 | 0.00 | Random |
| Sacks-2009a | -0.048 | [-0.113, 0.018] | 89.5 | 493.52 | 0.00 | Random |
| Sacks-2009b | -0.045 | [-0.111, 0.020] | 89.5 | 497.13 | 0.00 | Random |
| Roussell-2012a | -0.046 | [-0.111, 0.019] | 89.5 | 496.86 | 0.00 | Random |
| Roussell-2012b | -0.046 | [-0.111, 0.019] | 89.5 | 497.02 | 0.00 | Random |
| Weinheimer-2012a | -0.044 | [-0.108, 0.021] | 89.5 | 497.36 | 0.00 | Random |
| Weinheimer-2012b | -0.044 | [-0.108, 0.021] | 89.5 | 497.36 | 0.00 | Random |
| Weinheimer-2012c | -0.044 | [-0.108, 0.021] | 89.5 | 497.35 | 0.00 | Random |
| Weinheimer-2012d | -0.044 | [-0.108, 0.021] | 89.5 | 497.35 | 0.00 | Random |
| Juraschek-2013a | -0.047 | [-0.112, 0.018] | 89.5 | 495.22 | 0.00 | Random |
| Juraschek-2013b | -0.045 | [-0.110, 0.021] | 89.5 | 497.35 | 0.00 | Random |
| Chiu-2014a | -0.043 | [-0.108, 0.022] | 89.5 | 497.17 | 0.00 | Random |
| Chiu-2014b | -0.043 | [-0.108, 0.021] | 89.5 | 497.31 | 0.00 | Random |
| de Luis-2015a | -0.047 | [-0.115, 0.022] | 89.0 | 471.18 | 0.00 | Random |
| de Luis-2015b | -0.046 | [-0.111, 0.019] | 89.5 | 496.46 | 0.00 | Random |
| Hill-2015a | -0.045 | [-0.109, 0.019] | 89.5 | 497.45 | 0.00 | Random |
| Hill-2015b | -0.040 | [-0.104, 0.024] | 89.5 | 495.05 | 0.00 | Random |
| Hill-2015c | -0.045 | [-0.109, 0.020] | 89.5 | 497.45 | 0.00 | Random |
| Hill-2015d | -0.049 | [-0.113, 0.015] | 89.5 | 494.94 | 0.00 | Random |
| Hill-2015e | -0.043 | [-0.108, 0.022] | 89.5 | 497.13 | 0.00 | Random |
| Hill-2015f | -0.047 | [-0.111, 0.018] | 89.5 | 496.96 | 0.00 | Random |
| Fekete-2016a | -0.044 | [-0.108, 0.021] | 89.5 | 497.37 | 0.00 | Random |
| Fekete-2016b | -0.045 | [-0.109, 0.020] | 89.5 | 497.43 | 0.00 | Random |
| Mateo-Gallego-2017a | -0.046 | [-0.110, 0.018] | 89.5 | 497.29 | 0.00 | Random |
| Mateo-Gallego-2017b | -0.043 | [-0.111, 0.026] | 89.5 | 493.77 | 0.00 | Random |
| **HDL-cholesterol (mmol/L)** | | | | | | |
| None | -0.005 | [-0.022, 0.013] | 91.7 | 671.99 | 0.00 | Random |
| Jenkins-2000 | -0.003 | [-0.021, 0.014] | 91.8 | 667.44 | 0.00 | Random |
| Farnsworth-2003 | -0.005 | [-0.022, 0.013] | 91.8 | 671.99 | 0.00 | Random |
| Brinkworth-2004 | -0.005 | [-0.023, 0.013] | 91.8 | 671.95 | 0.00 | Random |
| Mamo-2005 | -0.005 | [-0.022, 0.013] | 91.8 | 671.87 | 0.00 | Random |
| Noakes-2005 | -0.005 | [-0.022, 0.013] | 91.8 | 671.92 | 0.00 | Random |
| Stephenson-2005 | -0.004 | [-0.021, 0.014] | 91.8 | 670.32 | 0.00 | Random |
| Ferrara-2006 | -0.005 | [-0.023, 0.012] | 91.8 | 668.87 | 0.00 | Random |
| Hodgson-2006 | -0.005 | [-0.022, 0.013] | 91.8 | 671.99 | 0.00 | Random |
| McAuley-2006 | -0.005 | [-0.022, 0.013] | 91.8 | 671.99 | 0.00 | Random |
| Treyzon-2008 | -0.004 | [-0.021, 0.014] | 91.8 | 670.24 | 0.00 | Random |
| Delbridge-2009 | -0.005 | [-0.022, 0.012] | 91.8 | 671.93 | 0.00 | Random |
| Papakonstantinou-2010 | -0.002 | [-0.017, 0.013] | 86.2 | 399.06 | 0.00 | Random |
| Gögebakan-2011 | -0.004 | [-0.022, 0.013] | 91.8 | 670.61 | 0.00 | Random |
| Toscani-2011a | -0.001 | [-0.018, 0.016] | 91.4 | 641.91 | 0.00 | Random |
| Toscani-2011b | -0.004 | [-0.022, 0.013] | 91.8 | 671.82 | 0.00 | Random |
| Mehrabani-2012 | -0.005 | [-0.023, 0.012] | 91.8 | 671.84 | 0.00 | Random |
| Sheikholeslami-2012 | -0.005 | [-0.023, 0.012] | 91.8 | 671.54 | 0.00 | Random |
| Azadbakht-2013 | -0.004 | [-0.027, 0.018] | 91.8 | 671.97 | 0.00 | Random |
| Dalle Grave-2013 | -0.004 | [-0.022, 0.013] | 91.8 | 671.61 | 0.00 | Random |
| Tang-2013 | -0.005 | [-0.022, 0.013] | 91.8 | 671.95 | 0.00 | Random |
| Machin-2014 | -0.004 | [-0.021, 0.014] | 91.8 | 669.58 | 0.00 | Random |
| Pedersen-2014 | -0.005 | [-0.022, 0.013] | 91.8 | 671.96 | 0.00 | Random |
| Gulati-2017 | -0.003 | [-0.021, 0.014] | 91.7 | 666.66 | 0.00 | Random |
| Fernandes-2018 | -0.005 | [-0.022, 0.013] | 91.8 | 671.97 | 0.00 | Random |
| Porter-2019 | -0.005 | [-0.023, 0.012] | 91.8 | 671.61 | 0.00 | Random |
| Bray-2020 | -0.006 | [-0.023, 0.011] | 91.8 | 669.38 | 0.00 | Random |
| Hudson-2020 | -0.005 | [-0.022, 0.013] | 91.8 | 671.96 | 0.00 | Random |
| Tischmann-2020 | -0.005 | [-0.022, 0.013] | 91.8 | 671.97 | 0.00 | Random |
| Yilmaz-2021 | -0.007 | [-0.024, 0.009] | 86.6 | 410.91 | 0.00 | Random |
| Meckling-2007a | -0.005 | [-0.023, 0.012] | 91.8 | 671.40 | 0.00 | Random |
| Meckling-2007b | -0.006 | [-0.023, 0.011] | 91.7 | 661.26 | 0.00 | Random |
| Claessens-2009a | -0.006 | [-0.023, 0.011] | 91.8 | 668.33 | 0.00 | Random |
| Claessens-2009b | -0.006 | [-0.023, 0.012] | 91.8 | 670.71 | 0.00 | Random |
| Sacks-2009a | -0.005 | [-0.023, 0.012] | 91.8 | 671.28 | 0.00 | Random |
| Sacks-2009b | -0.006 | [-0.024, 0.011] | 91.7 | 662.57 | 0.00 | Random |
| Roussell-2012a | -0.005 | [-0.022, 0.012] | 91.8 | 671.91 | 0.00 | Random |
| Roussell-2012b | -0.005 | [-0.022, 0.013] | 91.8 | 671.96 | 0.00 | Random |
| Weinheimer-2012a | -0.005 | [-0.022, 0.013] | 91.8 | 671.95 | 0.00 | Random |
| Weinheimer-2012b | -0.005 | [-0.022, 0.013] | 91.8 | 671.95 | 0.00 | Random |
| Weinheimer-2012c | -0.006 | [-0.024, 0.011] | 91.7 | 666.61 | 0.00 | Random |
| Weinheimer-2012d | -0.006 | [-0.024, 0.011] | 91.7 | 666.56 | 0.00 | Random |
| Juraschek-2013a | -0.003 | [-0.021, 0.014] | 91.7 | 658.96 | 0.00 | Random |
| Juraschek-2013b | -0.004 | [-0.021, 0.014] | 91.8 | 668.27 | 0.00 | Random |
| Chiu-2014a | -0.004 | [-0.022, 0.013] | 91.8 | 670.21 | 0.00 | Random |
| Chiu-2014b | -0.004 | [-0.022, 0.013] | 91.8 | 671.60 | 0.00 | Random |
| de Luis-2015a | -0.006 | [-0.024, 0.011] | 91.8 | 666.77 | 0.00 | Random |
| de Luis-2015b | -0.006 | [-0.023, 0.012] | 91.8 | 670.68 | 0.00 | Random |
| Hill-2015a | -0.003 | [-0.021, 0.014] | 91.7 | 665.70 | 0.00 | Random |
| Hill-2015b | -0.004 | [-0.021, 0.014] | 91.8 | 668.97 | 0.00 | Random |
| Hill-2015c | -0.004 | [-0.022, 0.013] | 91.8 | 670.02 | 0.00 | Random |
| Hill-2015d | -0.004 | [-0.021, 0.014] | 91.8 | 667.79 | 0.00 | Random |
| Hill-2015e | -0.005 | [-0.022, 0.013] | 91.8 | 671.98 | 0.00 | Random |
| Hill-2015f | -0.004 | [-0.022, 0.013] | 91.8 | 671.64 | 0.00 | Random |
| Fekete-2016a | -0.004 | [-0.021, 0.014] | 91.8 | 667.43 | 0.00 | Random |
| Fekete-2016b | -0.003 | [-0.021, 0.014] | 91.8 | 667.40 | 0.00 | Random |
| Mateo-Gallego-2017a | -0.001 | [-0.019, 0.016] | 91.4 | 638.39 | 0.00 | Random |
| Mateo-Gallego-2017b | -0.005 | [-0.023, 0.013] | 91.8 | 670.65 | 0.00 | Random |
| **CRP (mg/L)** | | | | | | |
| None | -0.159 | [ -0.509, 0.192] | 67.9 | 65.35 | 0.000 | Random |
| Brinkworth-2004 | -0.085 | [ -0.407, 0.236] | 45.8 | 36.93 | 0.012 | Random |
| Noakes-2005 | -0.113 | [ -0.472, 0.245] | 67.6 | 61.76 | 0.000 | Random |
| Mehrabani-2012 | -0.115 | [ -0.476, 0.245] | 67.6 | 61.75 | 0.000 | Random |
| Azadbakht-2013 | -0.251 | [ -0.552, 0.049] | 33.6 | 30.12 | 0.068 | Random |
| Fernandes-2018 | -0.168 | [ -0.535, 0.199] | 69.4 | 65.34 | 0.000 | Random |
| Yilmaz-2021 | -0.114 | [ -0.442, 0.214] | 63.5 | 54.85 | 0.000 | Random |
| Roussell-2012a | -0.159 | [ -0.516, 0.198] | 69.4 | 65.32 | 0.000 | Random |
| Roussell-2012b | -0.160 | [ -0.517, 0.197] | 69.4 | 65.34 | 0.000 | Random |
| Weinheimer-2012a | -0.192 | [ -0.549, 0.165] | 68.9 | 64.34 | 0.000 | Random |
| Weinheimer-2012b | -0.148 | [ -0.509, 0.213] | 69.2 | 64.94 | 0.000 | Random |
| Weinheimer-2012c | -0.187 | [ -0.547, 0.172] | 69.1 | 64.80 | 0.000 | Random |
| Weinheimer-2012d | -0.129 | [ -0.490, 0.232] | 68.6 | 63.60 | 0.000 | Random |
| de Luis-2015a | -0.162 | [ -0.513, 0.190] | 69.2 | 64.89 | 0.000 | Random |
| de Luis-2015b | -0.160 | [ -0.512, 0.193] | 69.4 | 65.32 | 0.000 | Random |
| Hill-2015a | -0.208 | [ -0.565, 0.150] | 68.5 | 63.41 | 0.000 | Random |
| Hill-2015b | -0.137 | [ -0.498, 0.224] | 68.9 | 64.26 | 0.000 | Random |
| Hill-2015c | -0.165 | [ -0.525, 0.196] | 69.4 | 65.35 | 0.000 | Random |
| Hill-2015d | -0.182 | [ -0.546, 0.182] | 69.3 | 65.15 | 0.000 | Random |
| Hill-2015e | -0.164 | [ -0.525, 0.196] | 69.4 | 65.35 | 0.000 | Random |
| Hill-2015f | -0.179 | [ -0.545, 0.187] | 69.4 | 65.26 | 0.000 | Random |
| Fekete-2016a | -0.147 | [ -0.525, 0.230] | 68.7 | 63.92 | 0.000 | Random |
| Fekete-2016b | -0.183 | [ -0.557, 0.191] | 69.3 | 65.25 | 0.000 | Random |

**Supplementary data**

# **Supplementary Table 5.** Sensitivity analysis following the removal of single groups or randomized controlled trials to assess the robustness of standard meta-analyses results for type-related studies

| **Group excluded** | **Weighted mean difference** | **[95 % CI]** | **I^2^ (%)** | **χ^2^** | ***P* for χ^2^** | **Model** |
| --- | --- | --- | --- | --- | --- | --- |
| **SBP (mmHg)** | | | | | | |
| None | -1.122 | [-2.511, 0.267] | 69.6 | 98.53 | 0.00 | Random |
| Prescott-1987 | -1.345 | [-2.720, 0.030] | 67.8 | 90.07 | 0.00 | Random |
| Kurowska-1997 | -1.335 | [-2.713, 0.042] | 68.3 | 91.39 | 0.00 | Random |
| Vigna-2000 | -1.246 | [-2.638, 0.147] | 69.7 | 95.65 | 0.00 | Random |
| Teede-2001 | -0.991 | [-2.431, 0.448] | 70.0 | 96.82 | 0.00 | Random |
| Rivas-2002 | -0.827 | [-2.035, 0.380] | 58.8 | 70.40 | 0.00 | Random |
| Cuevas-2003 | -1.137 | [-2.547, 0.274] | 70.5 | 98.35 | 0.00 | Random |
| Liao-2007 | -1.177 | [-2.590, 0.236] | 70.3 | 97.68 | 0.00 | Random |
| Azadbakht-2008 | -1.130 | [-2.527, 0.268] | 70.5 | 98.39 | 0.00 | Random |
| Maki-2010 | -1.055 | [-2.509, 0.399] | 70.5 | 98.47 | 0.00 | Random |
| Santo-2010a | -1.172 | [-2.589, 0.245] | 70.3 | 97.71 | 0.00 | Random |
| Santo-2010b | -1.096 | [-2.517, 0.326] | 70.6 | 98.53 | 0.00 | Random |
| Azadbakht-2011 | -1.047 | [-2.487, 0.393] | 70.5 | 98.32 | 0.00 | Random |
| Liu-2012a | -1.084 | [-2.535, 0.368] | 70.6 | 98.50 | 0.00 | Random |
| Liu-2012b | -1.167 | [-2.605, 0.270] | 70.1 | 97.02 | 0.00 | Random |
| Bahr-2013 | -1.079 | [-2.499, 0.341] | 70.6 | 98.49 | 0.00 | Random |
| Liu-2013a | -1.042 | [-2.486, 0.403] | 70.5 | 98.28 | 0.00 | Random |
| Liu-2013b | -0.981 | [-2.414, 0.451] | 69.9 | 96.40 | 0.00 | Random |
| Liu-2013c | -1.177 | [-2.601, 0.247] | 70.2 | 97.39 | 0.00 | Random |
| Liu-2013d | -1.193 | [-2.611, 0.224] | 70.1 | 97.11 | 0.00 | Random |
| Bahr-2015 | -1.220 | [-2.621, 0.180] | 70.0 | 96.57 | 0.00 | Random |
| Hill-2015a | -1.080 | [-2.513, 0.354] | 70.6 | 98.52 | 0.00 | Random |
| Hill-2015b | -1.152 | [-2.581, 0.277] | 70.4 | 97.88 | 0.00 | Random |
| Hill-2015c | -1.033 | [-2.474, 0.408] | 70.4 | 98.11 | 0.00 | Random |
| Hill-2015d | -1.155 | [-2.592, 0.282] | 70.3 | 97.51 | 0.00 | Random |
| Hill-2015e | -1.080 | [-2.513, 0.354] | 70.6 | 98.52 | 0.00 | Random |
| Hill-2015f | -1.080 | [-2.513, 0.354] | 70.6 | 98.52 | 0.00 | Random |
| Tahavorgar-2015 | -1.484 | [-2.757, 0.211] | 63.7 | 79.81 | 0.00 | Random |
| Sucher-2017 | -0.898 | [-2.232, 0.436] | 63.7 | 79.98 | 0.00 | Random |
| Basciani-2020a | -1.051 | [-2.526, 0.425] | 70.6 | 98.51 | 0.00 | Random |
| Basciani-2020b | -1.051 | [-2.525, 0.423] | 70.6 | 98.51 | 0.00 | Random |
| Crimarco-2020 | -1.222 | [-2.644, 0.200] | 69.5 | 95.15 | 0.00 | Random |
| **DBP (mmHg)** | | | | | | |
| None | -0.268 | [-1.576, 1.040] | 80.0 | 150.20 | 0.00 | Random |
| Prescott-1987 | -1.345 | [-2.720, 0.030] | 67.8 | 90.07 | 0.00 | Random |
| Kurowska-1997 | -1.335 | [-2.713, 0.042] | 68.3 | 91.39 | 0.00 | Random |
| Vigna-2000 | -1.246 | [-2.638, 0.147] | 69.7 | 95.65 | 0.00 | Random |
| Teede-2001 | -0.991 | [-2.431, 0.448] | 70.0 | 96.82 | 0.00 | Random |
| Rivas-2002 | -0.827 | [-2.035, 0.380] | 58.8 | 70.40 | 0.00 | Random |
| Cuevas-2003 | -1.137 | [-2.547, 0.274] | 70.5 | 98.35 | 0.00 | Random |
| Liao-2007 | -1.177 | [-2.590, 0.236] | 70.3 | 97.68 | 0.00 | Random |
| Azadbakht-2008 | -1.130 | [-2.527, 0.268] | 70.5 | 98.39 | 0.00 | Random |
| Maki-2010 | -1.055 | [-2.509, 0.399] | 70.5 | 98.47 | 0.00 | Random |
| Santo-2010a | -1.172 | [-2.589, 0.245] | 70.3 | 97.71 | 0.00 | Random |
| Santo-2010b | -1.096 | [-2.517, 0.326] | 70.6 | 98.53 | 0.00 | Random |
| Azadbakht-2011 | -1.047 | [-2.487, 0.393] | 70.5 | 98.32 | 0.00 | Random |
| Liu-2012a | -1.084 | [-2.535, 0.368] | 70.6 | 98.50 | 0.00 | Random |
| Liu-2012b | -1.167 | [-2.605, 0.270] | 70.1 | 97.02 | 0.00 | Random |
| Bahr-2013 | -1.079 | [-2.499, 0.341] | 70.6 | 98.49 | 0.00 | Random |
| Liu-2013a | -1.042 | [-2.486, 0.403] | 70.5 | 98.28 | 0.00 | Random |
| Liu-2013b | -0.981 | [-2.414, 0.451] | 69.9 | 96.40 | 0.00 | Random |
| Liu-2013c | -1.177 | [-2.601, 0.247] | 70.2 | 97.39 | 0.00 | Random |
| Liu-2013d | -1.193 | [-2.611, 0.224] | 70.1 | 97.11 | 0.00 | Random |
| Bahr-2015 | -1.220 | [-2.621, 0.180] | 70.0 | 96.57 | 0.00 | Random |
| Hill-2015a | -1.080 | [-2.513, 0.354] | 70.6 | 98.52 | 0.00 | Random |
| Hill-2015b | -1.152 | [-2.581, 0.277] | 70.4 | 97.88 | 0.00 | Random |
| Hill-2015c | -1.033 | [-2.474, 0.408] | 70.4 | 98.11 | 0.00 | Random |
| Hill-2015d | -1.155 | [-2.592, 0.282] | 70.3 | 97.51 | 0.00 | Random |
| Hill-2015e | -1.080 | [-2.513, 0.354] | 70.6 | 98.52 | 0.00 | Random |
| Hill-2015f | -1.080 | [-2.513, 0.354] | 70.6 | 98.52 | 0.00 | Random |
| Tahavorgar-2015 | -1.484 | [-2.757, 0.211] | 63.7 | 79.81 | 0.00 | Random |
| Sucher-2017 | -0.898 | [-2.232, 0.436] | 63.7 | 79.98 | 0.00 | Random |
| Basciani-2020a | -1.051 | [-2.526, 0.425] | 70.6 | 98.51 | 0.00 | Random |
| Basciani-2020b | -1.051 | [-2.525, 0.423] | 70.6 | 98.51 | 0.00 | Random |
| Crimarco-2020 | -1.222 | [-2.644, 0.200] | 69.5 | 95.15 | 0.00 | Random |
| **Total cholesterol (mmol/L)** | | | | | | |
| None | -0.120 | [-0.190, -0.051] | 92.2 | 931.49 | 0.00 | Random |
| van Raaij-1981 | -0.122 | [-0.192, -0.052] | 92.3 | 931.43 | 0.00 | Random |
| van Raaij-1982a | -0.120 | [-0.190, -0.050] | 92.2 | 928.45 | 0.00 | Random |
| van Raaij-1982b | -0.124 | [-0.194, -0.054] | 92.3 | 930.90 | 0.00 | Random |
| Bosello-1988 | -0.114 | [-0.183, -0.045] | 92.0 | 902.54 | 0.00 | Random |
| Bakhit-1994a | -0.119 | [-0.189, -0.049] | 92.2 | 927.05 | 0.00 | Random |
| Bakhit-1994b | -0.123 | [-0.193, -0.053] | 92.3 | 931.49 | 0.00 | Random |
| Wang-1995a | -0.124 | [-0.193, -0.054] | 92.3 | 930.26 | 0.00 | Random |
| Wang-1995b | -0.119 | [-0.188, -0.049] | 92.3 | 929.86 | 0.00 | Random |
| Kurowska-1997 | -0.123 | [-0.193, -0.053] | 92.3 | 931.35 | 0.00 | Random |
| Nilausen-1998 | -0.121 | [-0.191, -0.052] | 92.3 | 931.42 | 0.00 | Random |
| Shige-1998 | -0.122 | [-0.192, -0.052] | 92.3 | 931.46 | 0.00 | Random |
| Ashton-2000 | -0.119 | [-0.189, -0.049] | 92.2 | 923.17 | 0.00 | Random |
| Jenkins-2000 | -0.116 | [-0.185, -0.047] | 92.2 | 918.31 | 0.00 | Random |
| Teixeira-2000a | -0.116 | [-0.186, -0.047] | 92.2 | 920.51 | 0.00 | Random |
| Teixeira-2000b | -0.122 | [-0.201, -0.043] | 92.3 | 929.31 | 0.00 | Random |
| Vigna-2000 | -0.122 | [-0.192, -0.052] | 92.3 | 931.41 | 0.00 | Random |
| Gardner-2001a | -0.125 | [-0.195, -0.055] | 92.2 | 928.93 | 0.00 | Random |
| Gardner-2001b | -0.122 | [-0.192, -0.052] | 92.3 | 931.42 | 0.00 | Random |
| Teede-2001 | -0.120 | [-0.190, -0.050] | 92.3 | 929.62 | 0.00 | Random |
| Tonstad-2002a | -0.137 | [-0.204, -0.071] | 91.3 | 829.96 | 0.00 | Random |
| Tonstad-2002b | -0.121 | [-0.191, -0.051] | 92.3 | 929.88 | 0.00 | Random |
| Azadbakht-2003 | -0.116 | [-0.186, -0.047] | 92.2 | 921.47 | 0.00 | Random |
| Cuevas-2003 | -0.121 | [-0.191, -0.051] | 92.3 | 931.16 | 0.00 | Random |
| Desroches-2004a | -0.121 | [-0.191, -0.051] | 92.3 | 930.77 | 0.00 | Random |
| Desroches-2004b | -0.119 | [-0.189, -0.050] | 92.3 | 929.13 | 0.00 | Random |
| Chen-2005a | -0.106 | [-0.173, -0.038] | 91.7 | 868.53 | 0.00 | Random |
| Chen-2005b | -0.121 | [-0.190, -0.051] | 92.3 | 931.17 | 0.00 | Random |
| Xu-2005 | -0.119 | [-0.189, -0.050] | 92.3 | 930.04 | 0.00 | Random |
| Chen-2006 | -0.112 | [-0.181, -0.043] | 92.1 | 913.07 | 0.00 | Random |
| McVeigh-2006a | -0.120 | [-0.190, -0.049] | 92.2 | 919.64 | 0.00 | Random |
| McVeigh-2006b | -0.119 | [-0.189, -0.049] | 92.1 | 912.31 | 0.00 | Random |
| Liao-2007 | -0.120 | [-0.190, -0.050] | 92.3 | 930.34 | 0.00 | Random |
| Matthan-2007a | -0.121 | [-0.191, -0.051] | 92.3 | 930.52 | 0.00 | Random |
| Matthan-2007b | -0.122 | [-0.192, -0.052] | 92.3 | 931.04 | 0.00 | Random |
| Matthan-2007c | -0.121 | [-0.191, -0.051] | 92.3 | 930.11 | 0.00 | Random |
| Azadbakht-2008 | -0.112 | [-0.181, -0.043] | 92.1 | 913.64 | 0.00 | Random |
| Borodin-2009 | -0.118 | [-0.187, -0.048] | 92.2 | 925.43 | 0.00 | Random |
| Pipe-2009 | -0.121 | [-0.191, -0.051] | 92.3 | 931.02 | 0.00 | Random |
| Shidfar-2009 | -0.114 | [-0.183, -0.045] | 91.9 | 886.12 | 0.00 | Random |
| Beavers-2010 | -0.122 | [-0.191, -0.052] | 92.3 | 931.43 | 0.00 | Random |
| Campbell-2010 | -0.120 | [-0.190, -0.050] | 92.3 | 930.00 | 0.00 | Random |
| Jassi-2010 | -0.114 | [-0.183, -0.045] | 91.9 | 891.76 | 0.00 | Random |
| Santo-2010a | -0.124 | [-0.194, -0.055] | 92.3 | 929.93 | 0.00 | Random |
| Santo-2010b | -0.126 | [-0.196, -0.057] | 92.2 | 924.82 | 0.00 | Random |
| Tabibi-2010 | -0.122 | [-0.192, -0.053] | 92.3 | 931.23 | 0.00 | Random |
| Weisse-2010 | -0.121 | [-0.191, -0.052] | 92.3 | 931.43 | 0.00 | Random |
| Ma-2011 | -0.122 | [-0.192, -0.053] | 92.3 | 931.49 | 0.00 | Random |
| Liu-2012a | -0.120 | [-0.190, -0.050] | 92.2 | 928.22 | 0.00 | Random |
| Liu-2012b | -0.123 | [-0.193, -0.053] | 92.3 | 931.46 | 0.00 | Random |
| Sirtori-2012a | -0.117 | [-0.187, -0.048] | 92.2 | 925.34 | 0.00 | Random |
| Sirtori-2012b | -0.120 | [-0.190, -0.050] | 92.3 | 929.69 | 0.00 | Random |
| Bahr-2013 | -0.121 | [-0.191, -0.051] | 92.3 | 930.82 | 0.00 | Random |
| Liu-2014a | -0.118 | [-0.188, -0.048] | 92.2 | 925.53 | 0.00 | Random |
| Liu-2014b | -0.119 | [-0.189, -0.049] | 92.2 | 926.96 | 0.00 | Random |
| Bahr-2015 | -0.119 | [-0.189, -0.049] | 92.2 | 927.35 | 0.00 | Random |
| Frota-2015 | -0.113 | [-0.178, -0.049] | 90.2 | 738.09 | 0.00 | Random |
| Hill-2015a | -0.119 | [-0.189, -0.049] | 92.2 | 928.06 | 0.00 | Random |
| Hill-2015b | -0.124 | [-0.194, -0.054] | 92.3 | 931.00 | 0.00 | Random |
| Hill-2015c | -0.125 | [-0.195, -0.055] | 92.3 | 929.74 | 0.00 | Random |
| Hill-2015d | -0.123 | [-0.193, -0.053] | 92.3 | 931.44 | 0.00 | Random |
| Hill-2015e | -0.123 | [-0.193, -0.053] | 92.3 | 931.33 | 0.00 | Random |
| Hill-2015f | -0.121 | [-0.191, -0.052] | 92.3 | 931.13 | 0.00 | Random |
| Jamilian-2015 | -0.122 | [-0.192, -0.052] | 92.3 | 931.37 | 0.00 | Random |
| Padhi-2015 | -0.123 | [-0.194, -0.053] | 92.3 | 931.16 | 0.00 | Random |
| Tahavorgar-2015 | -0.127 | [-0.191, -0.063] | 86.5 | 533.96 | 0.00 | Random |
| Sucher-2017 | -0.125 | [-0.195, -0.056] | 92.2 | 928.11 | 0.00 | Random |
| Karamali-2018 | -0.123 | [-0.193, -0.054] | 92.3 | 931.22 | 0.00 | Random |
| Bergeron-2019a | -0.119 | [-0.189, -0.049] | 92.2 | 924.98 | 0.00 | Random |
| Bergeron-2019b | -0.120 | [-0.190, -0.050] | 92.2 | 926.13 | 0.00 | Random |
| Bergeron-2019c | -0.120 | [-0.190, -0.050] | 92.2 | 928.84 | 0.00 | Random |
| Bergeron-2019d | -0.120 | [-0.190, -0.050] | 92.2 | 927.71 | 0.00 | Random |
| Basciani-2020a | -0.122 | [-0.191, -0.052] | 92.3 | 931.24 | 0.00 | Random |
| Basciani-2020b | -0.117 | [-0.186, -0.047] | 92.2 | 920.33 | 0.00 | Random |
| George-2020 | -0.123 | [-0.192, -0.053] | 92.3 | 931.49 | 0.00 | Random |
| **Triglyceride (mmol/L)** | | | | | | |
| None | -0.051 | [-0.094, -0.008] | 74.5 | 270.70 | 0.00 | Random |
| Bosello-1988 | -0.047 | [-0.090, -0.004] | 74.4 | 265.58 | 0.00 | Random |
| Bakhit-1994a | -0.051 | [-0.094, -0.008] | 74.9 | 270.70 | 0.00 | Random |
| Bakhit-1994b | -0.053 | [-0.096, -0.010] | 74.8 | 269.80 | 0.00 | Random |
| Wang-1995 | -0.054 | [-0.097, -0.011] | 74.6 | 267.74 | 0.00 | Random |
| Kurowska-1997 | -0.053 | [-0.096, -0.010] | 74.7 | 268.83 | 0.00 | Random |
| Nilausen-1998 | -0.052 | [-0.096, -0.009] | 74.8 | 269.99 | 0.00 | Random |
| Shige-1998 | -0.052 | [-0.095, -0.009] | 74.9 | 270.54 | 0.00 | Random |
| Ashton-2000 | -0.050 | [-0.093, -0.007] | 74.8 | 270.35 | 0.00 | Random |
| Jenkins-2000 | -0.048 | [-0.091, -0.005] | 74.6 | 268.03 | 0.00 | Random |
| Teixeira-2000a | -0.053 | [-0.096, -0.010] | 74.8 | 269.71 | 0.00 | Random |
| Teixeira-2000b | -0.049 | [-0.092, -0.006] | 74.7 | 268.30 | 0.00 | Random |
| Vigna-2000 | -0.052 | [-0.095, -0.008] | 74.8 | 270.16 | 0.00 | Random |
| Gardner-2001a | -0.051 | [-0.094, -0.007] | 74.9 | 270.66 | 0.00 | Random |
| Gardner-2001b | -0.051 | [-0.094, -0.007] | 74.9 | 270.67 | 0.00 | Random |
| Teede-2001 | -0.049 | [-0.092, -0.005] | 74.7 | 268.33 | 0.00 | Random |
| Tonstad-2002a | -0.052 | [-0.096, -0.009] | 74.8 | 269.94 | 0.00 | Random |
| Tonstad-2002b | -0.050 | [-0.094, -0.007] | 74.9 | 270.64 | 0.00 | Random |
| Azadbakht-2003 | -0.049 | [-0.093, -0.005] | 74.5 | 267.00 | 0.00 | Random |
| Cuevas-2003 | -0.048 | [-0.091, -0.005] | 74.7 | 268.49 | 0.00 | Random |
| Desroches-2004a | -0.050 | [-0.094, -0.007] | 74.9 | 270.62 | 0.00 | Random |
| Desroches-2004b | -0.049 | [-0.092, -0.006] | 74.7 | 269.08 | 0.00 | Random |
| Chen-2005a | -0.049 | [-0.091, -0.006] | 74.2 | 263.15 | 0.00 | Random |
| Chen-2005b | -0.051 | [-0.094, -0.008] | 74.9 | 270.70 | 0.00 | Random |
| Chen-2006 | -0.050 | [-0.093, -0.007] | 74.8 | 270.34 | 0.00 | Random |
| McVeigh-2006a | -0.053 | [-0.097, -0.010] | 74.6 | 268.14 | 0.00 | Random |
| McVeigh-2006b | -0.052 | [-0.096, -0.009] | 74.8 | 270.05 | 0.00 | Random |
| Liao-2007 | -0.053 | [-0.096, -0.010] | 74.7 | 269.20 | 0.00 | Random |
| Matthan-2007a | -0.054 | [-0.097, -0.011] | 74.7 | 268.40 | 0.00 | Random |
| Matthan-2007b | -0.053 | [-0.096, -0.010] | 74.8 | 269.46 | 0.00 | Random |
| Matthan-2007c | -0.053 | [-0.096, -0.010] | 74.7 | 268.94 | 0.00 | Random |
| Azadbakht-2008 | -0.049 | [-0.092, -0.006] | 74.7 | 269.03 | 0.00 | Random |
| Borodin-2009 | -0.046 | [-0.089, -0.003] | 74.1 | 262.32 | 0.00 | Random |
| Pipe-2009 | -0.053 | [-0.097, -0.010] | 74.6 | 267.93 | 0.00 | Random |
| Shidfar-2009 | -0.050 | [-0.094, -0.007] | 74.9 | 270.50 | 0.00 | Random |
| Beavers-2010 | -0.052 | [-0.095, -0.009] | 74.9 | 270.42 | 0.00 | Random |
| Campbell-2010 | -0.052 | [-0.095, -0.008] | 74.9 | 270.57 | 0.00 | Random |
| Jassi-2010 | -0.042 | [-0.079, -0.004] | 63.5 | 186.11 | 0.00 | Random |
| Santo-2010a | -0.053 | [-0.096, -0.010] | 74.8 | 269.52 | 0.00 | Random |
| Santo-2010b | -0.052 | [-0.095, -0.009] | 74.8 | 270.07 | 0.00 | Random |
| Tabibi-2010 | -0.052 | [-0.095, -0.009] | 74.8 | 269.45 | 0.00 | Random |
| Weisse-2010 | -0.056 | [-0.098, -0.013] | 74.2 | 263.15 | 0.00 | Random |
| Ma-2011 | -0.049 | [-0.092, -0.006] | 74.8 | 269.50 | 0.00 | Random |
| Liu-2012a | -0.052 | [-0.095, -0.009] | 74.8 | 270.30 | 0.00 | Random |
| Liu-2012b | -0.053 | [-0.096, -0.010] | 74.7 | 269.21 | 0.00 | Random |
| Sirtori-2012a | -0.051 | [-0.094, -0.007] | 74.9 | 270.68 | 0.00 | Random |
| Sirtori-2012b | -0.051 | [-0.094, -0.008] | 74.9 | 270.70 | 0.00 | Random |
| Bahr-2013 | -0.052 | [-0.095, -0.009] | 74.8 | 270.27 | 0.00 | Random |
| Liu-2014a | -0.053 | [-0.096, -0.009] | 74.6 | 268.15 | 0.00 | Random |
| Liu-2014b | -0.050 | [-0.093, -0.006] | 74.8 | 269.85 | 0.00 | Random |
| Bahr-2015 | -0.051 | [-0.094, -0.008] | 74.9 | 270.67 | 0.00 | Random |
| Frota-2015 | -0.049 | [-0.096, -0.003] | 74.0 | 261.85 | 0.00 | Random |
| Hill-2015a | -0.052 | [-0.095, -0.009] | 74.9 | 270.39 | 0.00 | Random |
| Hill-2015b | -0.055 | [-0.098, -0.012] | 74.2 | 263.82 | 0.00 | Random |
| Hill-2015c | -0.050 | [-0.093, -0.007] | 74.9 | 270.54 | 0.00 | Random |
| Hill-2015d | -0.047 | [-0.090, -0.004] | 74.4 | 265.36 | 0.00 | Random |
| Hill-2015e | -0.057 | [-0.099, -0.014] | 73.8 | 259.89 | 0.00 | Random |
| Hill-2015f | -0.051 | [-0.094, -0.008] | 74.9 | 270.70 | 0.00 | Random |
| Jamilian-2015 | -0.048 | [-0.091, -0.005] | 74.7 | 268.30 | 0.00 | Random |
| Padhi-2015 | -0.053 | [-0.097, -0.010] | 74.6 | 267.30 | 0.00 | Random |
| Tahavorgar-2015 | -0.058 | [-0.100, -0.016] | 72.2 | 244.50 | 0.00 | Random |
| Sucher-2017 | -0.056 | [-0.098, -0.014] | 71.7 | 240.61 | 0.00 | Random |
| Karamali-2018 | -0.047 | [-0.090, -0.004] | 74.3 | 264.40 | 0.00 | Random |
| Bergeron-2019a | -0.053 | [-0.097, -0.010] | 74.6 | 267.38 | 0.00 | Random |
| Bergeron-2019b | -0.053 | [-0.096, -0.009] | 74.6 | 267.97 | 0.00 | Random |
| Bergeron-2019c | -0.052 | [-0.096, -0.008] | 74.6 | 267.85 | 0.00 | Random |
| Bergeron-2019d | -0.053 | [-0.097, -0.009] | 74.4 | 266.10 | 0.00 | Random |
| Basciani-2020a | -0.049 | [-0.092, -0.005] | 74.7 | 269.06 | 0.00 | Random |
| Basciani-2020b | -0.050 | [-0.093, -0.007] | 74.9 | 270.43 | 0.00 | Random |
| Crimarco-2020 | -0.052 | [-0.096, -0.009] | 74.8 | 269.86 | 0.00 | Random |
| George-2020 | -0.052 | [-0.096, -0.009] | 74.8 | 269.93 | 0.00 | Random |
| **LDL-cholesterol (mmol/L)** | | | | | | |
| None | -0.110 | [-0.183, -0.036] | 89.9 | 735.80 | 0.00 | Random |
| van Raaij-1981 | -0.109 | [-0.184, -0.034] | 90.0 | 733.58 | 0.00 | Random |
| van Raaij-1982a | -0.109 | [-0.183, -0.034] | 90.1 | 734.49 | 0.00 | Random |
| van Raaij-1982b | -0.113 | [-0.188, -0.039] | 90.0 | 733.20 | 0.00 | Random |
| Wiebe-1984 | -0.111 | [-0.185, -0.036] | 90.1 | 735.78 | 0.00 | Random |
| Bosello-1988 | -0.106 | [-0.180, -0.032] | 90.0 | 731.35 | 0.00 | Random |
| Bakhit-1994a | -0.108 | [-0.183, -0.034] | 90.1 | 734.17 | 0.00 | Random |
| Bakhit-1994b | -0.112 | [-0.187, -0.037] | 90.1 | 735.38 | 0.00 | Random |
| Wang-1995a | -0.111 | [-0.185, -0.036] | 90.1 | 735.78 | 0.00 | Random |
| Wang-1995b | -0.102 | [-0.176, -0.029] | 89.9 | 725.31 | 0.00 | Random |
| Kurowska-1997 | -0.110 | [-0.184, -0.035] | 90.1 | 735.65 | 0.00 | Random |
| Nilausen-1998 | -0.108 | [-0.182, -0.034] | 90.1 | 734.91 | 0.00 | Random |
| Shige-1998 | -0.111 | [-0.185, -0.037] | 90.1 | 735.74 | 0.00 | Random |
| Ashton-2000 | -0.110 | [-0.185, -0.035] | 90.1 | 735.57 | 0.00 | Random |
| Jenkins-2000 | -0.107 | [-0.181, -0.032] | 90.0 | 731.56 | 0.00 | Random |
| Vigna-2000 | -0.111 | [-0.185, -0.036] | 90.1 | 735.80 | 0.00 | Random |
| Gardner-2001a | -0.114 | [-0.189, -0.040] | 90.0 | 731.15 | 0.00 | Random |
| Gardner-2001b | -0.110 | [-0.185, -0.035] | 90.1 | 735.36 | 0.00 | Random |
| Teede-2001 | -0.109 | [-0.184, -0.034] | 90.1 | 734.70 | 0.00 | Random |
| Tonstad-2002a | -0.106 | [-0.181, -0.032] | 89.9 | 725.71 | 0.00 | Random |
| Tonstad-2002b | -0.109 | [-0.184, -0.034] | 90.0 | 733.29 | 0.00 | Random |
| Azadbakht-2003 | -0.108 | [-0.183, -0.033] | 90.0 | 730.06 | 0.00 | Random |
| Cuevas-2003 | -0.111 | [-0.185, -0.036] | 90.1 | 735.79 | 0.00 | Random |
| Bricarello-2004 | -0.107 | [-0.182, -0.033] | 90.0 | 730.43 | 0.00 | Random |
| Desroches-2004a | -0.111 | [-0.186, -0.037] | 90.1 | 735.74 | 0.00 | Random |
| Desroches-2004b | -0.110 | [-0.184, -0.035] | 90.1 | 735.62 | 0.00 | Random |
| Chen-2005a | -0.103 | [-0.177, -0.029] | 89.9 | 725.87 | 0.00 | Random |
| Chen-2005b | -0.111 | [-0.186, -0.037] | 90.1 | 735.71 | 0.00 | Random |
| Chen-2006 | -0.104 | [-0.178, -0.030] | 90.0 | 729.93 | 0.00 | Random |
| McVeigh-2006a | -0.108 | [-0.183, -0.032] | 89.9 | 721.89 | 0.00 | Random |
| McVeigh-2006b | -0.108 | [-0.183, -0.032] | 89.9 | 721.89 | 0.00 | Random |
| Gardner-2007a | -0.108 | [-0.182, -0.034] | 90.1 | 734.14 | 0.00 | Random |
| Gardner-2007b | -0.108 | [-0.183, -0.034] | 90.1 | 734.51 | 0.00 | Random |
| Liao-2007 | -0.109 | [-0.184, -0.034] | 90.1 | 734.67 | 0.00 | Random |
| Matthan-2007a | -0.110 | [-0.185, -0.035] | 90.1 | 735.51 | 0.00 | Random |
| Matthan-2007b | -0.111 | [-0.186, -0.037] | 90.1 | 735.64 | 0.00 | Random |
| Matthan-2007c | -0.109 | [-0.184, -0.034] | 90.1 | 734.20 | 0.00 | Random |
| Azadbakht-2008 | -0.101 | [-0.174, -0.028] | 89.7 | 709.32 | 0.00 | Random |
| Pipe-2009 | -0.110 | [-0.185, -0.035] | 90.1 | 735.60 | 0.00 | Random |
| Shidfar-2009 | -0.105 | [-0.178, -0.031] | 89.7 | 708.58 | 0.00 | Random |
| Beavers-2010 | -0.110 | [-0.185, -0.036] | 90.1 | 735.79 | 0.00 | Random |
| Campbell-2010 | -0.110 | [-0.185, -0.035] | 90.1 | 735.70 | 0.00 | Random |
| Jassi-2010 | -0.104 | [-0.177, -0.030] | 89.8 | 716.44 | 0.00 | Random |
| Santo-2010a | -0.110 | [-0.184, -0.036] | 90.1 | 735.80 | 0.00 | Random |
| Santo-2010b | -0.112 | [-0.186, -0.038] | 90.1 | 735.19 | 0.00 | Random |
| Tabibi-2010 | -0.111 | [-0.186, -0.037] | 90.1 | 735.71 | 0.00 | Random |
| Weisse-2010 | -0.109 | [-0.183, -0.035] | 90.1 | 735.25 | 0.00 | Random |
| Ma-2011 | -0.113 | [-0.188, -0.039] | 90.1 | 734.40 | 0.00 | Random |
| Liu-2012a | -0.108 | [-0.183, -0.034] | 90.0 | 733.30 | 0.00 | Random |
| Liu-2012b | -0.111 | [-0.186, -0.037] | 90.1 | 735.62 | 0.00 | Random |
| Sirtori-2012a | -0.108 | [-0.182, -0.033] | 90.1 | 734.16 | 0.00 | Random |
| Sirtori-2012b | -0.112 | [-0.187, -0.038] | 90.1 | 735.20 | 0.00 | Random |
| Bahr-2013 | -0.111 | [-0.186, -0.036] | 90.1 | 735.77 | 0.00 | Random |
| Liu-2014a | -0.107 | [-0.181, -0.032] | 90.0 | 729.07 | 0.00 | Random |
| Liu-2014b | -0.108 | [-0.182, -0.033] | 90.0 | 731.69 | 0.00 | Random |
| Bahr-2015 | -0.109 | [-0.183, -0.034] | 90.1 | 734.77 | 0.00 | Random |
| Frota-2015 | -0.100 | [-0.166, -0.034] | 86.4 | 537.93 | 0.00 | Random |
| Hill-2015a | -0.109 | [-0.183, -0.036] | 90.1 | 735.76 | 0.00 | Random |
| Hill-2015b | -0.110 | [-0.184, -0.036] | 90.1 | 735.79 | 0.00 | Random |
| Hill-2015c | -0.115 | [-0.189, -0.040] | 90.0 | 731.72 | 0.00 | Random |
| Hill-2015d | -0.112 | [-0.187, -0.037] | 90.1 | 735.23 | 0.00 | Random |
| Hill-2015e | -0.114 | [-0.188, -0.039] | 90.0 | 733.26 | 0.00 | Random |
| Hill-2015f | -0.113 | [-0.188, -0.039] | 90.1 | 733.81 | 0.00 | Random |
| Jamilian-2015 | -0.112 | [-0.187, -0.037] | 90.1 | 735.43 | 0.00 | Random |
| Padhi-2015 | -0.112 | [-0.188, -0.037] | 90.1 | 734.25 | 0.00 | Random |
| Tahavorgar-2015 | -0.116 | [-0.175, -0.058] | 80.1 | 367.28 | 0.00 | Random |
| Sucher-2017 | -0.114 | [-0.189, -0.039] | 89.9 | 725.63 | 0.00 | Random |
| Karamali-2018 | -0.114 | [-0.189, -0.040] | 90.0 | 732.54 | 0.00 | Random |
| Bergeron-2019a | -0.114 | [-0.189, -0.040] | 90.0 | 729.54 | 0.00 | Random |
| Bergeron-2019b | -0.114 | [-0.189, -0.039] | 90.0 | 730.78 | 0.00 | Random |
| Bergeron-2019c | -0.113 | [-0.188, -0.039] | 90.0 | 731.95 | 0.00 | Random |
| Bergeron-2019d | -0.114 | [-0.189, -0.039] | 90.0 | 730.58 | 0.00 | Random |
| Basciani-2020a | -0.109 | [-0.183, -0.034] | 90.1 | 734.25 | 0.00 | Random |
| Basciani-2020b | -0.106 | [-0.180, -0.032] | 89.9 | 725.35 | 0.00 | Random |
| Crimarco-2020 | -0.107 | [-0.182, -0.033] | 90.0 | 732.23 | 0.00 | Random |
| George-2020 | -0.113 | [-0.188, -0.039] | 90.1 | 733.74 | 0.00 | Random |
| **HDL-cholesterol (mmol/L)** | | | | | | |
| None | 0.029 | [0.015, 0.043] | 77.6 | 330.61 | 0.00 | Random |
| van Raaij-1981 | 0.028 | [0.014, 0.043] | 77.9 | 329.84 | 0.00 | Random |
| van Raaij-1982a | 0.028 | [0.014, 0.042] | 77.8 | 328.10 | 0.00 | Random |
| van Raaij-1982b | 0.029 | [0.015, 0.043] | 77.9 | 330.43 | 0.00 | Random |
| Bosello-1988 | 0.028 | [0.014, 0.043] | 77.9 | 329.77 | 0.00 | Random |
| Bakhit-1994a | 0.029 | [0.015, 0.043] | 77.9 | 330.56 | 0.00 | Random |
| Bakhit-1994b | 0.029 | [0.015, 0.043] | 77.9 | 330.60 | 0.00 | Random |
| Wang-1995a | 0.029 | [0.015, 0.043] | 77.9 | 329.75 | 0.00 | Random |
| Wang-1995b | 0.029 | [0.015, 0.043] | 77.7 | 328.06 | 0.00 | Random |
| Kurowska-1997 | 0.028 | [0.014, 0.042] | 77.8 | 329.57 | 0.00 | Random |
| Nilausen-1998 | 0.029 | [0.015, 0.043] | 77.9 | 330.10 | 0.00 | Random |
| Shige-1998 | 0.029 | [0.015, 0.043] | 77.9 | 330.45 | 0.00 | Random |
| Ashton-2000 | 0.031 | [0.017, 0.045] | 77.0 | 317.06 | 0.00 | Random |
| Jenkins-2000 | 0.030 | [0.016, 0.044] | 77.8 | 329.13 | 0.00 | Random |
| Teixeira-2000a | 0.029 | [0.015, 0.043] | 77.9 | 330.50 | 0.00 | Random |
| Teixeira-2000b | 0.029 | [0.015, 0.044] | 77.9 | 330.20 | 0.00 | Random |
| Vigna-2000 | 0.031 | [0.014, 0.048] | 77.4 | 322.57 | 0.00 | Random |
| Gardner-2001a | 0.028 | [0.014, 0.043] | 77.9 | 329.66 | 0.00 | Random |
| Gardner-2001b | 0.028 | [0.014, 0.043] | 77.9 | 329.71 | 0.00 | Random |
| Teede-2001 | 0.029 | [0.014, 0.043] | 77.9 | 330.34 | 0.00 | Random |
| Tonstad-2002a | 0.029 | [0.015, 0.043] | 77.9 | 330.57 | 0.00 | Random |
| Tonstad-2002b | 0.029 | [0.015, 0.043] | 77.9 | 330.60 | 0.00 | Random |
| Azadbakht-2003 | 0.029 | [0.015, 0.043] | 77.9 | 330.59 | 0.00 | Random |
| Cuevas-2003 | 0.029 | [0.014, 0.043] | 77.8 | 328.55 | 0.00 | Random |
| Bricarello-2004 | 0.028 | [0.014, 0.042] | 77.7 | 327.38 | 0.00 | Random |
| Desroches-2004a | 0.028 | [0.014, 0.042] | 77.8 | 329.03 | 0.00 | Random |
| Desroches-2004b | 0.028 | [0.014, 0.042] | 77.8 | 329.03 | 0.00 | Random |
| Chen-2005a | 0.029 | [0.015, 0.043] | 77.9 | 330.54 | 0.00 | Random |
| Chen-2005b | 0.029 | [0.015, 0.043] | 77.9 | 330.61 | 0.00 | Random |
| Chen-2006 | 0.030 | [0.015, 0.044] | 77.9 | 329.81 | 0.00 | Random |
| McVeigh-2006a | 0.028 | [0.014, 0.042] | 77.8 | 328.56 | 0.00 | Random |
| McVeigh-2006b | 0.029 | [0.015, 0.043] | 77.9 | 330.57 | 0.00 | Random |
| Liao-2007 | 0.029 | [0.015, 0.043] | 77.9 | 330.59 | 0.00 | Random |
| Matthan-2007a | 0.030 | [0.016, 0.044] | 77.7 | 327.55 | 0.00 | Random |
| Matthan-2007b | 0.030 | [0.016, 0.044] | 77.7 | 326.67 | 0.00 | Random |
| Matthan-2007c | 0.029 | [0.015, 0.044] | 77.9 | 330.27 | 0.00 | Random |
| Azadbakht-2008 | 0.029 | [0.015, 0.043] | 77.9 | 330.61 | 0.00 | Random |
| Borodin-2009 | 0.027 | [0.013, 0.041] | 77.3 | 321.78 | 0.00 | Random |
| Pipe-2009 | 0.030 | [0.016, 0.044] | 77.8 | 329.17 | 0.00 | Random |
| Shidfar-2009 | 0.028 | [0.014, 0.042] | 77.8 | 328.21 | 0.00 | Random |
| Beavers-2010 | 0.030 | [0.015, 0.044] | 77.9 | 329.62 | 0.00 | Random |
| Campbell-2010 | 0.030 | [0.016, 0.044] | 77.8 | 329.04 | 0.00 | Random |
| Jassi-2010 | 0.026 | [0.012, 0.040] | 76.8 | 314.60 | 0.00 | Random |
| Santo-2010a | 0.028 | [0.014, 0.042] | 77.5 | 324.22 | 0.00 | Random |
| Santo-2010b | 0.028 | [0.014, 0.041] | 76.5 | 309.99 | 0.00 | Random |
| Tabibi-2010 | 0.030 | [0.016, 0.044] | 77.7 | 327.81 | 0.00 | Random |
| Weisse-2010 | 0.028 | [0.014, 0.043] | 77.9 | 329.96 | 0.00 | Random |
| Ma-2011 | 0.029 | [0.015, 0.043] | 77.9 | 330.59 | 0.00 | Random |
| Liu-2012a | 0.029 | [0.015, 0.043] | 77.9 | 330.50 | 0.00 | Random |
| Liu-2012b | 0.029 | [0.015, 0.043] | 77.9 | 330.59 | 0.00 | Random |
| Sirtori-2012a | 0.030 | [0.016, 0.044] | 77.8 | 328.78 | 0.00 | Random |
| Sirtori-2012b | 0.029 | [0.015, 0.043] | 77.9 | 330.52 | 0.00 | Random |
| Bahr-2013 | 0.030 | [0.016, 0.044] | 77.7 | 327.22 | 0.00 | Random |
| Liu-2014a | 0.029 | [0.015, 0.043] | 77.9 | 330.31 | 0.00 | Random |
| Liu-2014b | 0.030 | [0.015, 0.044] | 77.8 | 329.47 | 0.00 | Random |
| Bahr-2015 | 0.030 | [0.016, 0.044] | 77.8 | 328.66 | 0.00 | Random |
| Frota-2015 | 0.027 | [0.013, 0.042] | 70.0 | 243.10 | 0.00 | Random |
| Hill-2015a | 0.029 | [0.015, 0.044] | 77.9 | 329.86 | 0.00 | Random |
| Hill-2015b | 0.029 | [0.014, 0.043] | 77.9 | 330.46 | 0.00 | Random |
| Hill-2015c | 0.030 | [0.016, 0.044] | 77.6 | 326.45 | 0.00 | Random |
| Hill-2015d | 0.029 | [0.015, 0.043] | 77.9 | 330.40 | 0.00 | Random |
| Hill-2015e | 0.030 | [0.016, 0.044] | 77.7 | 327.39 | 0.00 | Random |
| Hill-2015f | 0.030 | [0.016, 0.044] | 77.8 | 328.40 | 0.00 | Random |
| Jamilian-2015 | 0.029 | [0.015, 0.043] | 77.9 | 330.42 | 0.00 | Random |
| Padhi-2015 | 0.029 | [0.015, 0.044] | 77.8 | 329.47 | 0.00 | Random |
| Tahavorgar-2015 | 0.032 | [0.019, 0.046] | 73.2 | 272.30 | 0.00 | Random |
| Sucher-2017 | 0.029 | [0.015, 0.044] | 77.8 | 328.69 | 0.00 | Random |
| Karamali-2018 | 0.030 | [0.016, 0.044] | 77.8 | 328.58 | 0.00 | Random |
| Bergeron-2019a | 0.030 | [0.016, 0.045] | 77.5 | 324.00 | 0.00 | Random |
| Bergeron-2019b | 0.030 | [0.016, 0.045] | 77.5 | 324.00 | 0.00 | Random |
| Bergeron-2019c | 0.030 | [0.015, 0.044] | 77.9 | 329.84 | 0.00 | Random |
| Bergeron-2019d | 0.030 | [0.016, 0.044] | 77.8 | 329.01 | 0.00 | Random |
| Basciani-2020a | 0.032 | [0.018, 0.046] | 75.0 | 292.22 | 0.00 | Random |
| Basciani-2020b | 0.030 | [0.016, 0.044] | 77.3 | 321.38 | 0.00 | Random |
| Crimarco-2020 | 0.029 | [0.015, 0.043] | 77.9 | 330.39 | 0.00 | Random |
| George-2020 | 0.029 | [0.015, 0.043] | 77.9 | 330.61 | 0.00 | Random |
| **CRP (mg/L)** | | | | | | |
| None | -0.021 | [-0.174, 0.132] | 65.4 | 69.29 | 0.00 | Random |
| McVeigh-2006a | -0.035 | [-0.224, 0.154] | 65.9 | 67.37 | 0.00 | Random |
| McVeigh-2006b | -0.026 | [-0.209, 0.157] | 65.6 | 66.86 | 0.00 | Random |
| Matthan-2007a | -0.021 | [-0.175, 0.132] | 66.4 | 68.53 | 0.00 | Random |
| Matthan-2007b | -0.022 | [-0.176, 0.132] | 66.8 | 69.29 | 0.00 | Random |
| Matthan-2007c | -0.021 | [-0.175, 0.133] | 66.6 | 68.93 | 0.00 | Random |
| Ma-2011 | -0.005 | [-0.158, 0.148] | 65.5 | 66.63 | 0.00 | Random |
| Liu-2012a | -0.018 | [-0.177, 0.140] | 66.7 | 69.17 | 0.00 | Random |
| Liu-2012b | -0.024 | [-0.181, 0.133] | 66.8 | 69.28 | 0.00 | Random |
| Sirtori-2012a | -0.022 | [-0.177, 0.133] | 66.8 | 69.29 | 0.00 | Random |
| Sirtori-2012b | -0.023 | [-0.181, 0.135] | 66.8 | 69.29 | 0.00 | Random |
| Bahr-2013 | -0.025 | [-0.183, 0.133] | 66.8 | 69.28 | 0.00 | Random |
| Liu-2014a | -0.011 | [-0.143, 0.164] | 64.5 | 64.78 | 0.00 | Random |
| Liu-2014b | -0.014 | [-0.170, 0.143] | 66.6 | 68.81 | 0.00 | Random |
| Hill-2015a | -0.004 | [-0.155, 0.147] | 64.8 | 65.29 | 0.00 | Random |
| Hill-2015b | -0.010 | [-0.163, 0.143] | 65.9 | 67.49 | 0.00 | Random |
| Hill-2015c | -0.024 | [-0.179, 0.130] | 66.7 | 69.16 | 0.00 | Random |
| Hill-2015d | -0.022 | [-0.177, 0.133] | 66.8 | 69.29 | 0.00 | Random |
| Hill-2015e | -0.024 | [-0.178, 0.131] | 66.8 | 69.23 | 0.00 | Random |
| Hill-2015f | -0.022 | [-0.177, 0.133] | 66.8 | 69.29 | 0.00 | Random |
| Jamilian-2015 | -0.008 | [-0.160, 0.145] | 65.6 | 66.80 | 0.00 | Random |
| Padhi-2015 | -0.081 | [-0.303, 0.142] | 66.8 | 69.19 | 0.00 | Random |
| Sucher-2017 | -0.066 | [-0.148, 0.016] | 14.9 | 27.01 | 0.26 | Random |
| Karamali-2018 | -0.011 | [-0.167, 0.145] | 66.4 | 68.48 | 0.00 | Random |
| Basciani-2020a | -0.043 | [-0.195, 0.109] | 64.5 | 64.85 | 0.00 | Random |
| Basciani-2020b | -0.020 | [-0.178, 0.137] | 66.8 | 69.25 | 0.00 | Random |

**Supplementary data**

# **Supplementary Table 6.** Sensitivity analysis following the removal of single groups or randomized controlled trials to assess the robustness of network meta-analyses results for HDL-C

| **Group excluded** | **Weighted mean difference** | **[95 % CI]** | **I^2^ (%)** | **χ^2^** | ***P* for χ^2^** | **Model** |
| --- | --- | --- | --- | --- | --- | --- |
| None | -0.009 | [-0.027, 0.009] | 92.1 | 661.09 | 0.00 | Random |
| Jenkins-2000 | -0.007 | [-0.025, 0.011] | 92.2 | 656.56 | 0.00 | Random |
| Farnsworth-2003 | -0.009 | [-0.027, 0.009] | 92.3 | 661.09 | 0.00 | Random |
| Brinkworth-2004 | -0.009 | [-0.028, 0.009] | 92.3 | 661.04 | 0.00 | Random |
| Mamo-2005 | -0.009 | [-0.027, 0.009] | 92.3 | 660.97 | 0.00 | Random |
| Noakes-2005 | -0.009 | [-0.027, 0.009] | 92.3 | 661.02 | 0.00 | Random |
| Stephenson-2005 | -0.008 | [-0.026, 0.010] | 92.3 | 659.42 | 0.00 | Random |
| Ferrara-2006 | -0.009 | [-0.027, 0.009] | 92.2 | 657.96 | 0.00 | Random |
| Hodgson-2006 | -0.009 | [-0.027, 0.009] | 92.3 | 661.08 | 0.00 | Random |
| McAuley-2006 | -0.009 | [-0.027, 0.009] | 92.3 | 661.08 | 0.00 | Random |
| Treyzon-2008 | -0.008 | [-0.026, 0.010] | 92.3 | 659.36 | 0.00 | Random |
| Delbridge-2009 | -0.009 | [-0.027, 0.009] | 92.3 | 661.02 | 0.00 | Random |
| Papakonstantinou-2010 | -0.006 | [-0.022, 0.010] | 86.9 | 389.35 | 0.00 | Random |
| Gögebakan-2011 | -0.008 | [-0.027, 0.010] | 92.3 | 659.73 | 0.00 | Random |
| Toscani-2011a | -0.005 | [-0.023, 0.013] | 91.9 | 631.04 | 0.00 | Random |
| Toscani-2011b | -0.008 | [-0.027, 0.010] | 92.3 | 660.92 | 0.00 | Random |
| Mehrabani-2012 | -0.009 | [-0.028, 0.009] | 92.3 | 660.93 | 0.00 | Random |
| Sheikholeslami-2012 | -0.009 | [-0.028, 0.009] | 92.3 | 660.63 | 0.00 | Random |
| Azadbakht-2013 | -0.008 | [-0.032, 0.015] | 92.3 | 660.95 | 0.00 | Random |
| Dalle Grave-2013 | -0.008 | [-0.026, 0.010] | 92.3 | 660.71 | 0.00 | Random |
| Tang-2013 | -0.009 | [-0.027, 0.009] | 92.3 | 661.05 | 0.00 | Random |
| Machin-2014 | -0.008 | [-0.026, 0.010] | 92.3 | 658.69 | 0.00 | Random |
| Pedersen-2014 | -0.009 | [-0.027, 0.009] | 92.3 | 661.06 | 0.00 | Random |
| Gulati-2017 | -0.008 | [-0.026, 0.011] | 92.2 | 655.79 | 0.00 | Random |
| Fernandes-2018 | -0.009 | [-0.027, 0.009] | 92.3 | 661.06 | 0.00 | Random |
| Porter-2019 | -0.009 | [-0.028, 0.009] | 92.3 | 660.70 | 0.00 | Random |
| Bray-2020 | -0.01 | [-0.028, 0.008] | 92.3 | 658.47 | 0.00 | Random |
| Hudson-2020 | -0.009 | [-0.027, 0.009] | 92.3 | 661.06 | 0.00 | Random |
| Tischmann-2020 | -0.009 | [-0.027, 0.009] | 92.3 | 661.06 | 0.00 | Random |
| Yilmaz-2021 | -0.012 | [-0.028, 0.005] | 87.2 | 398.11 | 0.00 | Random |
| Meckling-2007a | -0.009 | [-0.028, 0.009] | 92.3 | 660.49 | 0.00 | Random |
| Meckling-2007b | -0.010 | [-0.028, 0.008] | 92.2 | 650.35 | 0.00 | Random |
| Claessens-2009a | -0.010 | [-0.028, 0.008] | 92.2 | 657.42 | 0.00 | Random |
| Claessens-2009b | -0.010 | [-0.028, 0.008] | 92.3 | 659.80 | 0.00 | Random |
| Sacks-2009a | -0.009 | [-0.028, 0.009] | 92.3 | 660.35 | 0.00 | Random |
| Sacks-2009b | -0.011 | [-0.029, 0.008] | 92.2 | 651.59 | 0.00 | Random |
| Roussell-2012a | -0.009 | [-0.027, 0.009] | 92.3 | 661.01 | 0.00 | Random |
| Roussell-2012b | -0.009 | [-0.027, 0.009] | 92.3 | 661.06 | 0.00 | Random |
| Juraschek-2013a | -0.007 | [-0.026, 0.011] | 92.1 | 648.16 | 0.00 | Random |
| Juraschek-2013b | -0.008 | [-0.026, 0.010] | 92.2 | 657.41 | 0.00 | Random |
| Chiu-2014a | -0.008 | [-0.026, 0.010] | 92.3 | 659.33 | 0.00 | Random |
| Chiu-2014b | -0.009 | [-0.027, 0.010] | 92.3 | 660.71 | 0.00 | Random |
| de Luis-2015a | -0.010 | [-0.029, 0.008] | 92.2 | 655.83 | 0.00 | Random |
| de Luis-2015b | -0.010 | [-0.028, 0.008] | 92.3 | 659.75 | 0.00 | Random |
| Hill-2015a | -0.007 | [-0.026, 0.011] | 92.2 | 654.85 | 0.00 | Random |
| Hill-2015b | -0.008 | [-0.026, 0.010] | 92.3 | 658.10 | 0.00 | Random |
| Hill-2015c | -0.008 | [-0.026, 0.010] | 92.3 | 659.14 | 0.00 | Random |
| Hill-2015d | -0.008 | [-0.026, 0.010] | 92.2 | 656.92 | 0.00 | Random |
| Hill-2015e | -0.009 | [-0.027, 0.009] | 92.3 | 661.08 | 0.00 | Random |
| Hill-2015f | -0.009 | [-0.027, 0.010] | 92.3 | 660.75 | 0.00 | Random |
| Fekete-2016a | -0.008 | [-0.026, 0.010] | 92.2 | 656.57 | 0.00 | Random |
| Fekete-2016b | -0.008 | [-0.026, 0.011] | 92.2 | 656.53 | 0.00 | Random |
| Mateo-Gallego-2017a | -0.005 | [-0.023, 0.013] | 91.9 | 627.59 | 0.00 | Random |
| Mateo-Gallego-2017b | -0.009 | [-0.028, 0.009] | 92.3 | 659.68 | 0.00 | Random |

**Supplementary data**

# **Supplementary Table 7.** Meta-regression results

| Primary outcome | No. of trails or groups | Meta-regression coefficient (95% CI) | *P* |
| --- | --- | --- | --- |
| SBP | 84 | -0.192 (-0.344 to -0.041) | **0.013** |
| DBP | 84 | -0.096 (-0.182 to -0.008) | **0.032** |
| TC | 102 | -0.010 (-0.022 to 0.002) | 0.101 |
| TG | 96 | -0.008 (-0.016 to 0.001) | 0.072 |
| LDL-C | 96 | -0.005 (-0.016 to 0.006) | 0.382 |
| HDL-C | 102 | -0.001 (-0.004 to 0.003) | 0.751 |

^1^*P* < 0.05 was considered to indicate a significant correlation between protein quantity and primary outcome.

SBP, systolic blood pressure; DBP, diastolic blood pressure; TC, total cholesterol; TG, triglyceride; LDL-C, low-density-lipoprotein-cholesterol; HDL-C, high-density-lipoprotein-cholesterol.

**Supplementary data**

# Supplementary Table 8. League table for SBP

| LMH | -0.39  (-5.99, 5.21) | 1.91  (-2.98, 6.80) | -1.89  (-8.02, 4.23) | -0.77  (-5.35, 3.81) | -2.08  (-7.94, 3.78) | -0.96  (-7.08, 5.17) |
| --- | --- | --- | --- | --- | --- | --- |
|  | LHL | 2.30  (-1.00, 5.59) | -1.50  (-3.99, 0.98) | -0.38  (-3.64, 2.88) | -1.69  (-4.56, 1.18) | -0.57  (-3.90, 2.76) |
|  |  | LHH | -3.80  (-7.92, 0.32) | **-2.68**  **(-4.42, -0.93)** | **-3.99**  **(-7.58, -0.40)** | -2.86  (-6.90, 1.17) |
|  |  |  | HML | 1.12  (-2.97, 5.22) | -0.19  (-3.99, 3.62) | 0.94  (-3.22, 5.09) |
|  |  |  |  | HMH | -1.31  (-4.99, 2.37) | -0.19  (-4.29, 3.92) |
|  |  |  |  |  | HHL | 1.12  (-3.08, 5.33) |
|  |  |  |  |  |  | HHH |

^1^ The value above the treatments correspond to the difference in mean in SBP (mmHg) between the column and the row.

HHH, high-protein, high-carbohydrate, high-fat diet; HHL, high-protein, high-carbohydrate, low-fat diet; HMH, high-protein, moderate-carbohydrate, high-fat diet; HML, high-protein, moderate-carbohydrate, low-fat diet; LHH, low-protein, high-carbohydrate, high-fat diet; LHL, low-protein, high-carbohydrate, low-fat diet; LMH, low-protein, moderate-carbohydrate, high-fat diet; SBP, systolic blood pressure.

**Supplementary data**

# Supplementary Table 9. League table for DBP

| LMH | 1.11  (-2.99, 5.21) | 1.39  (-2.14, 4.91) | 0.78  (-3.63, 5.19) | -0.37  (-3.67, 2.92) | -0.41  (-4.73, 3.92) | 0.67  (-3.73, 5.08) |
| --- | --- | --- | --- | --- | --- | --- |
|  | LHL | 0.28  (-2.19, 2.74) | -0.33  (-2.07, 1.41) | -1.49  (-3.96, 0.99) | -1.52  (-3.49, 0.45) | -0.44  (-2.77, 1.90) |
|  |  | LHH | -0.61  (-3.54, 2.33) | **-1.76**  **(-3.03, -0.50)** | -1.80  (-4.57, 0.98) | -0.71  (-3.59, 2.16) |
|  |  |  | HML | -1.15  (-4.11, 1.80) | -1.19  (-3.80, 1.43) | -0.11  (-2.99, 2.78) |
|  |  |  |  | HMH | -0.03  (-2.87, 2.80) | 1.05  (-1.91, 4.00) |
|  |  |  |  |  | HHL | 1.08  (-1.87, 4.03) |
|  |  |  |  |  |  | HHH |

^1^ The value above the treatments correspond to the difference in mean in DBP (mmHg) between the column and the row.
DBP, diastolic blood pressure; HHH, high-protein, high-carbohydrate, high-fat diet; HHL, high-protein, high-carbohydrate, low-fat diet; HMH, high-protein, moderate-carbohydrate, high-fat diet; HML, high-protein, moderate-carbohydrate, low-fat diet; LHH, low-protein, high-carbohydrate, high-fat diet; LHL, low-protein, high-carbohydrate, low-fat diet; LMH, low-protein, moderate-carbohydrate, high-fat diet.

**Supplementary data**

# Supplementary Table 10. League table for TC

| LMH | 0.04  (-0.18, 0.27) | 0.11  (-0.08, 0.30) | 0.01  (-0.23, 0.25) | -0.04  (-0.20, 0.12) | -0.10  (-0.35, 0.15) | 0.01  (-0.25, 0.27) |
| --- | --- | --- | --- | --- | --- | --- |
|  | LHL | 0.06  (-0.08, 0.21) | -0.03  (-0.14, 0.07) | -0.08  (-0.24, 0.07) | -0.14  (-0.28, 0.00) | -0.03  (-0.19, 0.13) |
|  |  | LHH | -0.10  (-0.26, 0.07) | **-0.15**  **(-0.24, -0.05)** | **-0.21**  **(-0.38, -0.03)** | -0.09  (-0.29, 0.10) |
|  |  |  | HML | -0.05  (-0.22, 0.13) | -0.11  (-0.28, 0.07) | 0.00  (-0.19, 0.20) |
|  |  |  |  | HMH | -0.06  (-0.24, 0.13) | 0.05  (-0.15, 0.26) |
|  |  |  |  |  | HHL | 0.11  (-0.10, 0.32) |
|  |  |  |  |  |  | HHH |

^1^ The value above the treatments correspond to the difference in mean in TC (mmol/L) between the column and the row.
TC, total cholesterol; LMH, low-protein, moderate-carbohydrate, high-fat diet; LHL, low-protein, high-carbohydrate, low-fat diet; LHH, low-protein, high-carbohydrate, high-fat diet; HML, high-protein, moderate-carbohydrate, low-fat diet; HMH, high-protein, moderate-carbohydrate, high-fat diet; HHL, high-protein, high-carbohydrate, low-fat diet; HHH, high-protein, high-carbohydrate, high-fat diet.

**Supplementary data**

# Supplementary Table 11. League table for TG

| LMH | -0.01  (-0.31, 0.29) | 0.14  (-0.11, 0.39) | -0.08  (-0.39, 0.23) | -0.03  (-0.25, 0.19) | 0.11  (-0.21, 0.43) | 0.02  (-0.33, 0.36) |
| --- | --- | --- | --- | --- | --- | --- |
|  | LHL | 0.15  (-0.04, 0.34) | -0.07  (-0.20, 0.06) | -0.02  (-0.22, 0.18) | 0.12  (-0.05, 0.30) | 0.03  (-0.17, 0.23) |
|  |  | LHH | **-0.22**  **(-0.43, -0.02)** | **-0.17**  **(-0.30, -0.04)** | -0.03  (-0.25, 0.19) | -0.12  (-0.37, 0.13) |
|  |  |  | HML | 0.05  (-0.17, 0.28) | 0.20  (-0.02, 0.41) | 0.10  (-0.13, 0.34) |
|  |  |  |  | HMH | 0.14  (-0.10, 0.38) | 0.05  (-0.22, 0.31) |
|  |  |  |  |  | HHL | -0.09  (-0.36, 0.17) |
|  |  |  |  |  |  | HHH |

^1^ The value above the treatments correspond to the difference in mean in TG (mmol/L) between the column and the row.
TG, triglyceride; LMH, low-protein, moderate-carbohydrate, high-fat diet; LHL, low-protein, high-carbohydrate, low-fat diet; LHH, low-protein, high-carbohydrate, high-fat diet; HML, high-protein, moderate-carbohydrate, low-fat diet; HMH, high-protein, moderate-carbohydrate, high-fat diet; HHL, high-protein, high-carbohydrate, low-fat diet; HHH, high-protein, high-carbohydrate, high-fat diet.

**Supplementary data**

# Supplementary Table 12. League table for LDL-C

| LMH | 0.02  (-0.18, 0.22) | 0.08  (-0.09, 0.25) | -0.01  (-0.22, 0.21) | -0.01  (-0.15, 0.13) | -0.10  (-0.33, 0.12) | 0.07  (-0.16, 0.30) |
| --- | --- | --- | --- | --- | --- | --- |
|  | LHL | 0.06  (-0.08, 0.20) | -0.03  (-0.13, 0.08) | -0.03  (-0.17, 0.11) | -0.12  (-0.27, 0.02) | 0.05  (-0.10, 0.19) |
|  |  | LHH | -0.09  (-0.25, 0.07) | -0.09  (-0.18, 0.00) | **-0.19**  **(-0.35, -0.02)** | -0.01  (-0.19, 0.16) |
|  |  |  | HML | 0.00  (-0.17, 0.16) | -0.10  (-0.27, 0.07) | 0.07  (-0.10, 0.25) |
|  |  |  |  | HMH | -0.10  (-0.27, 0.08) | 0.08  (-0.11, 0.26) |
|  |  |  |  |  | HHL | 0.17  (-0.02, 0.37) |
|  |  |  |  |  |  | HHH |

^1^ The value above the treatments correspond to the difference in mean in LDL-C (mmol/L) between the column and the row.
LDL-C, low-density-lipoprotein-cholesterol; LMH, low-protein, moderate-carbohydrate, high-fat diet; LHL, low-protein, high-carbohydrate, low-fat diet; LHH, low-protein, high-carbohydrate, high-fat diet; HML, high-protein, moderate-carbohydrate, low-fat diet; HMH, high-protein, moderate-carbohydrate, high-fat diet; HHL, high-protein, high-carbohydrate, low-fat diet; HHH, high-protein, high-carbohydrate, high-fat diet.

**Supplementary data**

# Supplementary Table 13. League table for HDL-C

| LMH | -0.01  (-0.08, 0.06) | 0.03  (-0.03, 0.09) | 0.00  (-0.08, 0.07) | 0.01  (-0.04, 0.07) | -0.03  (-0.11, 0.04) | -0.02  (-0.11, 0.06) |
| --- | --- | --- | --- | --- | --- | --- |
|  | LHL | 0.04  (-0.01, 0.09) | 0.01  (-0.02, 0.04) | 0.03  (-0.02, 0.08) | -0.02  (-0.07, 0.03) | -0.01  (-0.06, 0.04) |
|  |  | LHH | -0.03  (-0.08, 0.02) | -0.01  (-0.04, 0.01) | **-0.06**  **(-0.12, -0.01)** | -0.05  (-0.11, 0.01) |
|  |  |  | HML | 0.02  (-0.04, 0.07) | -0.03  (-0.09, 0.02) | -0.02  (-0.08, 0.04) |
|  |  |  |  | HMH | -0.05  (-0.11, 0.01) | -0.04  (-0.10, 0.03) |
|  |  |  |  |  | HHL | 0.01  (-0.06, 0.08) |
|  |  |  |  |  |  | HHH |

^1^ The value above the treatments correspond to the difference in mean in HDL-C (mmol/L) between the column and the row.
HDL-C, high-density-lipoprotein-cholesterol; LMH, low-protein, moderate-carbohydrate, high-fat diet; LHL, low-protein, high-carbohydrate, low-fat diet; LHH, low-protein, high-carbohydrate, high-fat diet; HML, high-protein, moderate-carbohydrate, low-fat diet; HMH, high-protein, moderate-carbohydrate, high-fat diet; HHL, high-protein, high-carbohydrate, low-fat diet; HHH, high-protein, high-carbohydrate, high-fat diet.

**Supplementary data**

# **Supplementary Table 14.** Risk of bias assessment of selected randomized controlled trials for a systematic review, meta-regression, standard meta-analysis, and network meta-analysis studying the impact of protein quantity and type on cardiovascular disease risk factors in adults

| **Study ID** | **Randomization process** | **Bias arising from period and carryover effects**^1^ | **Deviations from intended interventions** | **Missing outcome data** | **Measurement of the outcome** | **Selection of the reported result** |
| --- | --- | --- | --- | --- | --- | --- |
| Ashton-2000 (51) | Low | Low | Some concerns | Low | Some concerns | Low |
| Azadbakht-2003 (52) | Low | Low | Some concerns | Low | Some concerns | Low |
| Azadbakht-2011 (54) | Low | Low | Some concerns | **High** | Some concerns | Low |
| Bahr-2013 (55) | Low | Low | Low | Low | Low | Low |
| Bahr-2015 (56) | Low | Low | Low | Low | Low | Low |
| Bakhit-1994 (57) | Low | **High** | Low | **High** | Some concerns | Low |
| Bergeron-2019 (60) | Low | Low | Low | Low | Low | Low |
| Borodin-2009 (61) | Low | Low | Some concerns | Low | Some concerns | Low |
| Bricarello-2004 (63) | Low | **High** | Low | Low | Low | Low |
| Crimarco-2020 (67) | Low | **High** | Low | Low | Low | Low |
| Cuevas-2003 (68) | Low | **High** | Low | Low | Low | Low |
| Desroches-2004 (69) | Low | Low | Low | Low | Low | Low |
| Fekete-2016 (12) | Low | Low | Low | Low | Low | Low |
| Frota-2015 (70) | Low | Low | Low | Low | Low | Low |
| Gardner-2007 (72) | Low | Low | Low | Low | Low | Low |
| He-2011 (17) | Low | Low | Low | Low | Low | Low |
| Jenkins-2000 (76) | Low | Low | Some concerns | Low | Some concerns | Low |
| Juraschek-2013 (24) | Low | Low | Low | Low | Low | Low |
| Kurowska-1997 (78) | Low | Low | Some concerns | Low | Some concerns | Low |
| Machin-2014 (25) | Low | Low | Some concerns | Low | Some concerns | Low |
| Matthan-2007 (85) | Low | Low | Some concerns | Low | Some concerns | Low |
| McVeigh-2006 (86) | Low | Low | Low | Low | Low | Low |
| Meinertz-1988 (87) | Low | Low | Some concerns | Low | Some concerns | Low |
| Meinertz-1989 (88) | Low | Low | Some concerns | Low | Some concerns | Low |
| Nilausen-1998 (89) | Low | Low | Some concerns | Low | Some concerns | Low |
| Papakonstantinou-2010 (32) | Low | Low | Low | Low | Low | Low |
| Pipe-2009 (91) | Low | Low | Low | Low | Low | Low |
| Roussell-2012 (35) | Low | Some concerns | Some concerns | Low | Some concerns | Low |
| Sacks-1984 (36) | Low | **High** | Low | Low | Some concerns | Low |
| Shige-1998 (96) | Low | Low | Some concerns | Low | Some concerns | Low |
| Stephenson-2005 (39) | Low | **High** | Some concerns | Some concerns | Some concerns | Low |
| Wang-1995 (107) | Low | **High** | Some concerns | Low | Some concerns | Low |
| Wiebe-1984 (109) | Low | **High** | Some concerns | Low | Some concerns | Low |
| Wolfe-1991 (46) | Low | **High** | Some concerns | Low | Some concerns | Low |
| Wolfe-1992 (47) | Low | **High** | Some concerns | Low | Some concerns | Low |
| Wolfe-1999 (48) | Low | Low | Some concerns | Low | Some concerns | Low |
| Abete-2009 (1) | Low | NA | Some concerns | Low | Low | Low |
| Aldrich-2011 (2) | Low | NA | Low | Low | Low | Low |
| Anderson-2007 (50) | Low | NA | Low | Some concerns | Low | Low |
| Azadbakht-2008 (53) | Low | NA | Low | Low | Low | Low |
| Azadbakht-2013 (3) | Low | NA | Low | Low | Low | Low |
| Basciani-2020 (58) | Low | NA | Low | Low | Some concerns | Low |
| Beavers-2010 (59) | Low | NA | Low | Low | Low | Low |
| Bergeron-2019 (60) | Low | NA | Low | Low | Low | Low |
| Bosello-1988 (62) | Low | NA | Some concerns | Low | Some concerns | Low |
| Bray-2020 (4) | Low | NA | Low | Low | Some concerns | Low |
| Brinkworth-2004 (5) | Low | NA | Low | Low | Low | Low |
| Campbell-2010 (64) | Low | NA | Low | Low | Low | Low |
| Chen-2005 (65) | Low | NA | Low | Low | Low | Low |
| Chen-2006 (66) | Low | NA | Low | Low | Low | Low |
| Chiu-2014 (6) | Low | NA | Low | Low | Low | Low |
| Claessens-2009 (7) | Low | NA | Some concerns | Low | Low | Low |
| Dalle Grave-2013 (8) | Low | NA | Low | Low | Low | Low |
| de Luis-2015 (9) | Low | NA | Low | Low | Some concerns | Low |
| Delbridge-2009 (10) | Low | NA | Some concerns | Low | Some concerns | Low |
| Farnsworth-2003 (11) | Low | NA | Low | Low | Some concerns | Low |
| Fernandes-2018 (13) | Low | NA | Low | Low | Low | Low |
| Ferrara-2006 (14) | Low | NA | Low | Low | Low | Low |
| Gardner-2001 (71) | Low | NA | Low | Low | Low | Low |
| George-2020 (73) | Low | NA | Low | Low | Low | Low |
| Gögebakan-2011 (15) | Low | NA | Some concerns | Low | Some concerns | Low |
| Gulati-2017 (16) | Low | NA | Some concerns | Low | Some concerns | Low |
| Hill-2015 (18) | Low | NA | Some concerns | Low | Low | Low |
| Hodgson-2006 (19) | Low | NA | Some concerns | Low | Some concerns | Low |
| Hodgson-2012 (20) | Low | NA | Low | Low | Low | Low |
| Hudson-2020 (21) | Low | NA | Low | Low | Low | Low |
| Jamilian-2015 (74) | Low | NA | Low | Low | Low | Low |
| Jassi-2010 (75) | Low | NA | Low | Low | Low | Low |
| Johnston-2004 (23) | Low | NA | Some concerns | Low | **High** | Low |
| Karamali-2018 (77) | Low | NA | Low | Low | Low | Low |
| Liao-2007 (79) | Low | NA | Some concerns | Low | Some concerns | Low |
| Liu-2012 (80) | Low | NA | Low | Low | Low | Low |
| Liu-2013 (81) | Low | NA | Low | Low | Low | Low |
| Liu-2014 (82) | Low | NA | Low | Low | Low | Low |
| Ma-2011 (83) | Low | NA | Low | Low | Low | Low |
| MacAuley-2006 (28) | Low | NA | Some concerns | Low | Some concerns | Low |
| Maki-2010 (84) | Low | NA | Low | Low | Low | Low |
| Mamo-2005 (26) | Low | NA | Low | Low | Low | Low |
| Mateo-Gallego-2017 (27) | Low | NA | Low | Low | Low | Low |
| Meckling-2007 (29) | Low | NA | Some concerns | **High** | Some concerns | Low |
| Mehrabani-2012 (30) | Low | NA | Some concerns | **High** | Some concerns | Low |
| Noakes-2005 (31) | Low | NA | Some concerns | Low | Some concerns | Low |
| Padhi-2015 (90) | Low | NA | Low | Low | Low | Low |
| Pedersen-2014 (33) | Low | NA | Low | Some concerns | Low | Low |
| Perscott-1987 (92) | Low | NA | Low | Low | Low | Low |
| Porter-2019 (34) | Low | NA | Some concerns | Some concerns | Some concerns | Low |
| Rivas-2002 (93) | Low | NA | Low | Low | Low | Low |
| Sacks-2009 (37) | Low | NA | Low | Low | Low | Low |
| Santo-2010 (94) | Low | NA | Low | Low | Low | Low |
| Sheikholeslami-2012 (38) | Low | NA | Some concerns | Low | **High** | Low |
| Shidfar-2009 (95) | Low | NA | Low | Low | Low | Low |
| Sirtori-2012 (97) | Low | NA | Low | Low | Low | Low |
| Sucher-2017 (98) | Low | NA | Some concerns | Low | Some concerns | Low |
| Tabibi-2010 (99) | Low | NA | Some concerns | Low | **High** | Low |
| Tahavorgar-2015 (100) | Low | NA | Low | Low | Low | Low |
| Tang-2013 (40) | Low | NA | Some concerns | Some concerns | Some concerns | Low |
| Teede-2001 (101) | Low | NA | Low | Low | Low | Low |
| Teixeira-2000 (102) | Low | NA | Some concerns | Low | Some concerns | Low |
| Teunisseti-Beekman-2012 (41) | Low | NA | Low | Low | Low | Low |
| Tischmann-2020 (42) | Low | NA | Some concerns | Low | Some concerns | Low |
| Tonstad-2002 (103) | Low | NA | Low | Some concerns | Low | Low |
| Toscani-2011 (43) | Low | NA | Some concerns | Low | Some concerns | Low |
| Treyzon-2008 (44) | Low | NA | Some concerns | Low | Some concerns | Low |
| van Raaij-1981 (104) | Low | NA | Some concerns | Low | Some concerns | Low |
| van Raaij-1982 (105) | Low | NA | Some concerns | Low | **High** | Low |
| Vigna-2000 (106) | Low | NA | Low | Some concerns | Low | Low |
| Weinheimer-2012 (45) | Low | NA | Low | Low | Low | Low |
| Weisse-2010 (108) | Low | NA | Low | Some concerns | Low | Low |
| Xu-2005 (110) | Low | NA | Low | Low | Low | Low |
| Yilmaz-2021 (49) | Low | NA | Low | Some concerns | Low | Low |

^1^Only for crossover trials.

NA, not applicable.

**Supplementary data**

# Supplementary Table 15. Side-splitting approach^1^ for inconsistency for HDL-C

| Side | Direct | | Indirect | | Difference | | |
| --- | --- | --- | --- | --- | --- | --- | --- |
|  | Estimate | SE | Estimate | SE | Estimate | SE | *P* value |
| A - E | 0.015 | 0.027 | -0.003 | 0.141 | 0.018 | 0.143 | 0.898 |
| **B - D** | -0.001 | 0.017 | 0.118 | 0.051 | -0.119 | 0.054 | **0.028** |
| B - E | 0.053 | 0.040 | 0.009 | 0.034 | 0.044 | 0.052 | 0.396 |
| B - F | -0.008 | 0.026 | -0.067 | 0.051 | 0.059 | 0.057 | 0.296 |
| B - G | -0.008 | 0.030 | -0.020 | 0.060 | 0.012 | 0.067 | 0.862 |
| **C - D** | 0.047 | 0.044 | -0.072 | 0.031 | 0.119 | 0.054 | **0.028** |
| C - E | -0.018 | 0.015 | 0.026 | 0.049 | -0.044 | 0.052 | 0.392 |
| C - F | -0.094 | 0.042 | -0.035 | 0.038 | -0.059 | 0.057 | 0.295 |
| C - G | -0.059 | 0.053 | -0.048 | 0.041 | -0.012 | 0.067 | 0.862 |

^1^*P* for the assessment of inconsistency. *P* < 0.05 was considered to indicate a significant inconsistency existed between direct and indirect evidence.

A, low-protein, moderate-carbohydrate, high-fat diet; B, low-protein, high-carbohydrate, low-fat diet; C, low-protein, high-carbohydrate, high-fat diet; D, high-protein, moderate-carbohydrate, low-fat diet; E, high-protein, moderate-carbohydrate, high-fat diet; F, high-protein, high-carbohydrate, low-fat diet; G, high-protein, high-carbohydrate, high-fat diet; HDL-C, high-density-lipoprotein-cholesterol; SE, standard error.

**Supplementary data**

# Supplementary Table 16. GRADE evaluation for selected primary outcomes and all comparisons between the different groups

|  | Direct evidence | | | Indirect evidence | | Network meta-analysis | |
| --- | --- | --- | --- | --- | --- | --- | --- |
| Comparison | Number of RCTs | MD (95% CI) | Certainty of evidence^1^ | MD (95% CI) | Certainty of evidence^2^ | MD (95% CI) | Certainty of evidence^3^ |
| **SBP (mmHg)** | | | | | | | |
| A - D | 0 | - | - | -1.89 (-8.02, 4.23) | High | -1.89 (-8.02, 4.23) | Moderate ^c^ |
| A - E | 2 | -0.78 (-5.39, 3.84) | Moderate ^a^ | -0.48 (-34.62, 33.65) | High | -0.77 (-5.35, 3.81) | Moderate ^c^ |
| A - F | 0 | - | - | -2.08 (-7.94, 3.78) | High | -2.08 (-7.94, 3.78) | Moderate ^c^ |
| A - G | 0 | - | - | -0.96 (-7.08, 5.17) | High | -0.96 (-7.08, 5.17) | Moderate ^c^ |
| B - D | 10 | -1.50 (-3.99, 0.98) | Low ^ab^ | 0.68 (-253.20, 254.56) | High | -1.89 (-8.02, 4.23) | Low ^ce^ |
| B - E | 2 | 0.30 (-4.10, 4.69) | Moderate ^a^ | -1.27 (-6.29, 3.74) | High | -0.77 (-5.35, 3.81) | Low ^ce^ |
| B - F | 6 | -1.35 (-4.59, 1.88) | Low ^ab^ | -3.02 (-9.48, 3.45) | High | -2.08 (-7.94, 3.78) | Moderate ^c^ |
| B - G | 3 | -1.53 (-5.23, 2.17) | Low ^ab^ | 3.10 (-4.13, 10.33) | High | -0.96 (-7.08, 5.17) | Low ^ce^ |
| C - D | 0 | - | - | -3.80 (-7.92, 0.32) | High | -3.80 (-7.92, 0.32) | Moderate ^c^ |
| C - E | 18 | -2.79 (-4.63, -0.95) | Low ^ab^ | -1.24 (-7.64, 5.16) | High | -0.38 (-3.64, 2.88) | Moderate ^c^ |
| C - F | 2 | -4.80 (-9.91, 0.30) | Moderate ^a^ | -3.14 (-8.27, 1.99) | High | -1.69 (-4.56, 1.18) | Moderate ^c^ |
| C - G | 1 | -0.14 (-6.38, 6.10) | Moderate ^a^ | -4.76 (-9.96, 0.44) | High | -0.57 (-3.90, 2.76) | Moderate ^c^ |
| **DBP (mmHg)** | | | | | | | |
| A - D | 0 | - | - | 0.78 (-3.63, 5.19) | High | 0.78 (-3.63, 5.19) | Moderate ^c^ |
| A - E | 2 | -0.36 (-3.68, 2.95) | Moderate ^a^ | -1.11 (-27.51, 25.29) | High | -0.37 (-3.67, 2.92) | Moderate ^c^ |
| A – F | 0 | - | - | -0.41 (-4.73, 3.92) | High | -0.41 (-4.73, 3.92) | Moderate ^c^ |
| A - G | 0 | - | - | 0.67 (-3.73, 5.08) | High | 0.67 (-3.73, 5.08) | Moderate ^c^ |
| B - D | 10 | -0.41 (-2.19, 1.36) | Low ^ab^ | 1.96 (-7.47, 11.38) | High | -0.33 (-2.07, 1.41) | Low ^ce^ |
| B - E | 2 | -2.99 (-6.45, 0.47) | Moderate ^a^ | 0.05 (-3.44, 3.54) | High | -1.49 (-3.96, 0.99) | Low ^ce^ |
| B - F | 7 | -0.77 (-2.84, 1.29) | Low ^ab^ | -6.05 (-11.09, 1.02) | High | -1.52 (-3.49, 0.45) | Moderate ^c^ |
| B - G | 3 | -0.54 (-3.20, 2.12) | Low ^ab^ | -0.01 (-5.28, 5.25) | High | -0.44 (-2.77, 1.90) | Moderate ^c^ |
| C - D | 1 | 1.52 (-7.56, 10.60) | Moderate ^a^ | -0.86 (-3.97, 2.26) | High | -0.61 (-3.54, 2.33) | Low ^ce^ |
| C - E | 17 | -1.56 (-2.85, -0.26) | Low ^ab^ | -4.56 (-9.29, -0.17) | High | -1.76 (-3.03, -0.50) | Moderate ^c^ |
| C - F | 1 | -5.00 (-9.22, -0.78) | Moderate ^a^ | 0.28 (-3.16, -3.72) | High | -1.80 (-4.57, 0.98) | Low ^ce^ |
| C - G | 1 | -0.40 (-4.82, 4.02) | Moderate ^a^ | -0.94 (-4.84, 2.96) | High | -0.71 (-3.59, 2.16) | Moderate ^c^ |
| **TC (mmol/L)** | | | | | | | |
| A - D | 0 | - | - | 0.01 (-0.23, 0.25) | High | 0.01 (-0.23, 0.25) | Moderate ^c^ |
| A - E | 5 | -0.04 (-0.20, 0.12) | Low ^ad^ | -0.07 (-2.86, 2.73) | High | -0.04 (-0.20, 0.12) | Moderate ^c^ |
| A - F | 0 | - | - | -0.10 (-0.35, 0.15) | High | -0.10 (-0.35, 0.15) | Moderate ^c^ |
| A - G | 0 | - | - | 0.01 (-0.25, 0.27) | High | 0.01 (-0.25, 0.27) | Moderate ^c^ |
| B - D | 14 | -0.06 (-0.17, 0.05) | Very low ^abd^ | 0.23 (-0.10, 0.56) | High | -0.03 (-0.14, 0.07) | Low ^ce^ |
| B - E | 2 | 0.00 (-0.21, 0.22) | Low ^ad^ | -0.16 (-0.38, 0.05) | High | -0.08 (-0.24, 0.07) | Moderate ^c^ |
| B - F | 7 | -0.13 (-0.30, 0.03) | Low ^ad^ | -0.17 (-0.50, 0.15) | High | -0.14 (-0.28, 0.00) | Moderate ^c^ |
| B - G | 4 | -0.02 (-0.21, 0.17) | Very low ^abd^ | -0.06 (-0.43, 0.31) | High | -0.03 (-0.19, 0.13) | Moderate ^c^ |
| C - D | 2 | 0.10 (-0.19, 0.39) | Low ^ad^ | -0.19 (-0.39, 0.01) | High | -0.10 (-0.26, 0.07) | Low ^ce^ |
| C - E | 16 | -0.17 (-0.27, -0.06) | Very low ^abd^ | 0.00 (-0.29, -0.29) | High | -0.15 (-0.24, -0.05) | High |
| C - F | 2 | -0.23 (-0.50, 0.04) | Low ^ad^ | -0.19 (-0.42, 0.05) | High | -0.21 (-0.38, -0.03) | High |
| C - G | 1 | -0.12 (-0.45, 0.21) | Low ^ad^ | -0.08 (-0.33, 0.17) | High | -0.09 (-0.29, 0.10) | Moderate ^c^ |
| **TG (mmol/L)** | | | | | | | |
| A - D | 0 | - | - | -0.08 (-0.39, 0.23) | High | -0.08 (-0.39, 0.23) | Moderate ^c^ |
| A - E | 5 | -0.03 (-0.25, 0.19) | Moderate ^a^ | -0.07 (-1.75, 1.60) | High | -0.03 (-0.25, 0.19) | Moderate ^c^ |
| A – F | 0 | - | - | 0.11 (-0.21, 0.43) | High | 0.11 (-0.21, 0.43) | Moderate ^c^ |
| A - G | 0 | - | - | 0.02 (-0.33, 0.36) | High | 0.02 (-0.33, 0.36) | Moderate ^c^ |
| B - D | 14 | -0.09 (-0.23, 0.04) | Low ^ab^ | 0.11 (-0.29, 0.51) | High | -0.07 (-0.20, 0.06) | Low ^ce^ |
| B - E | 2 | 0.03 (-0.29, 0.34) | Moderate ^a^ | -0.05 (-0.32, 0.22) | High | -0.02 (-0.22, 0.18) | Low ^ce^ |
| B - F | 7 | 0.16 (-0.04, 0.36) | Moderate ^a^ | -0.02 (-0.41, 0.38) | High | 0.12 (-0.05, 0.30) | Low ^ce^ |
| B - G | 4 | 0.02 (-0.21, 0.25) | Low ^ab^ | 0.09 (-0.41, 0.58) | High | 0.03 (-0.17, 0.23) | Moderate ^c^ |
| C - D | 2 | -0.10 (-0.43, 0.23) | Low ^ab^ | -0.31 (-0.57, 0.04) | High | -0.22 (-0.43, -0.02) | High |
| C - E | 13 | -0.18 (-0.32, -0.04) | Low ^ab^ | -0.10 (-0.49, -0.29) | High | -0.17 (-0.30, -0.04) | High |
| C - F | 2 | -0.13 (-0.45, 0.20) | Low ^ab^ | 0.05 (-0.25, 0.35) | High | -0.03 (-0.25, 0.19) | Low ^ce^ |
| C - G | 1 | -0.08 (-0.53, 0.38) | Moderate ^a^ | -0.14 (-0.45, 0.17) | High | -0.12 (-0.37, 0.13) | Moderate ^c^ |
| **LDL-C (mmol/L)** | | | | | | | |
| A - D | 0 | - | - | -0.01 (-0.22, 0.21) | High | -0.01 (-0.22, 0.21) | Moderate ^c^ |
| A - E | 5 | -0.01 (-0.15, 0.13) | Moderate ^a^ | -0.04 (-2.02, 1.95) | High | -0.01 (-0.15, 0.13) | Moderate ^c^ |
| A – F | 0 | - | - | -0.10 (-0.33, 0.12) | High | -0.10 (-0.33, 0.12) | Moderate ^c^ |
| A - G | 0 | - | - | 0.07 (-0.16, 0.30) | High | 0.07 (-0.16, 0.30) | Moderate ^c^ |
| B - D | 14 | -0.04 (-0.15, 0.06) | Low ^ab^ | 0.17 (-0.18, 0.53) | High | -0.03 (-0.13, 0.08) | Moderate ^c^ |
| B - E | 2 | 0.02 (-0.16, 0.21) | Moderate ^a^ | -0.09 (-0.30, 0.12) | High | -0.03 (-0.17, 0.11) | Low ^ce^ |
| B - F | 5 | -0.11 (-0.27, 0.06) | Moderate ^a^ | -0.18 (-0.47, 0.11) | High | -0.12 (-0.27, 0.02) | Moderate ^c^ |
| B - G | 4 | 0.03 (-0.14, 0.20) | Low ^ab^ | 0.11 (-0.21, 0.42) | High | 0.05 (-0.10, 0.19) | Moderate ^c^ |
| C - D | 2 | 0.08 (-0.24, 0.40) | Low ^ab^ | -0.14 (-0.32, 0.04) | High | -0.09 (-0.25, 0.07) | Low ^ce^ |
| C - E | 15 | -0.10 (-0.20, 0.00) | Low ^ab^ | 0.01 (-0.25, 0.27) | High | -0.09 (-0.18, 0.00) | Low ^ce^ |
| C - F | 2 | -0.23 (-0.47, 0.01) | Low ^ab^ | -0.15 (-0.38, 0.08) | High | -0.19 (-0.35, -0.02) | High |
| C - G | 1 | 0.03 (-0.25, 0.31) | Moderate ^a^ | -0.04 (-0.27, 0.19) | High | -0.01 (-0.19, 0.16) | Low ^ce^ |
| **HDL-C (mmol/L)** | | | | | | | |
| A - D | 0 | - | - | 0.00 (-0.08, 0.07) | High | 0.00 (-0.08, 0.07) | Moderate ^c^ |
| A - E | 5 | 0.01 (-0.04, 0.07) | Moderate ^a^ | 0.00 (-0.28, 0.27) | High | 0.01 (-0.04, 0.07) | Moderate ^c^ |
| A – F | 0 | - | - | -0.03 (-0.11, 0.04) | High | -0.03 (-0.11, 0.04) | Moderate ^c^ |
| A - G | 0 | - | - | -0.02 (-0.11, 0.06) | High | -0.02 (-0.11, 0.06) | Moderate ^c^ |
| B - D | 14 | 0.00 (-0.03, 0.03) | Low ^ab^ | 0.12 (0.02, 0.22) | High | 0.01 (-0.02, 0.04) | Moderate ^c^ |
| B - E | 2 | 0.05 (-0.02, 0.13) | Moderate ^a^ | 0.01 (-0.06, 0.08) | High | 0.03 (-0.02, 0.08) | Moderate ^c^ |
| B - F | 7 | -0.01 (-0.06, 0.04) | Moderate ^a^ | -0.07 (-0.17, 0.03) | High | -0.02 (-0.07, 0.03) | Moderate ^c^ |
| B - G | 4 | -0.01 (-0.07, 0.05) | Low ^ab^ | -0.02 (-0.14, 0.10) | High | -0.01 (-0.06, 0.04) | Moderate ^c^ |
| C - D | 2 | 0.05 (-0.04, 0.13) | Low ^ab^ | -0.07 (-0.13, 0.01) | High | -0.03 (-0.08, 0.02) | Low ^ce^ |
| C - E | 16 | -0.02 (-0.05, 0.01) | Low ^ab^ | 0.03 (-0.07, 0.12) | High | -0.01 (-0.04, 0.01) | Low ^ce^ |
| C - F | 2 | -0.09 (-0.18, -0.01) | Low ^ab^ | -0.03 (-0.11, -0.04) | High | -0.06 (-0.12, -0.01) | High |
| C - G | 1 | -0.06 (-0.16, 0.05) | Moderate ^a^ | -0.05 (-0.13, 0.03) | High | -0.05 (-0.11, 0.01) | Moderate ^c^ |

^1^ The rating of the certainty of the direct estimates considers risk of bias, indirectness, inconsistency, and publication bias. As suggested recently by the GRADE working group, consideration of imprecision is not necessary when rating the direct and indirect estimates to inform the rating of NMA estimates.

^2^ Only intransitivity was assessed from the indirect estimates. Not downgraded for intransitivity because there was no evidence for an uneven distribution of effect modifiers.

^3^ Incoherence was assessed from the network estimates.

^a^ Downgraded because of inconsistency (unexplained substantial heterogeneity, I^2^ ≥ 50%, *P* < 0.10).

^b^ Downgraded because of risk of bias (≥ 1 RCT with high risk of bias).

^c^ Downgraded because of imprecision (confidence intervals were wide and included or were close to null effect).

^d^ Downgraded because of publication bias (asymmetry visually identified on comparison-adjusted funnel plot).

^e^ Downgraded because of incoherence (dominant estimate not similar to network estimate).

A, low-protein, moderate-carbohydrate, high-fat diet; B, low-protein, high-carbohydrate, low-fat diet; C, low-protein, high-carbohydrate, high-fat diet; D, high-protein, moderate-carbohydrate, low-fat diet; E, high-protein, moderate-carbohydrate, high-fat diet; F, high-protein, high-carbohydrate, low-fat diet; G, high-protein, high-carbohydrate, high-fat diet; DBP, diastolic blood pressure; GRADE, Grading of Recommendations Assessment, Development, and Evaluation; HDL-C, high-density-lipoprotein-cholesterol; LDL-C, low-density-lipoprotein-cholesterol; MD, mean difference; NMA, network meta-analysis; SBP, systolic blood pressure; TC, total cholesterol; TG, triglyceride.

**Supplementary data**

# **Supplementary Table 17.** Side-splitting approach^1^ for inconsistency for SBP

| Side | Direct | | Indirect | | Difference | | |
| --- | --- | --- | --- | --- | --- | --- | --- |
|  | Estimate | SE | Estimate | SE | Estimate | SE | *P* value |
| A - E | -0.775 | 2.357 | -0.484 | 17.418 | -0.291 | 17.577 | 0.987 |
| B - D | -1.504 | 1.269 | 0.680 | 129.532 | -2.185 | 129.539 | 0.987 |
| B - E | 0.298 | 2.244 | -1.273 | 2.559 | 1.571 | 3.404 | 0.644 |
| B - F | -1.354 | 1.649 | -3.015 | 3.300 | 1.662 | 3.690 | 0.652 |
| B - G | -1.531 | 1.890 | 3.100 | 3.688 | -4.631 | 4.145 | 0.264 |
| C - E | -2.792 | 0.938 | -1.239 | 3.267 | -1.552 | 3.398 | 0.648 |
| C - F | -4.802 | 2.604 | -3.140 | 2.616 | -1.662 | 3.690 | 0.652 |
| C - G | -0.140 | 3.183 | -4.759 | 2.652 | 4.619 | 4.143 | 0.265 |

^1^*P* for the assessment of inconsistency. *P* < 0.05 was considered to indicate a significant inconsistency existed between direct and indirect evidence.

A, low-protein, moderate-carbohydrate, high-fat diet; B, low-protein, high-carbohydrate, low-fat diet; C, low-protein, high-carbohydrate, high-fat diet; D, high-protein, moderate-carbohydrate, low-fat diet; E, high-protein, moderate-carbohydrate, high-fat diet; F, high-protein, high-carbohydrate, low-fat diet; G, high-protein, high-carbohydrate, high-fat diet; SBP, systolic blood pressure; SE, standard error.

**Supplementary data**

# **Supplementary Table 18.** Side-splitting approach1 for inconsistency for DBP

| Side | Direct | | Indirect | | Difference | | |
| --- | --- | --- | --- | --- | --- | --- | --- |
|  | Estimate | SE | Estimate | SE | Estimate | SE | *P* value |
| A - E | -0.363 | 1.693 | -1.112 | 13.469 | 0.749 | 13.575 | 0.956 |
| B - D | -0.413 | 0.905 | 1.957 | 4.808 | -2.370 | 4.892 | 0.628 |
| B - E | -2.994 | 1.765 | 0.051 | 1.780 | -3.045 | 2.507 | 0.224 |
| B - F | -0.772 | 1.054 | -6.054 | 2.568 | 5.282 | 2.776 | 0.057 |
| B - G | -0.542 | 1.357 | -0.014 | 2.687 | -0.529 | 3.012 | 0.861 |
| C - D | 1.520 | 4.631 | -0.858 | 1.590 | 2.378 | 4.896 | 0.627 |
| C - E | -1.556 | 0.662 | -4.562 | 2.413 | 3.006 | 2.501 | 0.229 |
| C - F | -5.000 | 2.151 | 0.282 | 1.755 | -5.282 | 2.776 | 0.057 |
| C - G | -0.400 | 2.256 | -0.941 | 1.990 | 0.541 | 3.009 | 0.857 |

^1^*P* for the assessment of inconsistency. *P* < 0.05 was considered to indicate a significant inconsistency existed between direct and indirect evidence.

A, low-protein, moderate-carbohydrate, high-fat diet; B, low-protein, high-carbohydrate, low-fat diet; C, low-protein, high-carbohydrate, high-fat diet; D, high-protein, moderate-carbohydrate, low-fat diet; DBP, diastolic blood pressure; E, high-protein, moderate-carbohydrate, high-fat diet; F, high-protein, high-carbohydrate, low-fat diet; G, high-protein, high-carbohydrate, high-fat diet; SE, standard error.

**Supplementary data**

# Supplementary Table 19. Side-splitting approach^1^ for inconsistency for TC

| Side | Direct | | Indirect | | Difference | | |
| --- | --- | --- | --- | --- | --- | --- | --- |
|  | Estimate | SE | Estimate | SE | Estimate | SE | *P* value |
| A - E | -0.039 | 0.083 | -0.066 | 1.427 | 0.026 | 1.429 | 0.985 |
| B - D | -0.062 | 0.056 | 0.231 | 0.170 | -0.293 | 0.179 | 0.101 |
| B - E | 0.004 | 0.112 | -0.165 | 0.110 | 0.169 | 0.157 | 0.281 |
| B - F | -0.134 | 0.082 | -0.174 | 0.165 | 0.040 | 0.184 | 0.827 |
| B - G | -0.020 | 0.095 | -0.062 | 0.188 | 0.042 | 0.211 | 0.844 |
| C - D | 0.100 | 0.147 | -0.194 | 0.102 | 0.293 | 0.179 | 0.101 |
| C - E | -0.165 | 0.053 | 0.003 | 0.147 | -0.169 | 0.156 | 0.281 |
| C - F | -0.229 | 0.139 | -0.189 | 0.120 | -0.040 | 0.184 | 0.827 |
| C - G | -0.119 | 0.168 | -0.077 | 0.127 | -0.042 | 0.211 | 0.844 |

^1^*P* for the assessment of inconsistency. *P* < 0.05 was considered to indicate a significant inconsistency existed between direct and indirect evidence.

A, low-protein, moderate-carbohydrate, high-fat diet; B, low-protein, high-carbohydrate, low-fat diet; C, low-protein, high-carbohydrate, high-fat diet; D, high-protein, moderate-carbohydrate, low-fat diet; E, high-protein, moderate-carbohydrate, high-fat diet; F, high-protein, high-carbohydrate, low-fat diet; G, high-protein, high-carbohydrate, high-fat diet; SE, standard error; TC, total cholesterol.

**Supplementary data**

# Supplementary Table 20. Side-splitting approach^1^ for inconsistency for TG

| Side | Direct | | Indirect | | Difference | | |
| --- | --- | --- | --- | --- | --- | --- | --- |
|  | Estimate | SE | Estimate | SE | Estimate | SE | *P* value |
| A - E | -0.029 | 0.113 | -0.075 | 0.853 | 0.046 | 0.860 | 0.958 |
| B - D | -0.094 | 0.070 | 0.112 | 0.203 | -0.206 | 0.215 | 0.337 |
| B - E | 0.028 | 0.160 | -0.053 | 0.138 | 0.081 | 0.212 | 0.702 |
| B - F | 0.160 | 0.102 | -0.017 | 0.201 | 0.177 | 0.225 | 0.432 |
| B - G | 0.019 | 0.116 | 0.085 | 0.254 | -0.066 | 0.279 | 0.812 |
| C - D | -0.100 | 0.167 | -0.306 | 0.135 | 0.206 | 0.215 | 0.337 |
| C - E | -0.180 | 0.071 | -0.098 | 0.198 | -0.081 | 0.210 | 0.699 |
| C - F | -0.126 | 0.166 | 0.051 | 0.152 | -0.177 | 0.225 | 0.432 |
| C - G | -0.077 | 0.231 | -0.143 | 0.158 | 0.066 | 0.279 | 0.812 |

^1^*P* for the assessment of inconsistency. *P* < 0.05 was considered to indicate a significant inconsistency existed between direct and indirect evidence.

A, low-protein, moderate-carbohydrate, high-fat diet; B, low-protein, high-carbohydrate, low-fat diet; C, low-protein, high-carbohydrate, high-fat diet; D, high-protein, moderate-carbohydrate, low-fat diet; E, high-protein, moderate-carbohydrate, high-fat diet; F, high-protein, high-carbohydrate, low-fat diet; G, high-protein, high-carbohydrate, high-fat diet; SE, standard error; TG, triglyceride.

**Supplementary data**

# Supplementary Table 21. Side-splitting approach^1^ for inconsistency for LDL-C

| Side | Direct | | Indirect | | Difference | | |
| --- | --- | --- | --- | --- | --- | --- | --- |
|  | Estimate | SE | Estimate | SE | Estimate | SE | *P* value |
| A - E | -0.007 | 0.072 | -0.035 | 1.012 | 0.028 | 1.015 | 0.978 |
| B - D | -0.043 | 0.054 | 0.174 | 0.180 | -0.216 | 0.188 | 0.251 |
| B - E | 0.025 | 0.096 | -0.091 | 0.106 | 0.115 | 0.143 | 0.418 |
| B - F | -0.106 | 0.085 | -0.181 | 0.147 | 0.074 | 0.169 | 0.661 |
| B - G | 0.032 | 0.085 | 0.106 | 0.162 | -0.074 | 0.183 | 0.688 |
| C - D | 0.076 | 0.163 | -0.141 | 0.094 | 0.216 | 0.188 | 0.251 |
| C - E | -0.104 | 0.051 | 0.012 | 0.133 | -0.115 | 0.142 | 0.418 |
| C - F | -0.225 | 0.122 | -0.151 | 0.117 | -0.074 | 0.169 | 0.661 |
| C - G | 0.031 | 0.141 | -0.043 | 0.117 | 0.074 | 0.183 | 0.688 |

^1^*P* for the assessment of inconsistency. *P* < 0.05 was considered to indicate a significant inconsistency existed between direct and indirect evidence.

A, low-protein, moderate-carbohydrate, high-fat diet; B, low-protein, high-carbohydrate, low-fat diet; C, low-protein, high-carbohydrate, high-fat diet; D, high-protein, moderate-carbohydrate, low-fat diet; E, high-protein, moderate-carbohydrate, high-fat diet; F, high-protein, high-carbohydrate, low-fat diet; G, high-protein, high-carbohydrate, high-fat diet; LDL-C, low-density-lipoprotein-cholesterol; SE, standard error.

**Supplementary data**

Supplementary Figure 1. Network diagrams for SBP (A), DBP (B), TC (C), TG (D), LDL-C (E), and HDL-C (F). The size of the nodes is proportional to the total number of participants allocated to the intervention and the thickness of the lines proportional to the number of studies evaluating each direct comparison. HHH, high-protein, high-carbohydrate, high-fat diet; HHL, high-protein, high-carbohydrate, low-fat diet; HMH, high-protein, moderate-carbohydrate, high-fat diet; HML, high-protein, moderate-carbohydrate, low-fat diet; LHH, low-protein, high-carbohydrate, high-fat diet; LHL, low-protein, high-carbohydrate, low-fat diet; LMH, low-protein, moderate-carbohydrate, high-fat diet.


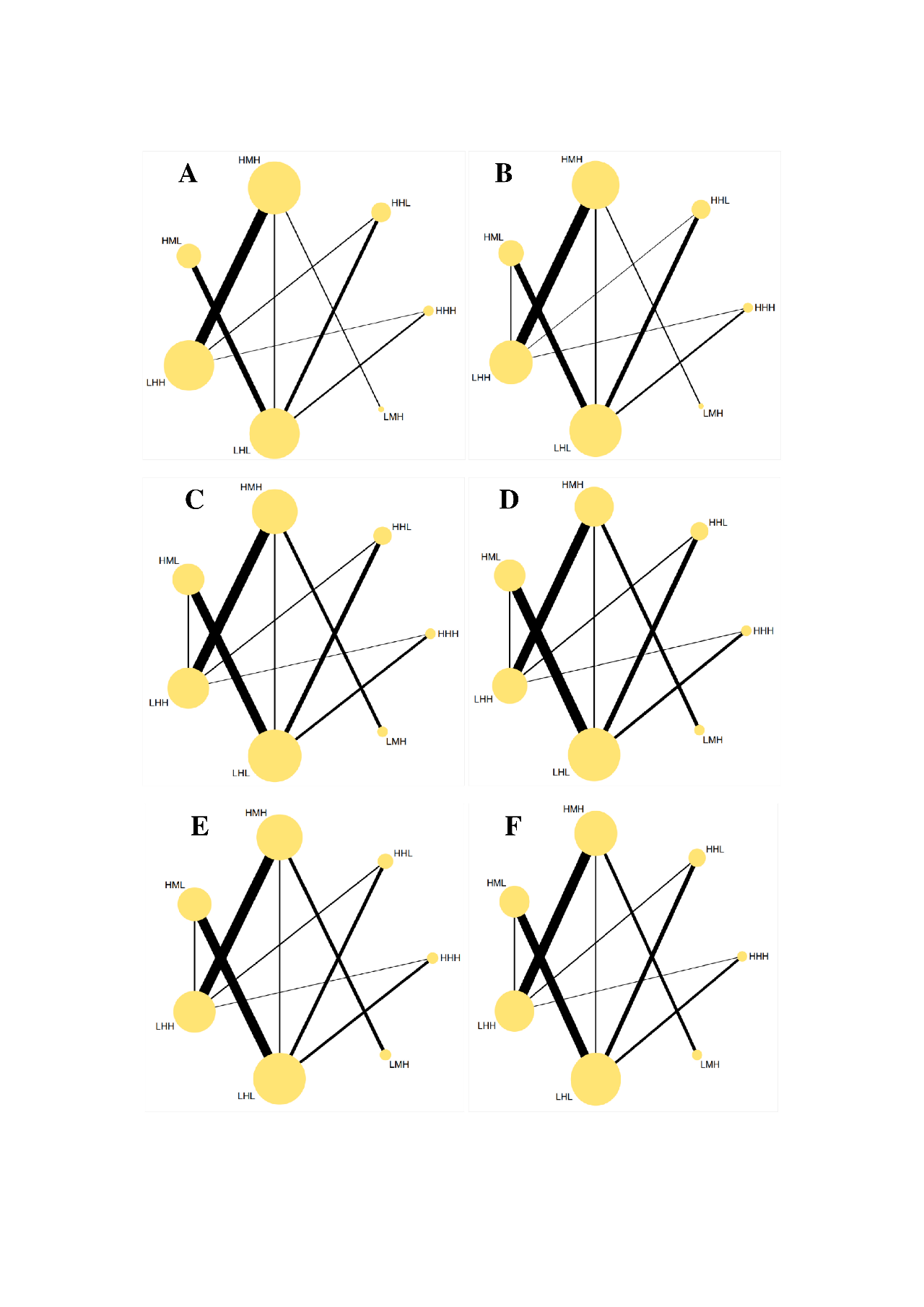


**Supplementary data**

# Supplementary Figure 2. Bubble plots for SBP (A), DBP (B), TC (C), TG (D), LDL-C (E), and HDL-C (F) in meta-regression analysis.


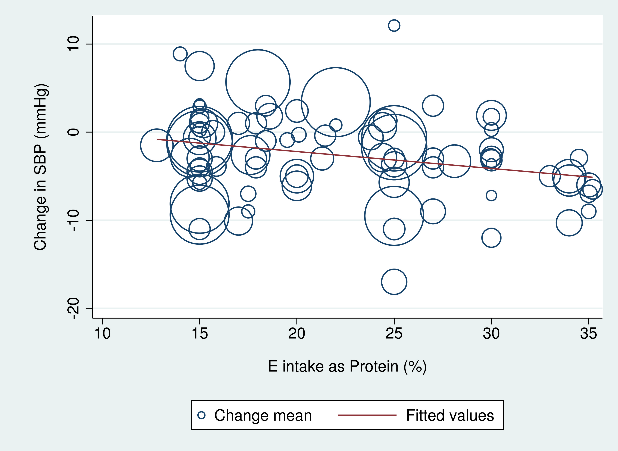

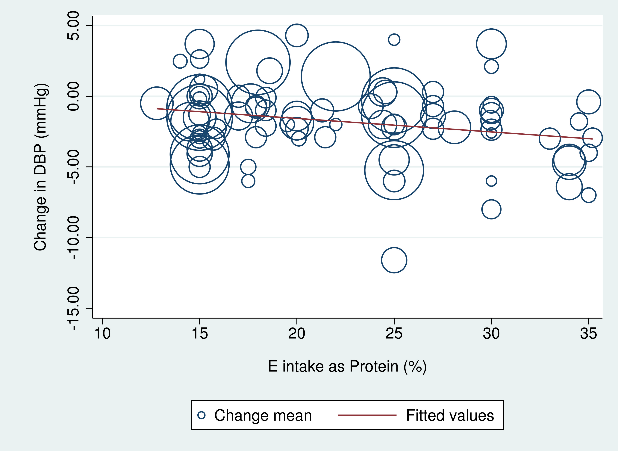


**B**

**A**


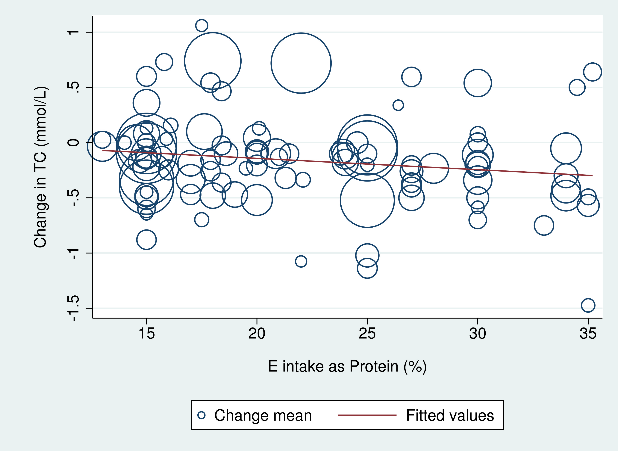

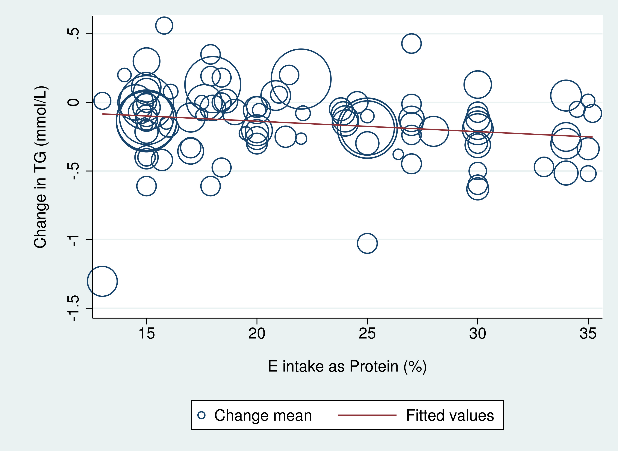


**D**

**C**


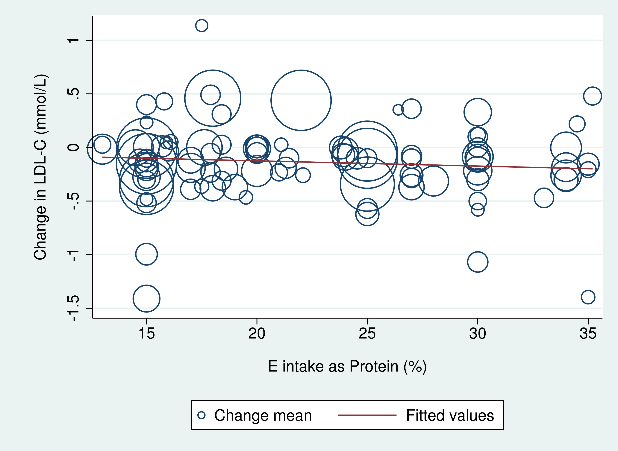

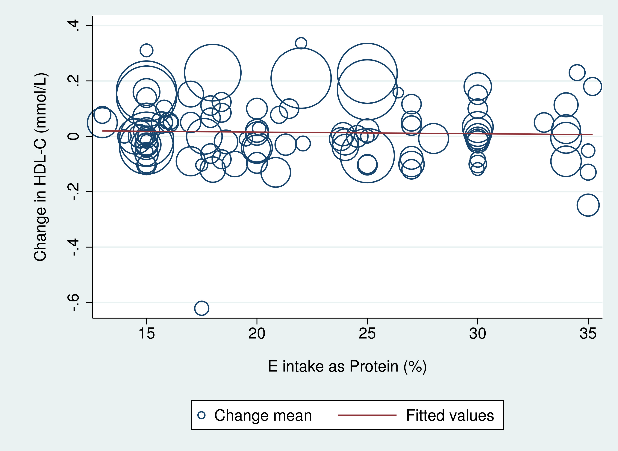


**F**

**E**

**Supplementary data**

# Supplementary Figure 3. Forest plots for TC (A), TG (B), LDL-C (C), HDL-C (D), CRP (E), IL-6 (F), and TNF-α (G) in standard meta-analysis for quantity-related studies.


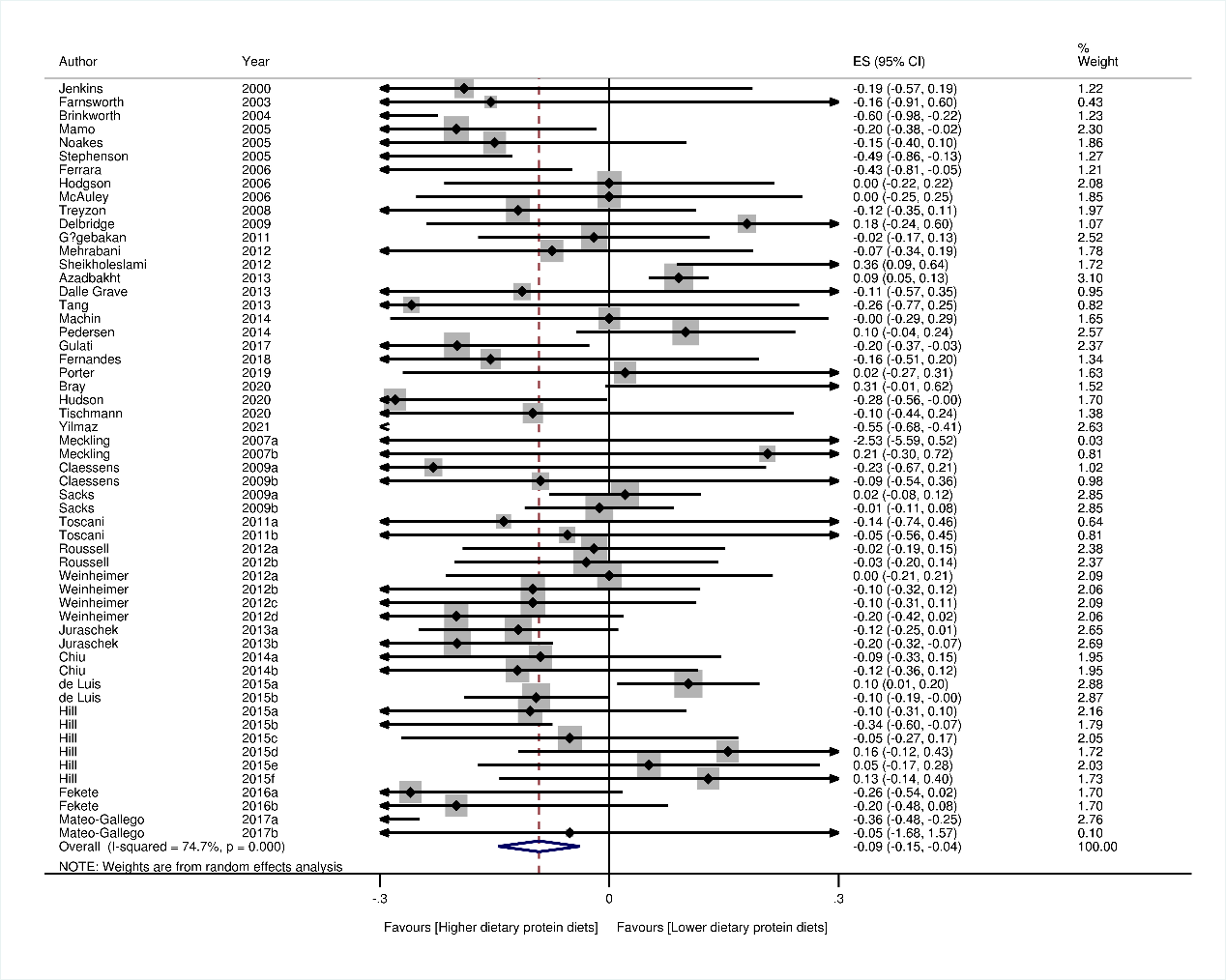


**A**


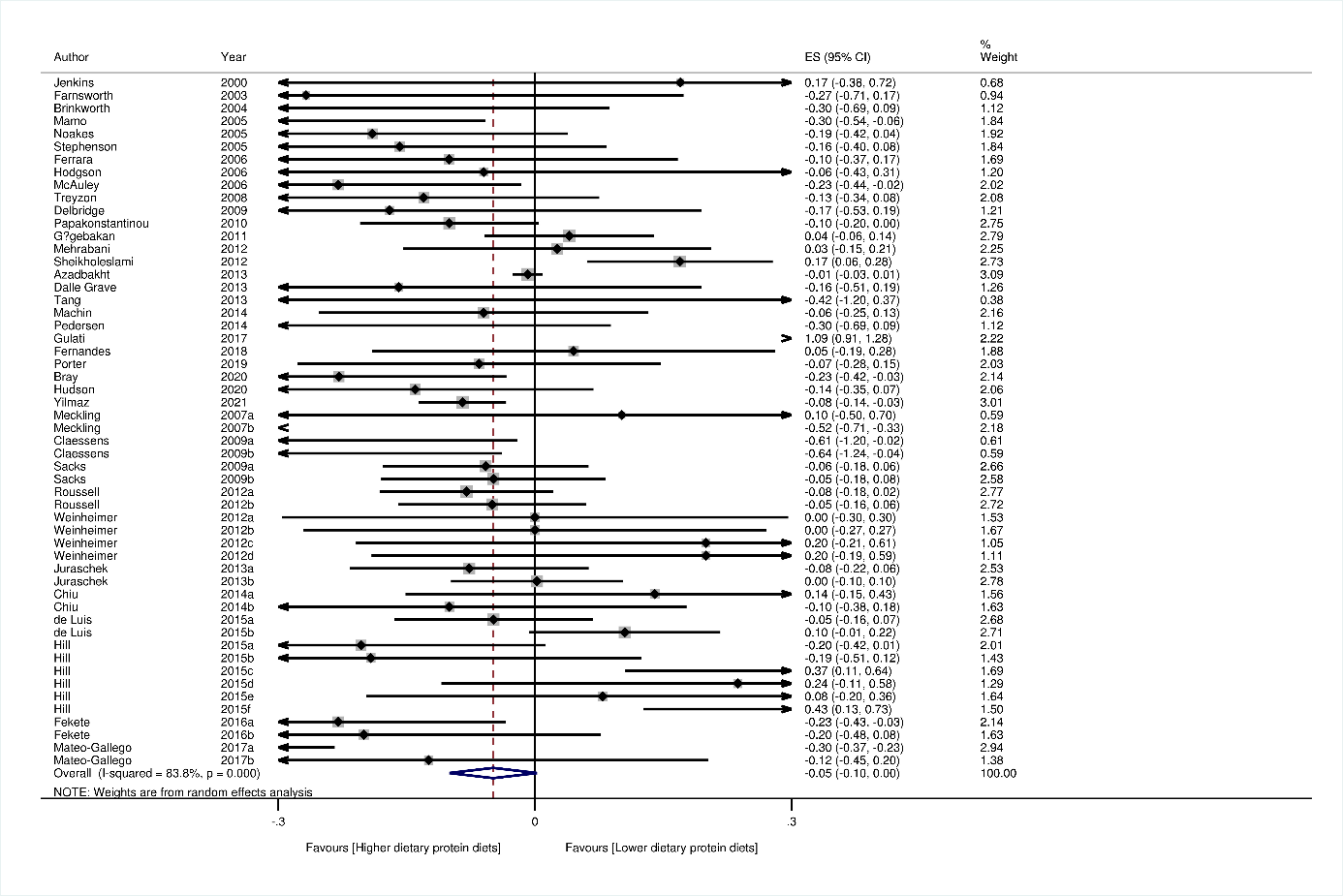


**B**


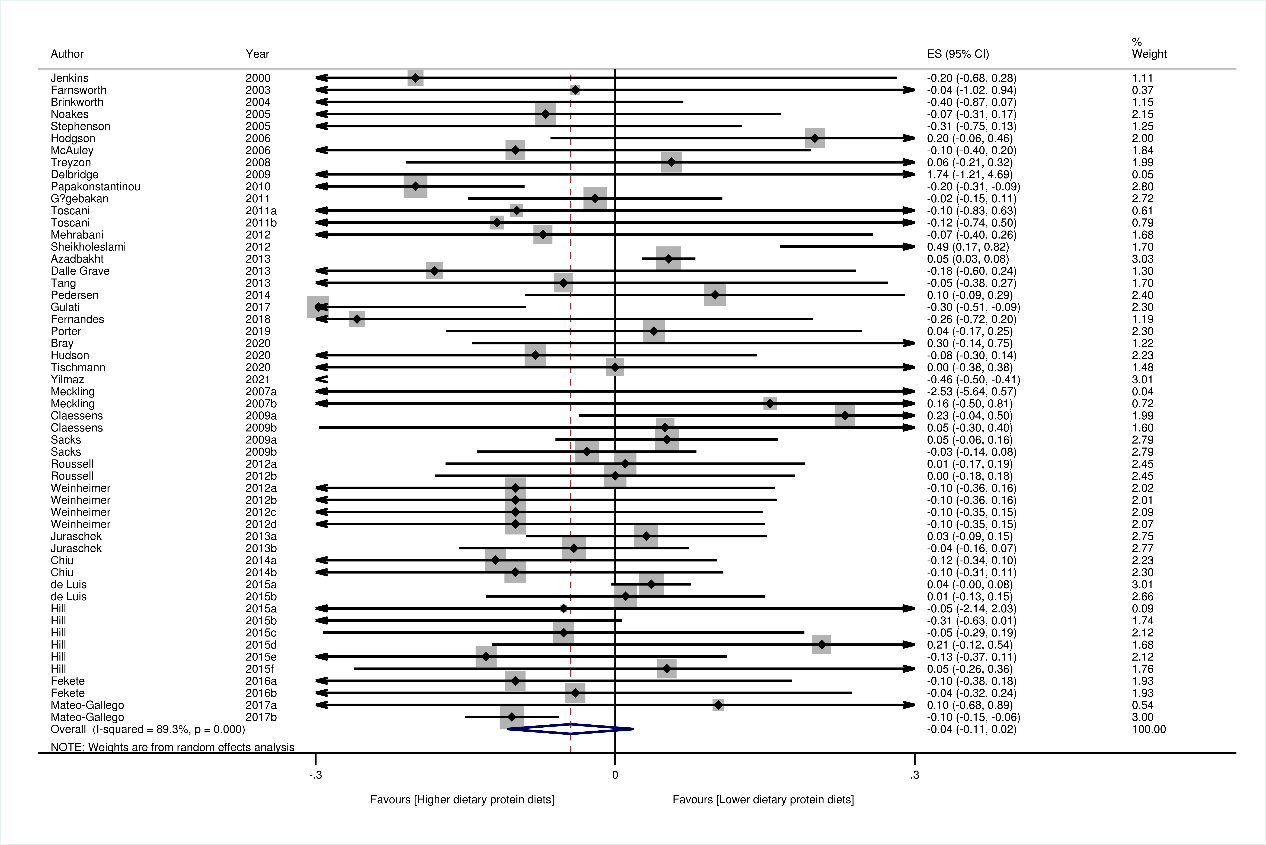


**C**


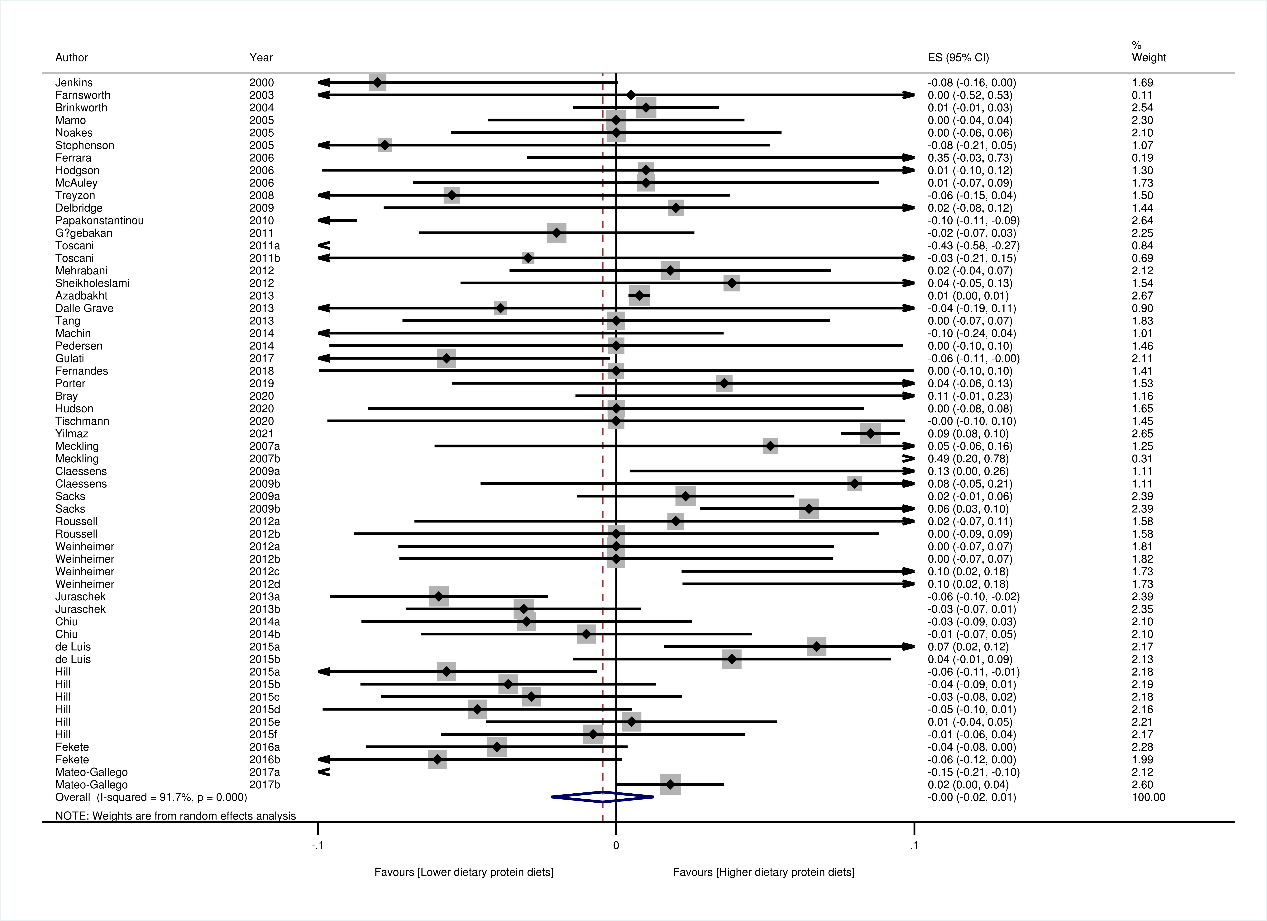


**D**


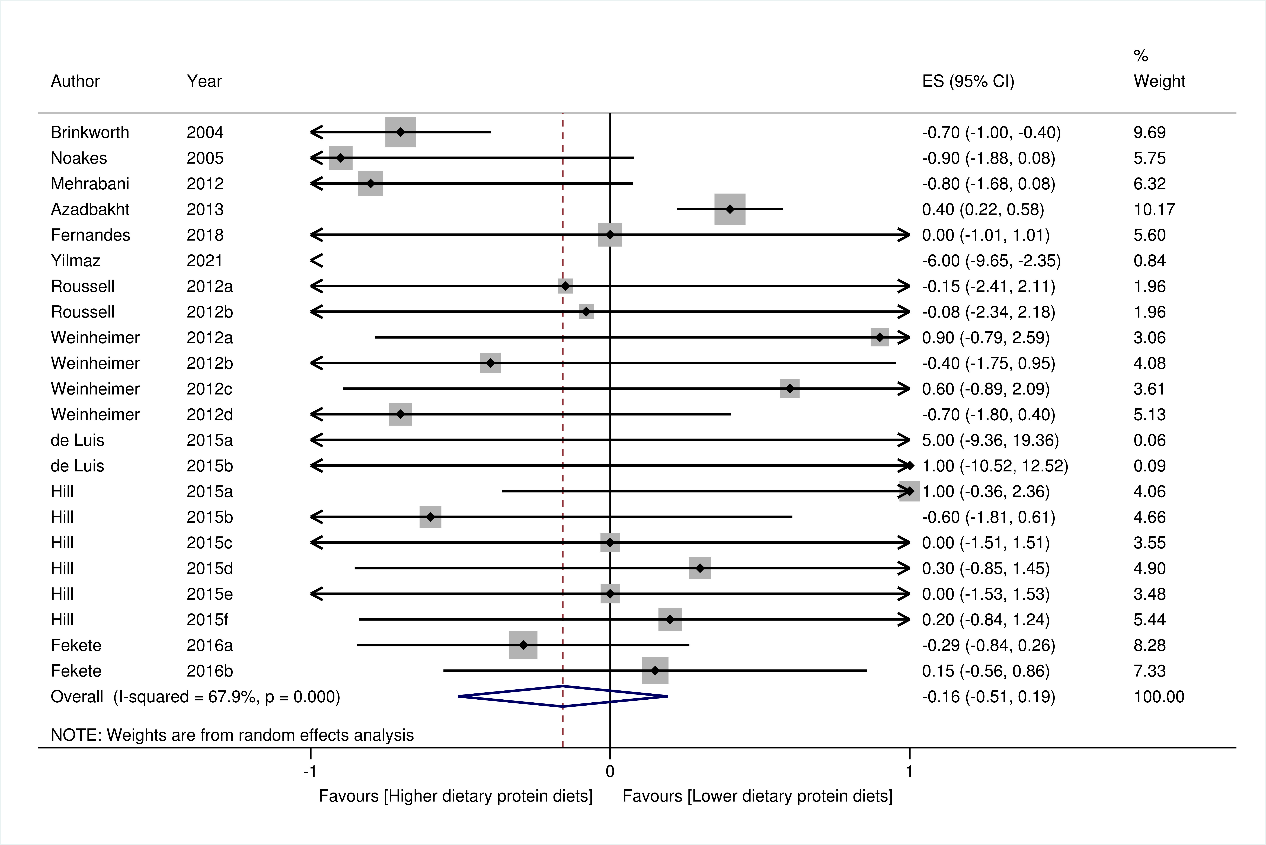


**E**


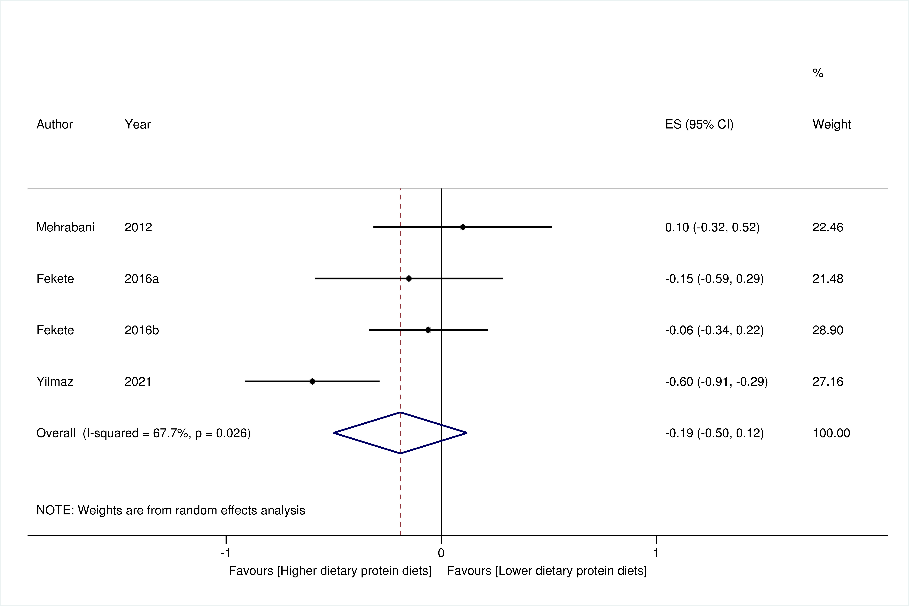


**F**


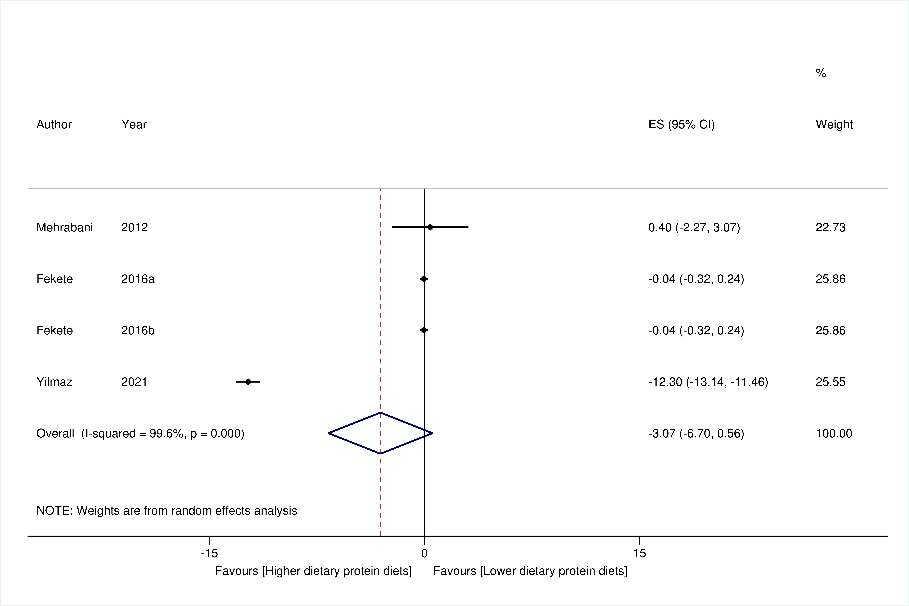


**G**

**Supplementary data**

# Supplementary Figure 4. Forest plots for SBP (A), DBP (B), FMD (C), and CRP (D) in standard meta-analysis for type-related studies.


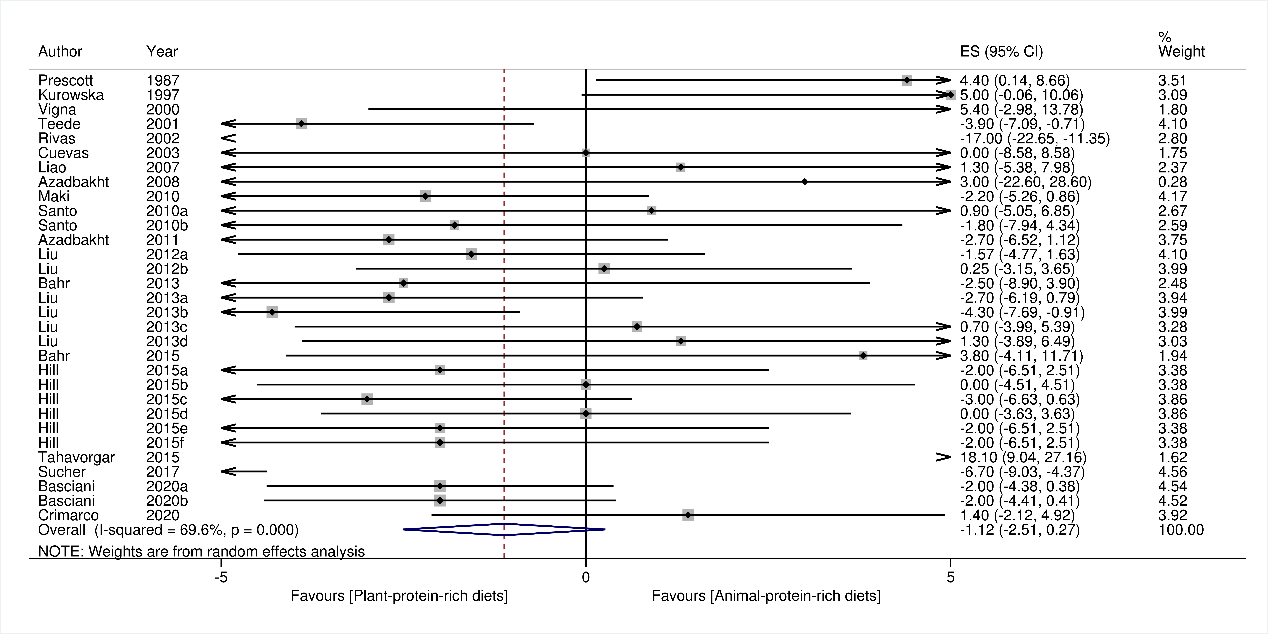


**A**


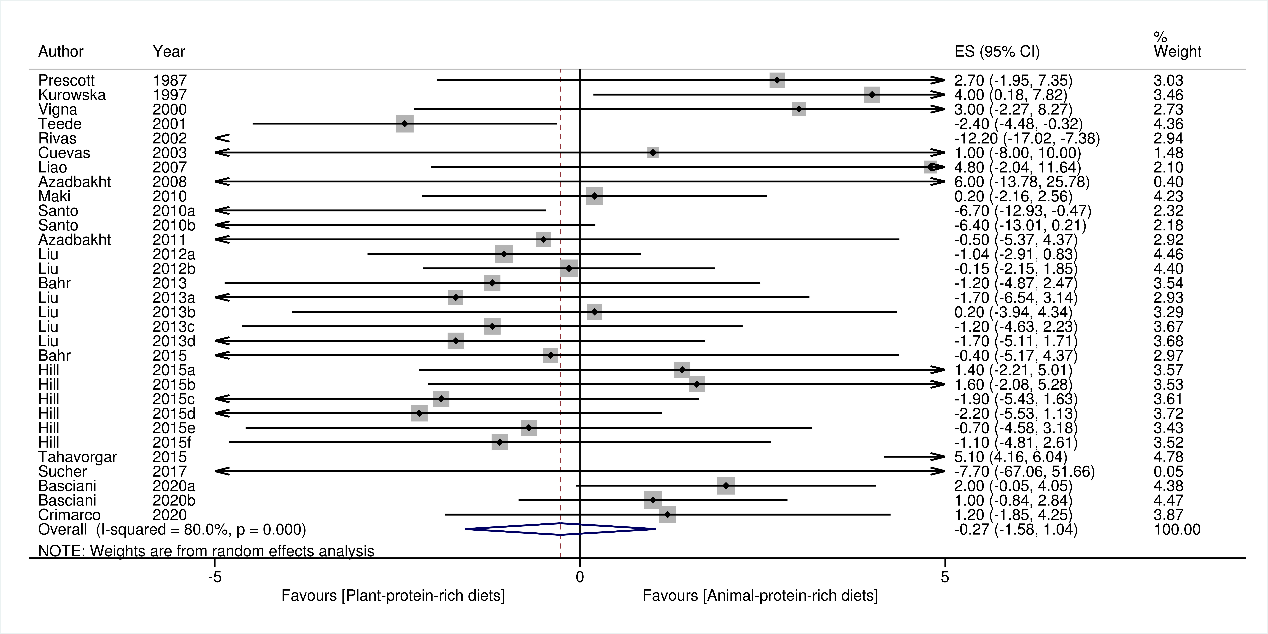


**B**


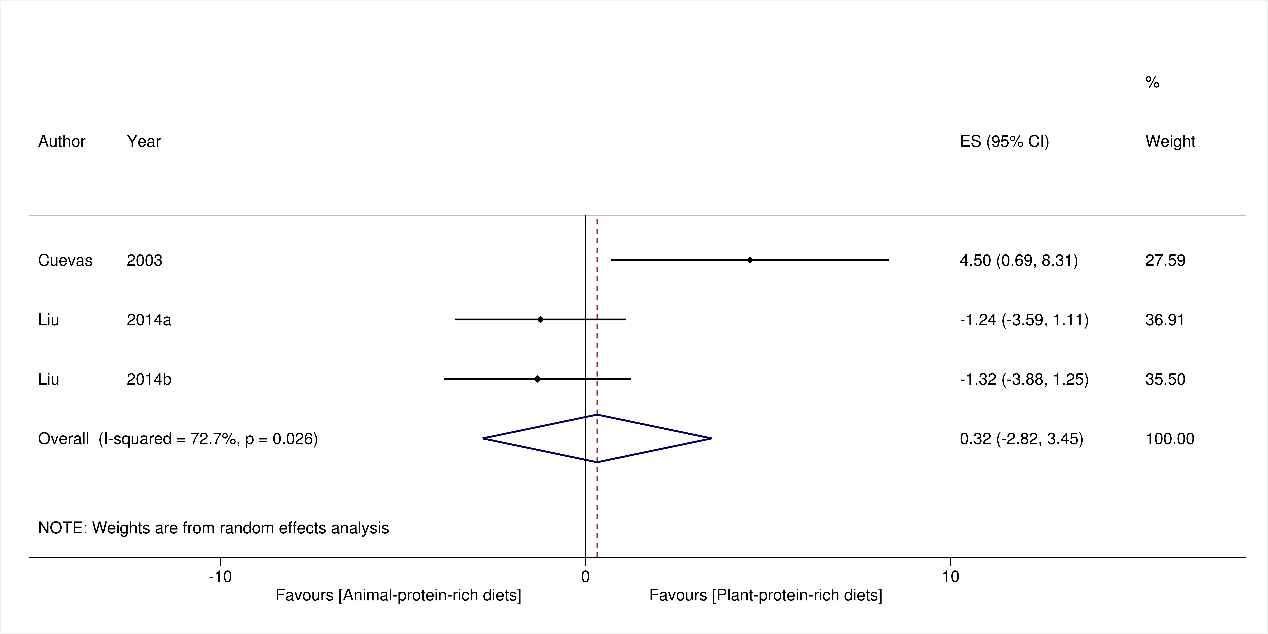


**C**


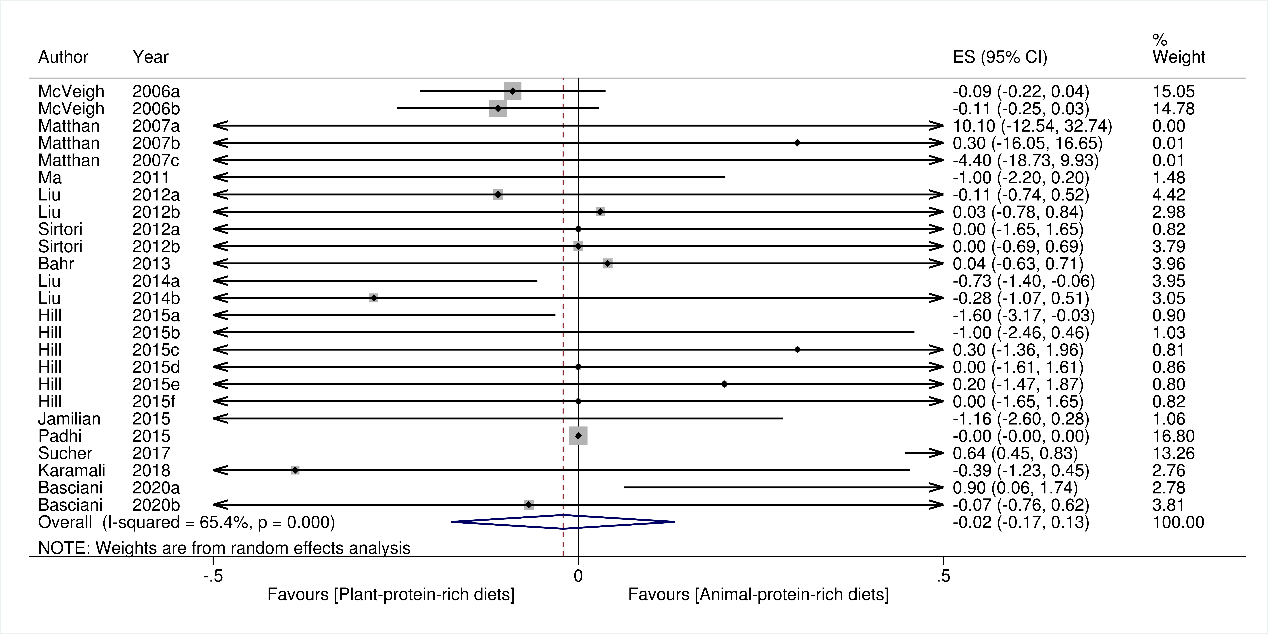


**D**

**Supplementary data**

# Supplementary Figure 5. Box plots showing the distributions of BMI (A), age (B), % male participants (C), study duration (D), sample size (E) across the available direct comparisons in the network meta-analysis.


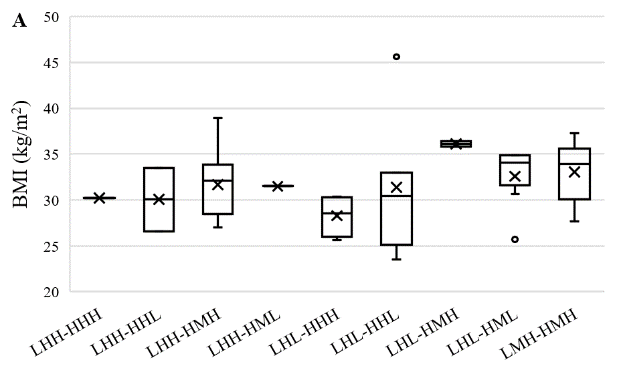

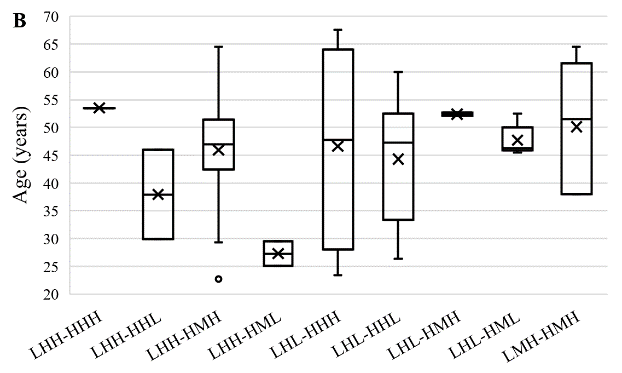


**
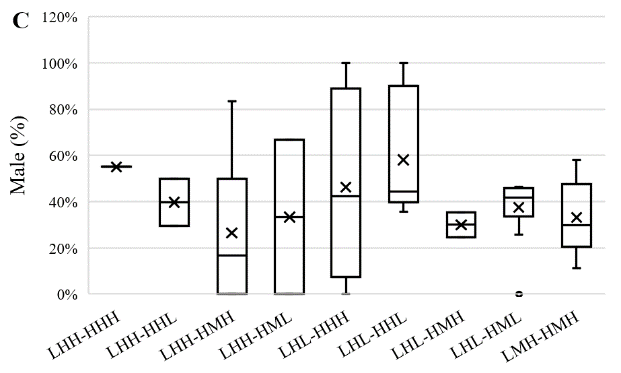

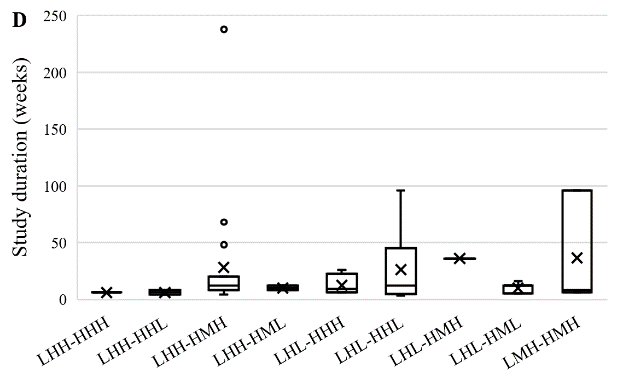
**

**
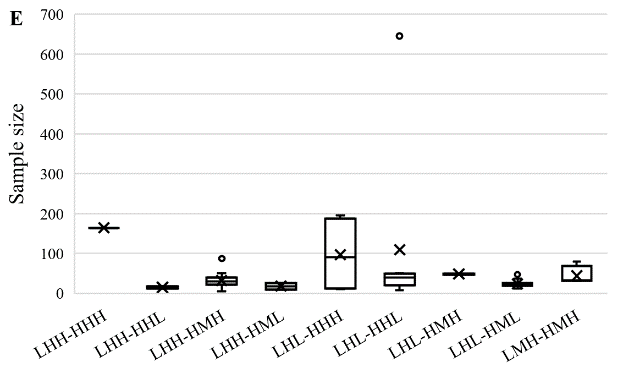
**

**Supplementary data**

# Supplementary Figure 6. Funnel plots for changes in SBP (A), DBP (B), TC (C), TG (D), LDL-C (E), HDL-C (F) and CRP (G) in standard meta-analysis for quantity-related studies. The dashed lines represent pseudo 95% confidence intervals.

# Egger’s test, A) *P-value* = 0.568; B) *P-value* = 0.751; C) *P-value* = 0.004; D) *P-value* = 0.435; E) *P-value* = 0.907; F) *P-value* = 0.405; G) *P-value* = 0.233


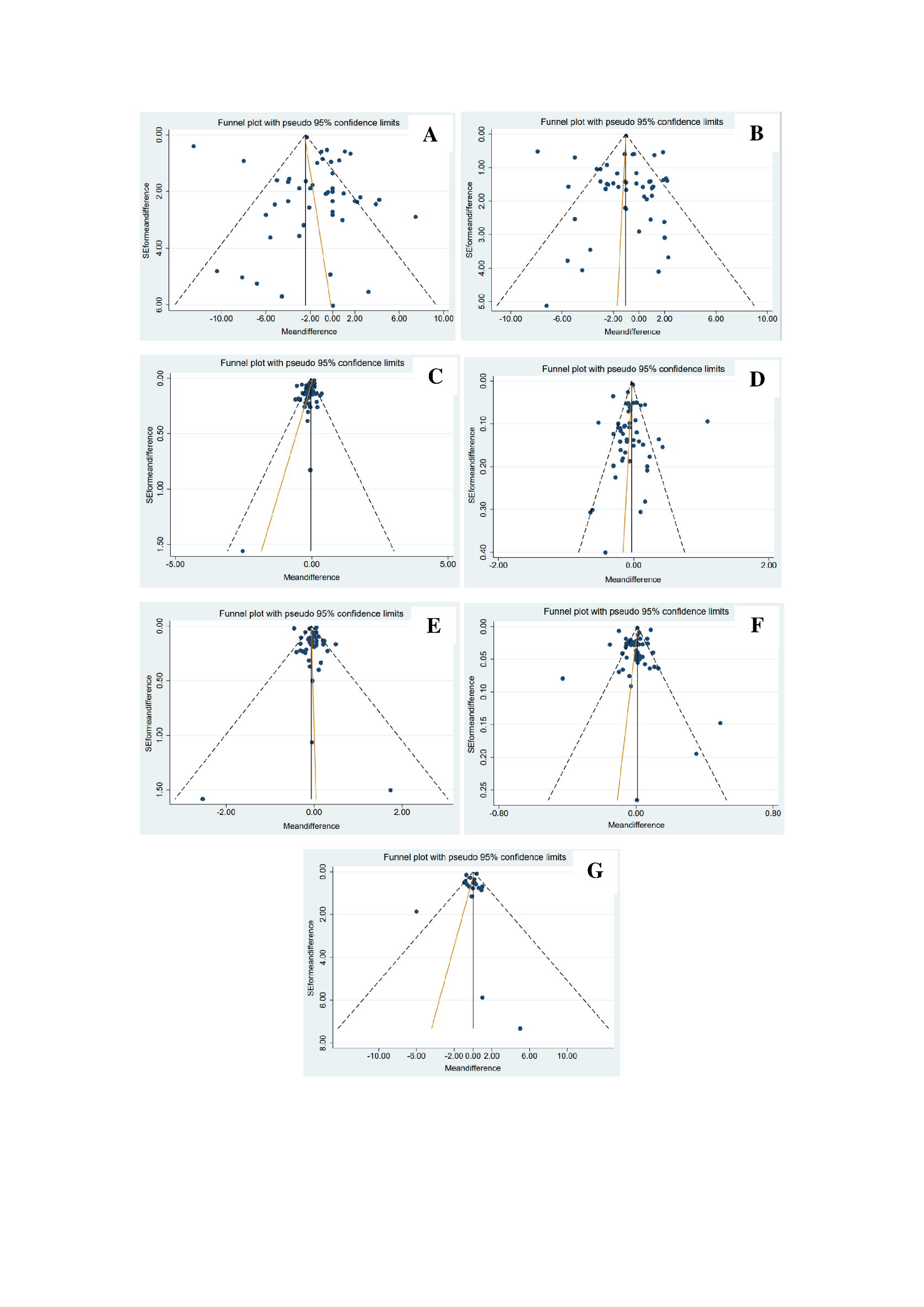


**Supplementary data**

# Supplementary Figure 7. Funnel plots for changes in SBP (A), DBP (B), TC (C), TG (D), LDL-C (E), HDL-C (F), and CRP (G) in standard meta-analysis for type-related studies. The dashed lines represent pseudo 95% confidence intervals.

# Egger’s test, A) *P-value* = 0.026; B) *P-value* = 0.002; C) *P-value* = 0.000; D) *P-value* = 0.232; E) *P-value* = 0.007; F) *P-value* = 0.320; G) *P-value* = 0.636


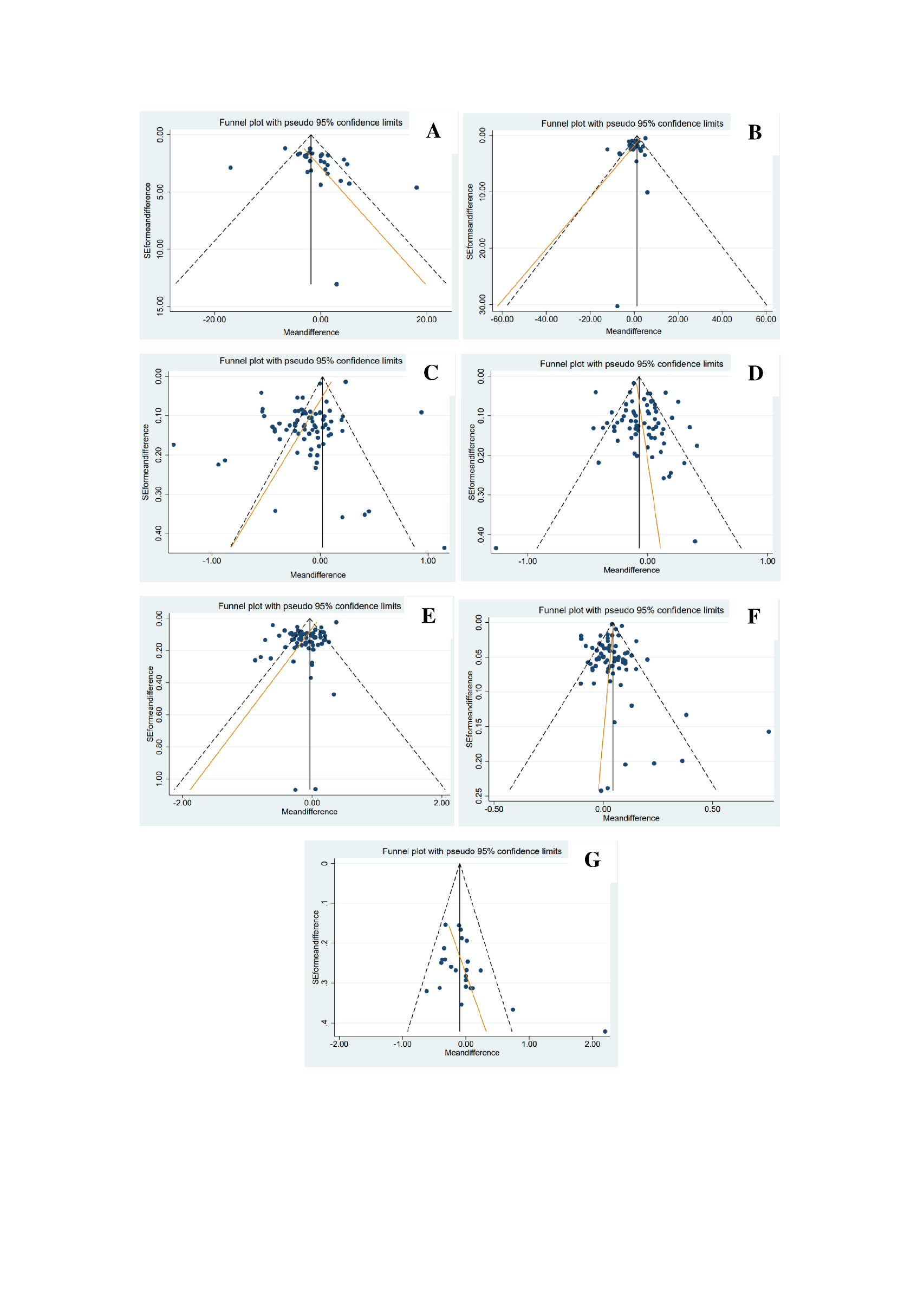


**Supplementary data**

# Supplementary Figure 8. Comparison-adjusted funnel plots for SBP (A), DBP (B), TC (C), TG (D), LDL-C (E), and HDL-C (F) including all studies of the network meta-analysis. The horizontal axis represents an adjusted effect size, presenting the difference between each observed effect size and the mean effect size for the specific comparison being made. The dashed lines represent pseudo 95% confidence intervals.


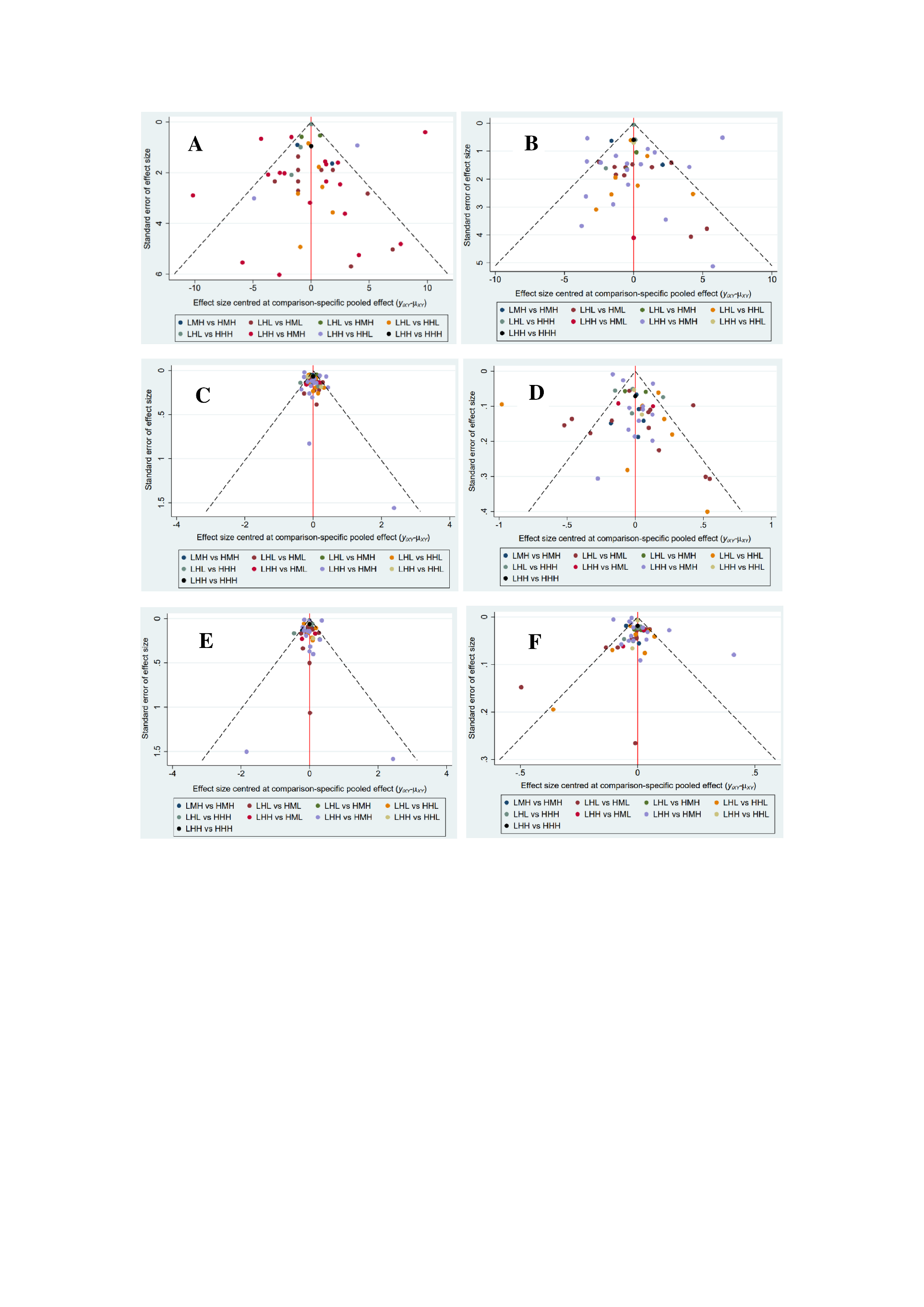


**Supplementary data**

**Supplementary references:**

1. Abete I, Parra D, Martinez JA Legume-, fish-, or high-protein-based hypocaloric diets: effects on weight loss and mitochondrial oxidation in obese men: United States, 2009.

2. Aldrich ND, Reicks MM, Sibley SD, Redmon JB, Thomas W, Raatz SK Varying protein source and quantity do not significantly improve weight loss, fat loss, or satiety in reduced energy diets among midlife adults, 2011.

3. Azadbakht L, Izadi V, Surkan PJ, Esmaillzadeh A. Effect of a high protein weight loss diet on weight, high-sensitivity C-reactive protein, and cardiovascular risk among overweight and obese women: A parallel clinical trial. Intl. J. Endocrinol. 2013;2013(

4. Bray GA, Redman LM, Rood J, de Jonge L, Smith SR. Effect of Overeating Dietary Protein at Different Levels on Circulating Lipids and Liver Lipid: the PROOF Study. Nutrients 2020;12(12).

5. Brinkworth G, Noakes M, Keogh J, Luscombe N, Wittert G, Clifton P. Long-term effects of a high-protein, low-carbohydrate diet on weight control and cardiovascular risk markers in obese hyperinsulinemic subjects. International journal of obesity and related metabolic disorders 2004;28(5):661.

6. Chiu S, Williams P, Dawson T, Bergman R, Stefanovski D, Watkins S, Krauss R. Diets High in Protein or Saturated Fat Do Not Affect Insulin Sensitivity or Plasma Concentrations of Lipids and Lipoproteins in Overweight and Obese Adults. JOURNAL OF NUTRITION 2014;144(11):1753.

7. Claessens M, van Baak M, Monsheimer S, Saris W The effect of a low-fat, high-protein or high-carbohydrate ad libitum diet on weight loss maintenance and metabolic risk factors: England, 2009.

8. Dalle Grave R, Calugi S, Gavasso I, El Ghoch M, Marchesini G. A randomized trial of energy-restricted high-protein versus high-carbohydrate, low-fat diet in morbid obesity. Obesity (Silver Spring, Md.) 2013;21(9):1774.

9. de Luis D, Izaola O, de la Fuente B, Primo D, Romero E. Role of Fatty Acid-Binding Protein 2 Ala54Thr Genotype on Weight Loss and Cardiovascular Risk Factors after a High-Protein/Low-Carbohydrate versus a Standard Hypocaloric Diet during 9 Months. Annals of nutrition & metabolism 2015;67(2):81.

10. Delbridge E, Prendergast L, Pritchard J, Proietto J. One-year weight maintenance after significant weight loss in healthy overweight and obese subjects: does diet composition matter? American journal of clinical nutrition 2009;90(5):1203.

11. Farnsworth E, Luscombe N, Noakes M, Wittert G, Argyiou E, Clifton P Effect of a high-protein, energy-restricted diet on body composition, glycemic control, and lipid concentrations in overweight and obese hyperinsulinemic men and women: United States, 2003.

12. Fekete A, Giromini C, Chatzidiakou Y, Givens I, Lovegrove J. The chronic consumption of milk proteins improve vascular function and lipid biomarkers in mildly hypertensive adults: results from the Whey2Go RCT. Proceedings of the Nutrition Society 2016;75(E171.

13. Fernandes R, Nabuco H, Sugihara J, Cavalcante E, Fabro P, Tomeleri C, Ribeiro A, Barbosa D, Venturini D, Schoenfeld Bet al. Effect of protein intake beyond habitual intakes following resistance training on cardiometabolic risk disease parameters in pre-conditioned older women. Experimental gerontology 2018;110(9.

14. Ferrara L, Innelli P, Palmieri V, Limauro S, De Luca G, Ferrara F, Liccardo E, Celentano A. Effects of different dietary protein intakes on body composition and vascular reactivity. European journal of clinical nutrition 2006;60(5):643.

15. Gögebakan Ö, Kohl A, Osterhoff MA, van Baak MA, Jebb SA, Papadaki A, Martinez JA, Handjieva-Darlenska T, Hlavaty P, Weickert MO. Effects of weight loss and long-term weight maintenance with diets varying in protein and glycemic index on cardiovascular risk factors: the diet, obesity, and genes (DiOGenes) study: a randomized, controlled trial. Circulation 2011;124(25):2829.

16. Gulati S, Misra A, Tiwari R, Sharma M, Pandey R, Yadav C. Effect of high-protein meal replacement on weight and cardiometabolic profile in overweight/obese Asian Indians in North India. British journal of nutrition 2017;117(11):1531.

17. He J, Wofford M, Reynolds K, Chen J, Chen C, Myers L, Minor D, Elmer P, Jones D, Whelton P. Effect of dietary protein supplementation on blood pressure: a randomized, controlled trial. Circulation 2011;124(5):589.

18. Hill A, Harris Jackson K, Roussell M, West S, Kris-Etherton P. Type and amount of dietary protein in the treatment of metabolic syndrome: a randomized controlled trial. American journal of clinical nutrition 2015;102(4):757.

19. Hodgson J, Burke V, Beilin L, Puddey I. Partial substitution of carbohydrate intake with protein intake from lean red meat lowers blood pressure in hypertensive persons. The American journal of clinical nutrition 2006;83(4):780.

20. Hodgson J, Zhu K, Lewis J, Kerr D, Meng X, Solah V, Devine A, Binns C, Woodman R, Prince R. Long-term effects of a protein-enriched diet on blood pressure in older women. The British journal of nutrition 2012;107(11):1664.

21. Hudson J, Zhou J, Kim J, Campbell W. Incorporating Milk Protein Isolate into an Energy-Restricted Western-Style Eating Pattern Augments Improvements in Blood Pressure and Triglycerides, but Not Body Composition Changes in Adults Classified as Overweight or Obese: A Randomized Controlled Trial. Nutrients 2020;12(3).

22. Jenkins D, Kendall C, Vidgen E, Vuksan V, Jackson C, Augustin L, Lee B, Garsetti M, Agarwal S, Rao Aet al. Effect of soy-based breakfast cereal on blood lipids and oxidized low-density lipoprotein: United States, 2000.

23. Johnston C, Tjonn S, Swan P. High-protein, low-fat diets are effective for weight loss and favorably alter biomarkers in healthy adults. Journal of nutrition 2004;134(3):586.

24. Juraschek S, Appel L, Anderson C, Miller E. Effect of a high-protein diet on kidney function in healthy adults: results from the OmniHeart trial. American journal of kidney diseases 2013;61(4):547.

25. Machin D, Park W, Alkatan M, Mouton M, Tanaka H. Hypotensive effects of solitary addition of conventional nonfat dairy products to the routine diet: a randomized controlled trial. American journal of clinical nutrition 2014;100(1):80.

26. Mamo J, James A, Soares M, Griffiths D, Purcell K, Schwenke J. A low-protein diet exacerbates postprandial chylomicron concentration in moderately dyslipidaemic subjects in comparison to a lean red meat protein-enriched diet. European journal of clinical nutrition 2005;59(10):1142.

27. Mateo-Gallego R, Marco-Benedí V, Perez-Calahorra S, Bea A, Baila-Rueda L, Lamiquiz-Moneo I, de Castro-Orós I, Cenarro A, Civeira F. Energy-restricted, high-protein diets more effectively impact cardiometabolic profile in overweight and obese women than lower-protein diets. Clinical nutrition (Edinburgh, Scotland) 2017;36(2):371.

28. McAuley K, Smith K, Taylor R, McLay R, Williams S, Mann J. Long-term effects of popular dietary approaches on weight loss and features of insulin resistance. International journal of obesity (2005) 2006;30(2):342.

29. Meckling K, Sherfey R. A randomized trial of a hypocaloric high-protein diet, with and without exercise, on weight loss, fitness, and markers of the Metabolic Syndrome in overweight and obese women. Physiologie appliquee, nutrition et metabolisme [Applied physiology, nutrition, and metabolism] 2007;32(4):743.

30. Mehrabani H, Salehpour S, Amiri Z, Farahani S, Meyer B, Tahbaz F. Beneficial effects of a high-protein, low-glycemic-load hypocaloric diet in overweight and obese women with polycystic ovary syndrome: a randomized controlled intervention study. Journal of the American College of Nutrition 2012;31(2):117.

31. Noakes M, Keogh J, Foster P, Clifton P. Effect of an energy-restricted, high-protein, low-fat diet relative to a conventional high-carbohydrate, low-fat diet on weight loss, body composition, nutritional status, and markers of cardiovascular health in obese women. American journal of clinical nutrition 2005;81(6):1298.

32. Papakonstantinou E, Triantafillidou D, Panagiotakos D, Koutsovasilis A, Saliaris M, Manolis A, Melidonis A, Zampelas A. A high-protein low-fat diet is more effective in improving blood pressure and triglycerides in calorie-restricted obese individuals with newly diagnosed type 2 diabetes. European journal of clinical nutrition 2010;64(6):595.

33. Pedersen E, Jesudason D, Clifton P. High protein weight loss diets in obese subjects with type 2 diabetes mellitus. Nutrition, metabolism, and cardiovascular diseases : NMCD 2014;24(5):554.

34. Porter S, Connelly M, Orenduff M, McDonald S, Sloane R, Huffman K, Kraus W, Bales C. Impact on cardiometabolic risk of a weight loss intervention with higher protein from lean red meat: combined results of 2 randomized controlled trials in obese middle-aged and older adults. Journal of clinical lipidology 2019;13(6):920.

35. Roussell M, Hill A, Gaugler T, West S, Heuvel J, Alaupovic P, Gillies P, Kris-Etherton P. Beef in an Optimal Lean Diet study: effects on lipids, lipoproteins, and apolipoproteins. The American journal of clinical nutrition 2012;95(1):9.

36. Sacks F, Wood P, Kass E Stability of blood pressure in vegetarians receiving dietary protein supplements: United States, 1984.

37. Sacks FM, Bray GA, Carey VJ, Smith SR, Ryan DH, Anton SD, McManus K, Champagne CM, Bishop LM, Laranjo N. Comparison of weight-loss diets with different compositions of fat, protein, and carbohydrates. New England Journal of Medicine 2009;360(9):859.

38. Sheikholeslami VD, Ahmadi KGF. Changes in antioxidant status and cardiovascular risk factors of overweight young men after six weeks supplementation of whey protein isolate and resistance training. Appetite 2012;59(3):673.

39. Stephenson T, Setchell K, Kendall C, Jenkins D, Anderson J, Fanti P. Effect of soy protein-rich diet on renal function in young adults with insulin-dependent diabetes mellitus. Clinical nephrology 2005;64(1):1.

40. Tang M, Armstrong CL, Leidy HJ, Campbell WW. Normal vs. high‐protein weight loss diets in men: effects on body composition and indices of metabolic syndrome. Obesity 2013;21(3):E204.

41. Teunisseti-Beekman KFM, Dopheide J, Geleijnse JM, Bakker SJL, Brink EJ, de Leeuw PW, van Baak MA. Protein supplementatio n lowers blood pressure in overweight adults: effect of dietary proteins on blood pressure (PROPRES), a randomized trial1. American Journal of Clinical Nutrition 2012;95(4):966.

42. Tischmann L, Drummen M, Joris P, Gatta-Cherifi B, Raben A, Fogelholm M, Matias I, Cota D, Mensink R, Westerterp-Plantenga Met al. Effects of a High-Protein Diet on Cardiometabolic Health, Vascular Function, and Endocannabinoids-A PREVIEW Study. Nutrients 2020;12(5).

43. Toscani M, Mario F, Radavelli-Bagatini S, Wiltgen D, Matos M, Spritzer P. Effect of high-protein or normal-protein diet on weight loss, body composition, hormone, and metabolic profile in southern Brazilian women with polycystic ovary syndrome: a randomized study. Gynecological endocrinology 2011;27(11):925.

44. Treyzon L, Chen S, Hong K, Yan E, Carpenter C, Thames G, Bowerman S, Wang H, Elashoff R, Li Z. A controlled trial of protein enrichment of meal replacements for weight reduction with retention of lean body mass. Nutrition journal 2008;7(23.

45. Weinheimer EM, Conley TB, Kobza VM, Sands LP, Lim E, Janle EM, Campbell WW. Whey protein supplementation does not affect exercise training-induced changes in body composition and indices of metabolic syndrome in middle-aged overweight and obese adults. The Journal of nutrition 2012;142(8):1532.

46. Wolfe B, Giovannetti P Short-term effects of substituting protein for carbohydrate in the diets of moderately hypercholesterolemic human subjects: United States, 1991.

47. Wolfe B, Giovannetti P High protein diet complements resin therapy of familial hypercholesterolemia: Canada, 1992.

48. Wolfe B, Piché L. Replacement of carbohydrate by protein in a conventional-fat diet reduces cholesterol and triglyceride concentrations in healthy normolipidemic subjects. Clinical and investigative medicine. Medecine clinique et experimentale 1999;22(4):140.

49. Yılmaz SK, Eskici G, Mertoğlu C, Ayaz A. Effect of different protein diets on weight loss, inflammatory markers, and cardiometabolic risk factors in obese women. Journal of Research in Medical Sciences 2021, DOI:doi:10.4103/jrms.JRMS_611_20 doi:10.4103/jrms.JRMS_611_20:1.

50. Anderson J, Fuller J, Patterson K, Blair R, Tabor A Soy compared to casein meal replacement shakes with energy-restricted diets for obese women: randomized controlled trial: United States, 2007.

51. Ashton E, Ball M. Effects of soy as tofu vs meat on lipoprotein concentrations. European journal of clinical nutrition 2000;54(1):14.

52. Azadbakht L, Shakerhosseini R, Atabak S, Jamshidian M, Mehrabi Y, Esmaill-Zadeh A Beneficiary effect of dietary soy protein on lowering plasma levels of lipid and improving kidney function in type II diabetes with nephropathy: England, 2003.

53. Azadbakht L, Atabak S, Esmaillzadeh A. Soy protein intake, cardiorenal indices, and C-reactive protein in type 2 diabetes with nephropathy: a longitudinal randomized clinical trial. Diabetes care 2008;31(4):648.

54. Azadbakht L, Nurbakhsh S. Effect of soy drink replacement in a weight reducing diet on anthropometric values and blood pressure among overweight and obese female youths. Asia Pacific journal of clinical nutrition 2011;20(3):383.

55. Bahr M, Fechner A, Kramer J, Kiehntopf M, Jahreis G. Lupin protein positively affects plasma LDL cholesterol and LDL:HDL cholesterol ratio in hypercholesterolemic adults after four weeks of supplementation: a randomized, controlled crossover study. NUTRITION JOURNAL 2013;12(

56. Bähr M, Fechner A, Kiehntopf M, Jahreis G. Consuming a mixed diet enriched with lupin protein beneficially affects plasma lipids in hypercholesterolemic subjects: a randomized controlled trial. Clinical nutrition (Edinburgh, Scotland) 2015;34(1):7.

57. Bakhit R, Klein B, Essex-Sorlie D, Ham J, Erdman JJ, Potter S Intake of 25 g of soybean protein with or without soybean fiber alters plasma lipids in men with elevated cholesterol concentrations: United States, 1994.

58. Basciani S, Camajani E, Contini S, Persichetti A, Risi R, Bertoldi L, Strigari L, Prossomariti G, Watanabe M, Mariani Set al. Very-low-calorie ketogenic diets with whey, vegetable, or animal protein in patients with obesity: A randomized pilot study. J. Clin. Endocrinol. Metab. 2020;105(9):2939.

59. Beavers K, Serra M, Beavers D, Hudson G, Willoughby D. The lipid-lowering effects of 4 weeks of daily soymilk or dairy milk ingestion in a postmenopausal female population. Journal of medicinal food 2010;13(3):650.

60. Bergeron N, Chiu S, Williams P, King S, Krauss R. Effects of red meat, white meat, and nonmeat protein sources on atherogenic lipoprotein measures in the context of low compared with high saturated fat intake: a randomized controlled trial. The American journal of clinical nutrition 2019;110(1):24.

61. Borodin E, Menshikova I, Dorovskikh V, Feoktistova N, Shtarberg M, Yamamoto T, Takamatsu K, Mori H, Yamamoto S. Effects of two-month consumption of 30 g a day of soy protein isolate or skimmed curd protein on blood lipid concentration in Russian adults with hyperlipidemia. Journal of nutritional science and vitaminology 2009;55(6):492.

62. Bosello O, Cominacini L, Zocca I, Garbin U, Compri R, Davoli A, Brunetti L. Short- and long-term effects of hypocaloric diets containing proteins of different sources on plasma lipids and apoproteins of obese subjects. Annals of nutrition & metabolism 1988;32(4):206.

63. Bricarello L, Kasinski N, Bertolami M, Faludi A, Pinto L, Relvas W, Izar M, Ihara S, Tufik S, Fonseca F Comparison between the effects of soy milk and non-fat cow milk on lipid profile and lipid peroxidation in patients with primary hypercholesterolemia: United States, 2004.

64. Campbell S, Khalil D, Payton M, Arjmandi B. One-year soy protein supplementation does not improve lipid profile in postmenopausal women. Menopause (New York, N.Y.) 2010;17(3):587.

65. Chen S, Ferng S, Yang C, Peng S, Lee H, Chen J Variable effects of soy protein on plasma lipids in hyperlipidemic and normolipidemic hemodialysis patients: United States, 2005.

66. Chen S, Chen J, Yang C, Peng S, Ferng S. Effect of soya protein on serum lipid profile and lipoprotein concentrations in patients undergoing hypercholesterolaemic haemodialysis. The British journal of nutrition 2006;95(2):366.

67. Crimarco A, Springfield S, Petlura C, Streaty T, Cunanan K, Lee J, Fielding-Singh P, Carter M, Topf M, Wastyk Het al. A randomized crossover trial on the effect of plant-based compared with animal-based meat on trimethylamine-N-oxide and cardiovascular disease risk factors in generally healthy adults: study With Appetizing Plantfood-Meat Eating Alternative Trial (SWAP-MEAT). American journal of clinical nutrition 2020;112(5):1188.

68. Cuevas A, Irribarra V, Castillo O, Yañez M, Germain A Isolated soy protein improves endothelial function in postmenopausal hypercholesterolemic women: England, 2003.

69. Desroches S, Mauger J, Ausman L, Lichtenstein A, Lamarche B Soy protein favorably affects LDL size independently of isoflavones in hypercholesterolemic men and women: United States, 2004.

70. Frota KM, dos Santos FR, Ribeiro V, Arêas J. Cowpea protein reduces LDL-cholesterol and apolipoprotein B concentrations, but does not improve biomarkers of inflammation or endothelial dysfunction in adults with moderate hypercholesterolemia. Nutricion hospitalaria 2015;31(4):1611.

71. Gardner C, Newell K, Cherin R, Haskell W The effect of soy protein with or without isoflavones relative to milk protein on plasma lipids in hypercholesterolemic postmenopausal women: United States, 2001.

72. Gardner C, Messina M, Kiazand A, Morris J, Franke A. Effect of two types of soy milk and dairy milk on plasma lipids in hypercholesterolemic adults: a randomized trial. Journal of the American College of Nutrition 2007;26(6):669.

73. George K, Muñoz J, Akhavan N, Foley E, Siebert S, Tenenbaum G, Khalil D, Chai S, Arjmandi B. Is soy protein effective in reducing cholesterol and improving bone health? Food & function 2020;11(1):544.

74. Jamilian M, Asemi Z. The Effect of Soy Intake on Metabolic Profiles of Women With Gestational Diabetes Mellitus. The Journal of clinical endocrinology and metabolism 2015;100(12):4654.

75. Jassi HK, Jain A, Arora S, Chitra R. Effect of soy proteins Vs soy isoflavones on lipid profile in postmenopausal women. Indian J. Clin. Biochem. 2010;25(2):201.

76. Jenkins D, Kendall C, Garsetti M, Rosenberg-Zand R, Jackson C, Agarwal S, Rao A, Diamandis E, Parker T, Faulkner Det al. Effect of soy protein foods on low-density lipoprotein oxidation and ex vivo sex hormone receptor activity--a controlled crossover trial: United States, 2000.

77. Karamali M, Kashanian M, Alaeinasab S, Asemi Z. The effect of dietary soy intake on weight loss, glycaemic control, lipid profiles and biomarkers of inflammation and oxidative stress in women with polycystic ovary syndrome: a randomised clinical trial. Journal of human nutrition and dietetics : the official journal of the British Dietetic Association 2018;31(4):533.

78. Kurowska E, Jordan J, Spence J, Wetmore S, Piché L, Radzikowski M, Dandona P, Carroll K Effects of substituting dietary soybean protein and oil for milk protein and fat in subjects with hypercholesterolemia: Canada, 1997.

79. Liao F, Shieh M, Yang S, Lin S, Chien Y. Effectiveness of a soy-based compared with a traditional low-calorie diet on weight loss and lipid levels in overweight adults. Nutrition (Burbank, Los Angeles County, Calif.) 2007;23(7):551.

80. Liu Z, Ho S, Chen Y, Ho Y. The effects of isoflavones combined with soy protein on lipid profiles, C-reactive protein and cardiovascular risk among postmenopausal Chinese women. Nutrition, metabolism, and cardiovascular diseases : NMCD 2012;22(9):712.

81. Liu Z, Ho S, Chen Y, Woo J. Effect of soy protein and isoflavones on blood pressure and endothelial cytokines: a 6-month randomized controlled trial among postmenopausal women. Journal of hypertension 2013;31(2):384.

82. Liu Z, Ho S, Chen Y, Ho S, To K, Tomlinson B, Woo J. Whole soy, but not purified daidzein, had a favorable effect on improvement of cardiovascular risks: a 6-month randomized, double-blind, and placebo-controlled trial in equol-producing postmenopausal women. Molecular nutrition & food research 2014;58(4):709.

83. Ma L, Grann K, Li M, Jiang Z. A pilot study to evaluate the effect of soy isolate protein on the serum lipid profile and other potential cardiovascular risk markers in moderately hypercholesterolemic Chinese adults. Ecology of food and nutrition 2011;50(6):473.

84. Maki K, Butteiger D, Rains T, Lawless A, Reeves M, Schasteen C, Krul E. Effects of soy protein on lipoprotein lipids and fecal bile acid excretion in men and women with moderate hypercholesterolemia. Journal of clinical lipidology 2010;4(6):531.

85. Matthan N, Jalbert S, Ausman L, Kuvin J, Karas R, Lichtenstein A. Effect of soy protein from differently processed products on cardiovascular disease risk factors and vascular endothelial function in hypercholesterolemic subjects. The American journal of clinical nutrition 2007;85(4):960.

86. McVeigh B, Dillingham B, Lampe J, Duncan A. Effect of soy protein varying in isoflavone content on serum lipids in healthy young men. The American journal of clinical nutrition 2006;83(2):244.

87. Meinertz H, Faergeman O, Nilausen K, Chapman M, Goldstein S, Laplaud P. Effects of soy protein and casein in low cholesterol diets on plasma lipoproteins in normolipidemic subjects. Atherosclerosis 1988;72(1):63.

88. Meinertz H, Nilausen K, Faergeman O Soy protein and casein in cholesterol-enriched diets: effects on plasma lipoproteins in normolipidemic subjects: United States, 1989.

89. Nilausen K, Meinertz H Variable lipemic response to dietary soy protein in healthy, normolipemic men: United States, 1998.

90. Padhi E, Blewett H, Duncan A, Guzman R, Hawke A, Seetharaman K, Tsao R, Wolever T, Ramdath D. Whole Soy Flour Incorporated into a Muffin and Consumed at 2 Doses of Soy Protein Does Not Lower LDL Cholesterol in a Randomized, Double-Blind Controlled Trial of Hypercholesterolemic Adults. The Journal of nutrition 2015;145(12):2665.

91. Pipe E, Gobert C, Capes S, Darlington G, Lampe J, Duncan A. Soy protein reduces serum LDL cholesterol and the LDL cholesterol:HDL cholesterol and apolipoprotein B:apolipoprotein A-I ratios in adults with type 2 diabetes. The Journal of nutrition 2009;139(9):1700.

92. Prescott S, Jenner D, Beilin L, Margetts B, Vandongen R Controlled study of the effects of dietary protein on blood pressure in normotensive humans: Australia, 1987.

93. Rivas M, Garay R, Escanero J, Cia P, Cia P, Alda J Soy milk lowers blood pressure in men and women with mild to moderate essential hypertension: United States, 2002.

94. Santo A, Santo A, Browne R, Burton H, Leddy J, Horvath S, Horvath P. Postprandial lipemia detects the effect of soy protein on cardiovascular disease risk compared with the fasting lipid profile. Lipids 2010;45(12):1127.

95. Shidfar F, Ehramphosh E, Heydari I, Haghighi L, Hosseini S, Shidfar S. Effects of soy bean on serum paraoxonase 1 activity and lipoproteins in hyperlipidemic postmenopausal women. International journal of food sciences and nutrition 2009;60(3):195.

96. Shige H, Ishikawa T, Higashi K, Yamashita T, Tomiyasu K, Yoshida H, Hosoai H, Ito T, Nakajima K, Ayaori Met al. Effects of soy protein isolate (SPI) and casein on the postprandial lipemia in normolipidemic men. Journal of nutritional science and vitaminology 1998;44(1):113.

97. Sirtori C, Triolo M, Bosisio R, Bondioli A, Calabresi L, de Vergori V, Gomaraschi M, Mombelli G, Pazzucconi F, Zacherl Cet al. Hypocholesterolaemic effects of lupin protein and pea protein/fibre combinations in moderately hypercholesterolaemic individuals. The British journal of nutrition 2012;107(8):1176.

98. Sucher S, Markova M, Hornemann S, Pivovarova O, Rudovich N, Thomann R, Schneeweiss R, Rohn S, Pfeiffer A. Comparison of the effects of diets high in animal or plant protein on metabolic and cardiovascular markers in type 2 diabetes: a randomized clinical trial. Diabetes, obesity & metabolism 2017;19(7):944.

99. Tabibi H, Imani H, Hedayati M, Atabak S, Rahmani L Effects of soy consumption on serum lipids and apoproteins in peritoneal dialysis patients: a randomized controlled trial: United States, 2010.

100. Tahavorgar A, Vafa M, Shidfar F, Gohari M, Heydari I. Beneficial effects of whey protein preloads on some cardiovascular diseases risk factors of overweight and obese men are stronger than soy protein preloads - A randomized clinical trial. J. Nutr. Intermediary Metab. 2015;2(3):69.

101. Teede H, Dalais F, Kotsopoulos D, Liang Y, Davis S, McGrath B Dietary soy has both beneficial and potentially adverse cardiovascular effects: a placebo-controlled study in men and postmenopausal women: United States, 2001.

102. Teixeira S, Potter S, Weigel R, Hannum S, Erdman J, Hasler C. Effects of feeding 4 levels of soy protein for 3 and 6 wk on blood lipids and apolipoproteins in moderately hypercholesterolemic men. AMERICAN JOURNAL OF CLINICAL NUTRITION 2000;71(5):1077.

103. Tonstad S, Smerud K, Høie L A comparison of the effects of 2 doses of soy protein or casein on serum lipids, serum lipoproteins, and plasma total homocysteine in hypercholesterolemic subjects: United States, 2002.

104. van Raaij J, Katan M, Hautvast J, Hermus R Effects of casein versus soy protein diets on serum cholesterol and lipoproteins in young healthy volunteers: United States, 1981.

105. van Raaij J, Katan M, West C, Hautvast J Influence of diets containing casein, soy isolate, and soy concentrate on serum cholesterol and lipoproteins in middle-aged volunteers: United States, 1982.

106. Vigna G, Pansini F, Bonaccorsi G, Albertazzi P, Donegà P, Zanotti L, de Aloysio D, Mollica G, Fellin R Plasma lipoproteins in soy-treated postmenopausal women: a double-blind, placebo-controlled trial: Netherlands, 2000.

107. Wang M, Yamamoto S, Chung H, Chung S, Miyatani S, Mori M, Okita T, Sugano M Antihypercholesterolemic effect of undigested fraction of soybean protein in young female volunteers: Japan, 1995.

108. Weisse K, Brandsch C, Zernsdorf B, Nkengfack N, Hofmann K, Eder K, Stangl G Lupin protein compared to casein lowers the LDL cholesterol:HDL cholesterol-ratio of hypercholesterolemic adults: Germany, 2010.

109. Wiebe S, Bruce V, McDonald B A comparison of the effect of diets containing beef protein and plant proteins on blood lipids of healthy young men: United States, 1984.

110. Xu C, Wang X-H, Guo L-Y, Zhou B. Effect of soybean protein in lowering human serum cholesterol. Chin. J. Clin. Rehab. 2005;9(43):167.
